# Sclerites of Bursa Copulatrix Reveal Hidden Generic Diversity in Dimini (Coleoptera, Elateridae): Revision of *Parapenia* Species from China, with the Establishment of Three New Genera

**DOI:** 10.3390/insects16101003

**Published:** 2025-09-26

**Authors:** Lu Qiu, Yongying Ruan, Alexander S. Prosvirov, Robin Kundrata

**Affiliations:** 1 School of Life Sciences (School of Ecological Forestry), Mianyang Teachers’ College, Mianxing West Road, Mianyang 621000, China; 2 Plant Protection Research Center, Shenzhen Polytechnic University, Shenzhen 518055, China; yongyingruan@hotmail.com; 3 Department of Entomology, Faculty of Biology, Moscow State University, Leninskie Gory 1/12, 119234 Moscow, Russia; carrabus69@mail.ru; 4 Department of Zoology, Faculty of Science, Palacky University, 17. listopadu 50, 77146 Olomouc, Czech Republic

**Keywords:** Elateroidea, Dendrometrinae, click beetles, diversity, new genus, Oriental region

## Abstract

The genus *Parapenia* Suzuki from China is revised, with 12 species recognized, including six new species: *P. fuxi* **sp. nov.**, *P. nyuwa* **sp. nov.**, *P. pangu* **sp. nov.**, *P. ruihangi* **sp. nov.**, *P. wuchaoi* **sp. nov.**, and *P. zhengi* **sp. nov.** In addition, *P. sausai* Schimmel and *P. villosa* (Fleutiaux) are newly recorded from China, and *P. jagemanni* Schimmel **syn. nov.** is synonymized with *P. yunnana* Schimmel. Large plate-like sclerites in the bursa copulatrix are consistently present in *Parapenia* and show diagnostic value for generic delimitation. Comparative morphological studies, particularly of these sclerites, justify the establishment of three new genera distinct from *Parapenia*: (1) *Parapenioides* **gen. nov.**, including *P. zidani* **sp. nov.**; (2) *Megapenia* **gen. nov.**, including *M. cruciata* (Bouwer) **comb. nov.**, type species *M. marginalis* (Fleutiaux) **comb. nov.**, and *M. tianlongi* **sp. nov.**; and (3) *Sinopenia* **gen. nov.**, including *S. significata* (Schimmel) **comb. nov.** A comparative diagnostic table of the four genera, together with a checklist and distribution maps, is provided. Identification keys to Chinese species of *Parapenia* and *Megapenia* are also included. This study clarifies the taxonomic complexity of *Parapenia* and emphasizes the overlooked significance of sclerotized structures in the bursa copulatrix for distinguishing genera within Dimini.

## Simple Summary

In this study, we revised the click-beetle genus *Parapenia* from China. We recognized 12 Chinese species in total, including six that are newly discovered. Two species are reported from China for the first time, and one species is treated as a synonym. A detailed study of female reproductive structures revealed plate-like structures that had rarely been noticed before in this group of beetles. These structures proved to be very reliable for identifying and separating different taxonomic groups. Based on these findings, three new genera morphologically similar to *Parapenia* were established. To support future work, we provided a comparative diagnostic table, distribution maps, and identification keys for these beetles in China. This research not only clarifies the true diversity of *Parapenia* but also demonstrates that overlooked internal structures can be essential for understanding the generic limits and how these click-beetle groups are related to each other, which is important for documenting and protecting biodiversity.

## 1. Introduction

*Parapenia* Suzuki, 1982 (Coleoptera, Elateridae, Dendrometrinae) is a relatively small but morphologically distinctive genus of the tribe Dimini [1,2,3]. It can be readily distinguished from other Dimini genera by its unique pronotal structure, characterized by emarginate (=bidentate) apex of posterior angles and prominently narrowed, protruding anterior angles [1]. Species of this genus are predominantly distributed in the northern Oriental Region, with records from China, India, Laos, Myanmar, Thailand, and Vietnam [3]. The genus remained poorly defined until study of Suzuki [1], as its constituent species originally described in other genera: *Parapenia villosa* (Fleutiaux, 1936) and *P. tonkinensis* (Fleutiaux, 1918) in *Penia* Laporte, 1838 [4,5], and *P. taiwana* (Miwa, 1930) in *Csikia* Szombathy, 1910 [6]. Suzuki [1] established *Parapenia* to accommodate these three species and described three additional ones from Thailand and India (Assam). Subsequent contributions by Schimmel [7,8,9,10,11] further expanded the genus, with additional seven species described from China, India, and Laos, and *Penia marginalis* Fleutiaux, 1942 transferred to *Parapenia* [12]. Prior to the present study, the genus comprised 14 valid species, seven of which were recorded from China [3].

Recent taxonomic studies have increasingly highlighted China as a major biodiversity hotspot for Dimini beetles, with numerous new taxa and distributional records being documented [13,14,15,16,17,18,19,20,21,22,23,24,25]. China harbors half of all known *Parapenia* species; however, the genus remains poorly understood due to incomplete original descriptions and insufficient diagnostic illustrations. This study presents the first comprehensive revisionary study for Chinese *Parapenia*, recognizing 12 species, including six new species, two new country records, and one new synonym. In addition, three new genera are established for species previously misassigned to *Parapenia* or superficially similar to it. Notably, large plate-like sclerites in the bursa copulatrix, rarely found in other Dimini but present in all four genera treated here, are shown to have diagnostic value at the generic level. These findings establish a robust morphological framework for *Parapenia* and its allied genera from China and neighboring regions, offering a solid basis for future phylogenetic and biogeographic studies of Asian Dimini.

## 2. Materials and Methods

Specimens were softened in hot water, after which the genital segments were excised and dissected following treatment in 10% KOH, heated to 70–80 °C for 10–15 min. Habitus images were captured using a Canon EOS RP camera (Canon Inc., Tokyo, Japan) with an EF-EOS R mount adapter (Canon Inc., Taipei City, Taiwan, China) and a Laowa 100 mm F2.8 CA-Dreamer Macro 2× lens (Changgeng Optics Technology Co., Ltd, Anhui, China). Diagnostic characters were photographed using the same camera setup, either with a Laowa 25 mm F2.8 2.5–5× Ultra Macro Lens (Changgeng Optics Technology Co., Ltd, Anhui, China) or in combination with a Mitutoyo M Plan Apo 10×/0.28 lens (Mitutoyo Co., Kanagawa, Japan). All figures (Figure 1, Figure 2, Figure 3, Figure 4, Figure 5, Figure 6, Figure 7, Figure 8, Figure 9, Figure 10, Figure 11, Figure 12, Figure 13, Figure 14, Figure 15, Figure 16, Figure 17, Figure 18, Figure 19, Figure 20, Figure 21, Figure 22, Figure 23, Figure 24, Figure 25, Figure 26, Figure 27, Figure 28, Figure 29, Figure 30, Figure 31, Figure 32, Figure 33, Figure 34, Figure 35, Figure 36, Figure 37, Figure 38, Figure 39 and Figure 40) were edited in Adobe Photoshop CC 2019. To facilitate the observation of punctation on the pronotum, part of the pubescence was removed from the pronotum for some specimens (Figure 3D, Figure 5C,D, Figure 7C, Figure 9E, Figure 11A, Figure 12E, Figure 15A, Figure 18A, Figure 20D, Figure 21H, Figure 24D, Figure 26C,D, Figure 28G and Figure 33A). The distribution map (Figure 38, Figure 39 and Figure 40) was generated using QGIS 3.42.3-Bratislava.

**Figure 1:**
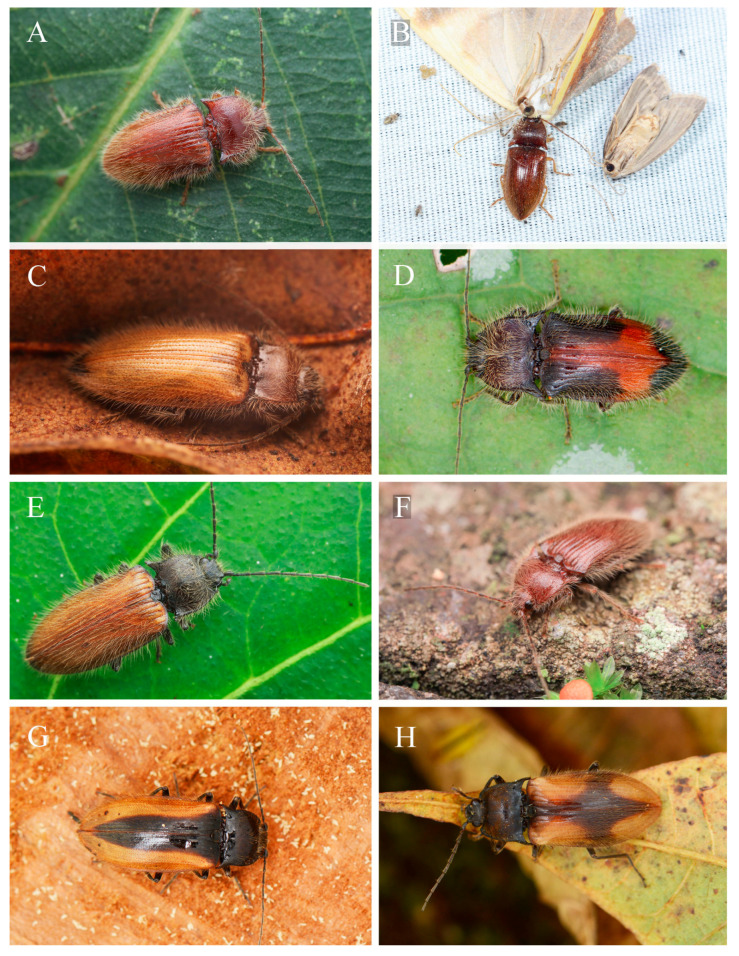
Living individuals of *Parapenia* and *Megapenia* **gen. nov.** species from China. (**A**) *Parapenia villosa* (Guangxi, Napo); (**B**) *P. wulingshanensis* (Guangdong, Nanling Mountain); (**C**) *P. pangu* **sp. nov.** (Yunnan, Bingzhongluo); (**D**) *P. wuchaoi* **sp. nov.** (Yunnan, Dulongjiang); (**E**) *P. nyuwa* **sp. nov.** (Yunnan, Ailaoshan Mountain); (**F**) *P. tonkinensis* (Sichuan, Tangjiahe N.R.); (**G**) *Megapenia marginalis* **comb. nov.** (Yunnan, Pianma); (**H**) *M. cruciata* **comb. nov.** (Yunnan, Dulongjiang). (**B**) photographed by Guo-Cong Huang; (**D**,**G**,**H**) by Chao Wu; the rest by Lu Qiu.

Body length was measured from the anterior margin of the head to the apex of the elytra. Pronotal length was taken along the midline; pronotal width at the posterior angles; and body width at the broadest point of the elytra. The generic concept of Parapenia follows Suzuki [1] and Kundrata et al. [3]. Historical locality names from Taiwan Province are referenced according to Chu & Yamanaka [26].

Specimens examined in this study are deposited in the following collections:

CWNU—College of Life Sciences, China West Normal University, Nanchong, Sichuan, China
DEMSU—Department of Entomology, Lomonosov Moscow State University, Moscow, Russia
DLU—Dali University, Dali, Yunnan, China
HBUM—Hebei University Museum, Baoding, Hebei, China
HNHM—Hungarian Natural History Museum, Budapest, Hungary
IZCAS—Institute of Zoology, Chinese Academy of Sciences, Beijing, China
MNHN—Muséum National d’Histoire Naturelle, Paris, France
MYTC (MYNU)—Invertebrate Collection of Mianyang Teachers’ College, Mianyang, Sichuan, China
MZMB—Moravské zemské muzeum, Brno, Czech Republic
NHMUK—Natural History Museum, London, United Kingdom
NHMW—Natural History Museum Vienna, Vienna, Austrian
PCJHo—collection of Jan Horák, Praha, Czech Republic
PCRK—collection of Robin Kundrata, Olomouc, Czech Republic
SZPU (SZPT)—Plant Protection Research Center, Shenzhen Polytechnic University, Shenzhen, Guangdong, China
TARI—Taiwan Agricultural Research Institute, Taichung, Taiwan, China
ZISP—Zoological Institute of the Russian Academy of Sciences, St. Petersburg, Russia

Although not illustrated in this paper, photographs of the holotypes of *Parapenia nigroapicalis* Suzuki, 1982 and *P. thailandica* Suzuki, 1982 (provided by W. Suzuki), and *P. spicula* Schimmel, 2001 (provided by K. Matsumoto and M. Geiser), were examined for comparative purposes.

For most specimens, collection data are originally in Chinese and provided here in English. For type specimens and certain material deposited in HNHM, NHMUK, NHMW, PCJHo, PCRK, and ZISP, original labels are transcribed verbatim and enclosed in quotation marks (“”), with commas (,) indicating different labels, and slashes (/) indicating different lines on the same labels. Chinese translations or supplementary notes are provided in square brackets ([]). The ZooBank LSID number for this publication is: urn:lsid:zoobank.org:pub:DAADD908-70D0-43D7-AF95-B8C107A5036B.

## 3. Results

### 3.1. Revision of Parapenia Species from China

#### *Genus Parapenia* Suzuki, 1982

(Figure 1A–F, Figure 2, Figure 3, Figure 4, Figure 5, Figure 6, Figure 7, Figure 8, Figure 9, Figure 10, Figure 11, Figure 12, Figure 13, Figure 14, Figure 15, Figure 16, Figure 17, Figure 18, Figure 19, Figure 20, Figure 21, Figure 22, Figure 23, Figure 24, Figure 25A–C, Figure 38 and Figure 39)

*Parapenia* Suzuki, 1982: 84 [1] (original description); Schimmel & Platia 1991: 306 [27] (diagnosis); Schimmel 1996: 157 [2] (diagnosis); Suzuki 1999: 121 [28] (catalogue); Cate 2007: 184 [29] (catalogue); Kundrata et al. 2018: 31 [3] (catalogue); Kundrata et al., 2018: 275 [30] (remarks); Jiang & Yang 2023: 45 [31] (catalogue).

**Type species.** *Parapenia nigroapicalis* Suzuki, 1982, by original designation.

**Figure 2:**
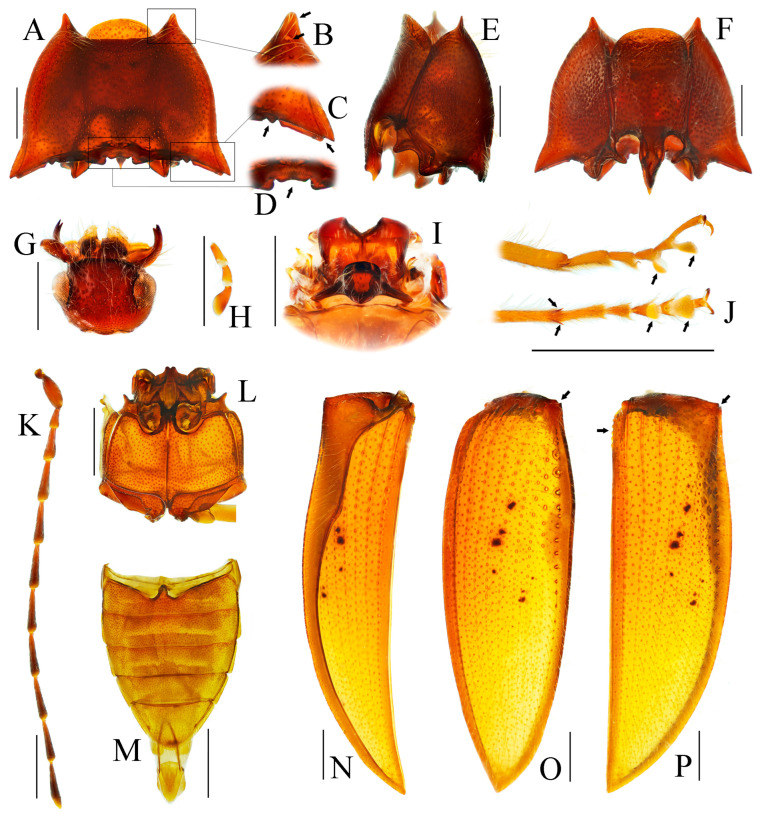
Characters of genus *Parapenia* (*P. wulingshanensis*, male from Guangdong, Nanling Mountain). (**A**–**F**) Pronotum: (**A**) dorsal view; (**B**) anterior angle of pronotum (upper arrow shows the tapered process, lower arrow shows the pit at the end of the process); (**C**) hind angle of pronotum (left arrow shows the two dents near the end of posterior margin of hind angle, right arrow shows the bidentate hind angle); (**D**) the M-shaped notch at posterior margin of pronotum (arrowed); (**E**) lateral view; (**F**) ventral view; (**G**) head; (**H**) Maxillary palpi II–IV; (**I**) mesonotum; (**J**) tarsi (left two arrows show the two spines at distal of tibia, right four arrows show the two lobes at tarsomeres III–IV); (**K**) antenna; (**L**) meso- and metathorax; (**M**) abdomen; (**N**–**P**) elytron: (**N**) lateral view; (**O**) dorso-lateral view (arrow shows the bidentate anterior angle); (**P**) dorsal view (left arrow shows the serrated suture base of elytron, right arrow shows the bidentate anterior angle). Scale bars 1 mm, (**B**–**D**) not to scale.

**Figure 3:**
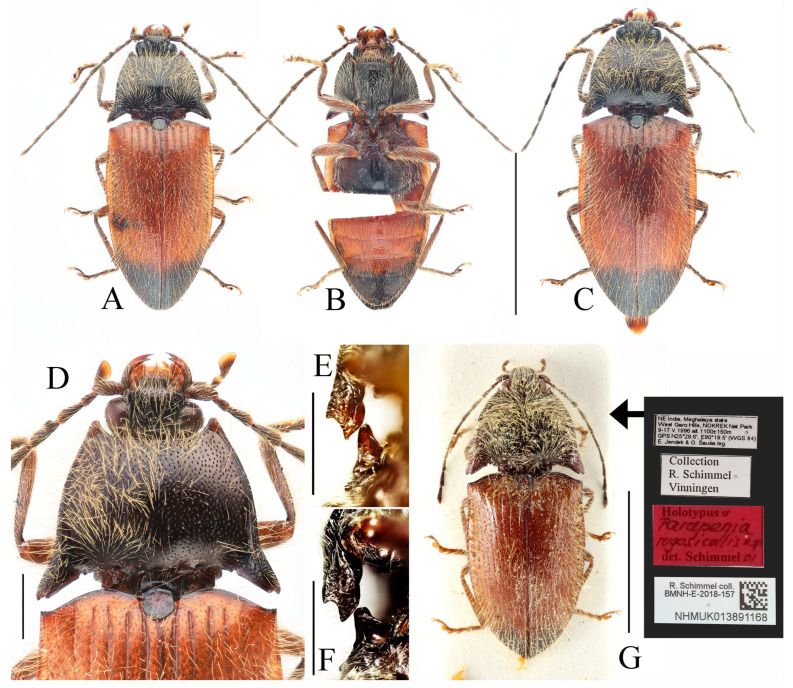
(**A**–**F**) *Parapenia fuxi* sp. nov. from Yunnan, Tongbiguan. (**A**) Male, holotype, dorsal view; (**B**) same, ventral view; (**C**) female, paratype, dorsal view; (**D**) head and pronotum of male, holotype, dorsal view; (**E**) prosternal process, male, holotype, lateral view; (**F**) same, female, paratype, lateral view; (**G**) *P. rugosicollis*, male, holotype and its labels. (**G**) photographed by Keita Matsumoto, copyright BMNH. Scale bars 5 mm for (**A**–**C**,**G**); 1 mm for (**D**–**F**).

**Redescription.** Small- to medium-sized, measuring 8.3–11.3 mm in length. Sexual dimorphism weakly expressed or absent. Coloration polymorphic, ranging from uniform to bicolored patterns, typically spanning yellow, brown to black (Figure 1A–F). Body robust (Figure 3A) to moderately elongate (Figure 5A,B), with glossy integument. Dorsal surfaces densely covered with long, erect pubescence, while ventral surfaces bear semi-erect pubescence of similar density.

Head smooth, nearly flat to slightly convex. Frons medially with shallow impression, apically overhanging clypeus base; supra-antennal carinae smooth and distinct laterally but gradually obsolete medially, not forming sharp frontal carina. Labrum transverse, subovate, finely punctate with dense setal cluster at distomedial margin. Mandibles bidentate (Figure 2G). Maxillary palpi with enlarged hatchet-like apical palpomere (Figure 2H). Eyes prominent, globose. Antennae elongate, extending to/about elytral mid-length in both sexes; scape robust; pedicel shortest; antennomeres III–XI elongate filiform (Figure 2K).

Pronotum subtrapezoidal, widest at apices of posterior angles; surface smooth, typically slightly convex medially (Figure 2A). Width between posterior angles exceeding length along midline. Anterior angle protruding distinctly beyond middle of anterior margin, protruding portion tapered, forming elongate process (Figure 2B, upper arrow); base with deep pit (Figure 2B, lower arrow). Posterior angle bidentate or with bidentate tendency (Figure 2C, right arrow). Pronotum carinate near lateral and anterior margins, space between carina and margin usually elevated. Anterior carina fine, space narrow; lateral carina gradually widening toward posterior angle, terminating at notch between bidentate teeth. Posterior margin medially with large M-shaped notch accommodating anterior portion of scutellar shield (Figure 2D, arrow). One or two additional small notches present near posterior margin of posterior angle (Figure 2C, left arrow, showing two notches); between these notches and transverse M-shaped notch, one faint median micro-notch present.

Prosternum subquadrate, sides weakly narrowing basally; chin-piece broadly convex, not surpassing apices of pronotal anterior angles (Figure 2F). Pronotosternal suture double-lined; prosternal process coarsely sculptured, ventrally expanded between procoxae then tapering apically, margins carinate, medially carinate; dorsally widened; apex typically notched (Figure 2E). Hypomeron punctation denser than pronotum. Tibia apically with two short spurs (Figure 2J, left arrows). Tarsomeres 3–4 lobed, lobe of tarsomere 4 larger than that of tarsomere 3 (Figure 2J, right four arrows). Metacoxal plate medially enlarged, strongly reduced laterally (Figure 2L). Elytra elongate (Figure 2N–P), base strongly expanded, humeri angulate; basal angles bidentate, accommodating pronotal posterior angles (Figure 2O,P, right arrows). Epipleuron well-developed. Margin of elytral suture near scutellar shield serrate (Figure 2P, left arrow). Apical portion narrowed, apices blunt, slightly pointed to spinous. Surface smooth, striae formed by punctate lines; striae 7–9 with larger punctures at basal half. Abdominal ventrite V typically rounded apically (Figure 2M), occasionally strongly narrowed into spinous projection (Figure 9D).

**Figure 4:**
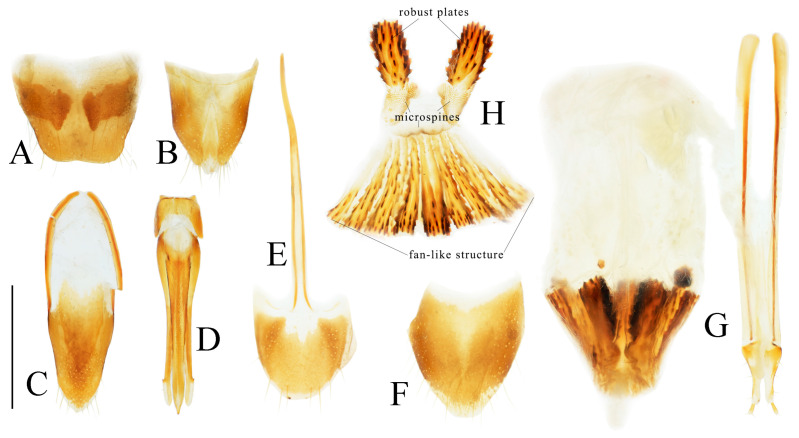
Pregenital segments and genitalia of *Parapenia fuxi* **sp. nov.**, male, holotype (**A**–**D**) and female, paratype (**E**–**H**). (**A**) Sternite VIII and tergite VIII, ventral view; (**B**) tergites IX–X, dorsal view; (**C**) sternite IX, dorsal view; (**D**) aedeagus, ventral view; (**E**) sternite VIII, ventral view; (**F**) tergite VIII, dorsal view; (**G**) ovipositor and genital tract, ventral view; (**H**) sclerotized plates of bursa copulatrix. Scale bar 1 mm.

Male: tergite VIII subtrapezoidal, apical margin medially concave (Figure 4A and Figure 6A) or typically convex (Figure 19A and Figure 24E). Sternite VIII bearing paired large sclerotized lateral areas, rest membranous (Figure 4A). Tergite IX anterior margin deeply emarginate medially, lobes rounded apically; tergite X slightly surpassing apices of tergite IX (Figure 4B). Sternite IX elongate, apical half usually tapering (Figure 4C). Aedeagus: uniform in shape across most species (Figure 4D, Figure 6D, Figure 8D, Figure 11G, Figure 13D, Figure 16H–Q, Figure 19G–M, Figure 23D,I and Figure 24H), except in *P. wulingshanensis* Schimmel, 2006 (Figure 22H–M). Median lobe and parameres elongate. Median lobe straight, exceeding beyond the apices of parameres, gradually narrowing distally. Paramere straight or slightly curved, with small subapical hook; post-hook portion elongate; apices generally blunt; lateral margin bearing sparse setae. Phallobase narrow, typically U-shaped or occasionally trapezoidal; in *P. wulingshanensis* V-shaped, strongly protruded posteriorly.

Female: tergite VIII apically narrowed (Figure 4F). Sternite VIII semioval; spiculum ventrale elongate, length equaling or up to 3 times of sternite VIII length (Figure 4E). Ovipositor elongate; coxite apically tapered; styli distinct, cylindrical, inserted subapically (Figure 4G). Bursa copulatrix large, apical portion bearing umbrella-like folded cluster of spinous sclerotized plates (Figure 4G). Cluster consisting of two robust plates and one fan-like structure (formed by six to ten elongate plates, usually median 2–3 plates longer than the rest plates) (Figure 4H, Figure 6H, Figure 8H, Figure 9I, Figure 11K, Figure 13H, Figure 16G, Figure 19F, Figure 20H and Figure 22G). Plate apices membranous, densely covered with microspines; microspination particularly dense on apical portions of robust plates.

**Bionomics.** Members of this genus predominantly inhabit densely vegetated montane forests, occurring at elevations ranging from 475 to 2700 m. Adults are primarily diurnal and can be collected through branch-beating of trees and shrubs along forest trails (Figure 1A,C,E). As noted by Suzuki [1], these beetles show a particular affinity for sheltering among dead leaves of evergreen trees at heights of 1–3 m above ground level. Nocturnal activity has also been observed, with specimens found crawling on foliage or ground surfaces (Figure 1D,F). Some species (e.g., *Parapenia villosa*, *P. wulingshanensis*, and *P. tonkinensis*, etc.) exhibit phototaxis and are attracted to artificial light sources (Figure 1B). Notably, adults have been documented feeding on dead insects during light trapping events (Figure 1B), suggesting carnivory in this genus. Individuals have also been collected while visiting flowers (Gan-Yan Yang, pers. observ.), suggesting possible nectar feeding.

**Distribution.** China, India, Laos, Myanmar, Thailand, and Vietnam (Figure 38 and Figure 39).

**Remarks.** This genus represents a well-defined taxon within the tribe Dimini. The pronotal morphology provides key diagnostic features: (1) elongate spinous anterior angles, each base with a distinct pit (Figure 25A); (2) dorsally bidentate posterior angles (or showing bidentate tendency, corresponding to Suzuki’s [1] “emarginate” condition) (Figure 25B); and (3) 1–2 small notches along posterior margin near posterior angles. The elytra exhibit complementary bidentate anterior angles that articulate precisely with the pronotal posterior angles (Figure 25B). Internal diagnostic characters include: (1) uniform aedeagal structure across species (Figure 4D, Figure 6D, Figure 8D, Figure 11G, Figure 13D, Figure 16H–Q, Figure 19G–M, Figure 23D,I and Figure 24H); and (2) consistent configuration of the sclerites of bursa copulatrix (Figure 4H, Figure 6H, Figure 8H, Figure 9I, Figure 11K, Figure 13H, Figure 16G, Figure 19F, Figure 20H and Figure 22G). These genitalic features provide additional reliable diagnostic criteria for generic identification. *Parapenioides* gen. nov. and *Sinopenia* gen. nov. are the only two genera sharing with *Parapenia* the bidentate posterior angles of pronotum and complementary bidentate anterior angles of elytra, However, *Parapenia* can be readily distinguished from both by the structure of the anterior angles of the pronotum (Figure 25A,D,G), as well as by the morphology of the aedeagus (Figure 4D, Figure 27D and Figure 29D) and the sclerotized plates of the bursa copulatrix (Figure 4G,H, Figure 27H and Figure 29H). For details see Figure 25 and Table 1.

Provisional key to known species of *Parapenia* from China *

**Figure 5:**
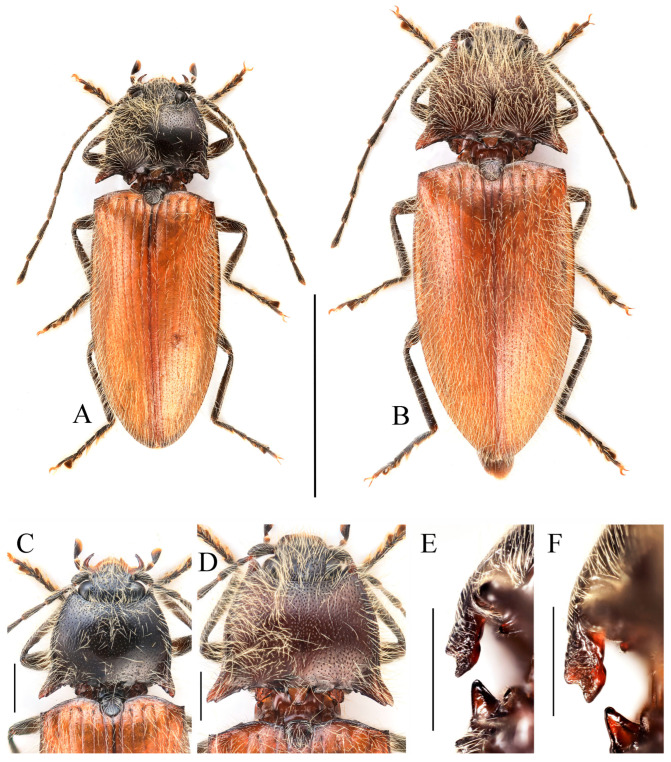
*Parapenia nyuwa* **sp. nov.** from Yunnan, Ailaoshan Mountain. (**A**) Male, holotype, dorsal view; (**B**) female, paratype, dorsal view; (**C**) head and pronotum of male, holotype, dorsal view; (**D**) head and pronotum of female, paratype, dorsal view; (**E**) prosternal process, male, holotype, lateral view; (**F**) same, female, paratype, lateral view. Scale bars 5 mm for (**A**,**B**); 1 mm for (****C**–F**).

1. Abdominal ventrite V with apex normally rounded (Figure 2M); tibiae and femora yellow to dark brown, unicolorous or diffusely bicolored (Figure 7B, Figure 10D, Figure 20B, Figure 21D and Figure 24B)………………………………………………………………………………………………‥ 2
- Abdominal ventrite V with apex strongly narrowed into a spinous projection (Figure 9D); tibiae and femora yellow, sharply contrasted with black at joint (Figure 9B)……………………………………………………………………………. *P. ruihangi* sp. nov.
2. Elytral apex with spinous projection (Figure 7A and Figure 24A)…………………………‥ 3
- Elytral apex blunt (Figure 3A and Figure 5A) or weakly pointed (Figure 10E and Figure 12C). 4
3. Pronotum uniformly black to brown; elytra yellow with small apical black spot (Figure 7A)…………………………………………………………………………………………………… *P. pangu* sp. nov.
- Pronotum bicolored, reddish-brown with black margins; elytra reddish-brown with black lateral margins (Figure 24A)………………………………‥………… *P. zhengi* sp. nov.
4. Pronotum with deep and coarse longitudinal wrinkles at sides of disc (Figure 20D); elytra and abdomen each sharply bicolored (Figure 20A,B)……………. *P. wuchaoi* sp. nov.
- Pronotum smooth, without wrinkles (Figure 12E) or with only indistinct weak traces (Figure 21H); elytra and abdomen each bicolored (Figure 3A,B), or unicolored (Figure 14A,B)……………………………………………………………………………………………. 5
5. Head and pronotum dark brown to black; elytra contrastingly yellow (Figure 5A and Figure 10A)…………………………………………………………………………………………. 6
- Head, pronotum, and elytra concolorous, or head and pronotum slightly darker; yellowish brown to dark brown overall (Figure 14G–N)………………………………………. 8
6. Body elongate, elytra/pronotum length ratio = 3.8–3.9:1; elytra predominantly yellow with indistinctly darkened margins (Figure 5A)…………………………… *P. nyuwa* sp. nov.
- Body stout, elytra/pronotum length ratio = 3.3–3.5:1; elytra unicolorous yellow or yellow with black apices…………………………………………………………………………. 7
7. Elytra totally yellow, apices slightly pointed (Figure 10E)…‥ *P. sausai* Schimmel, 1998
- Elytra yellow with apical 1/4 black, apices rounded (Figure 3A)……… *P. fuxi* sp. nov.
8. Body rather elongate (Figure 23A), elytra/pronotum length ratio = 4.0–4.1:1 (W China: C Yunnan)……………………………………………………‥…… *P. yunnana* Schimmel, 1993
- Body stout to moderate elongate, elytra/pronotum length ratio = 2.9–3.7:1………. 9
9. Aedeagus with phallobase enlarged, V-shaped, strongly protruding posteriorly (S & E China: Anhui, Fujian, Guangdong, Guangxi, Hunan, Jiangxi, Zhejiang) (Figure 22H–M)………………………………………………………………………………. *P. wulingshanensis* Schimmel, 2006
- Aedeagus with typical phallobase of the genus, truncate or rounded posteriorly (Figure 19G–M)………………………………………………………………………………………………………………‥‥. 10
10. Body robust, typical specimens with elytra/pronotum length ratio = 2.9–3.2:1; reddish-brown with pale elytral margins (N Vietnam; China: W & N Guangxi, S Guizhou, Hainan)……………………………………………………………………………………. *P. villosa* (Fleutiaux, 1936)
- Body slenderer, elytra/pronotum length ratio = 3.3–3.7:1; brown to dark brown without paler elytral margins……………………………………………………………………….11
11. Posterior angle of pronotum usually more narrowly produced; elytral apices usually pointed (China: Taiwan)……………………………………………‥‥. *P. taiwana* (Miwa, 1930)
- Posterior angle of pronotum usually more broadly produced; elytral apices usually blunt (N Vietnam; W China: Yunnan, Sichuan)…………… *P. tonkinensis* (Fleutiaux, 1918)

* Due to the unclear morphological boundaries among some species and the unresolved relationships between them (e.g., *P. taiwana* versus *P. tonkinensis*), the diagnostic characters selected for these taxa are not strongly distinctive. To aid identification in these cases, we have supplemented the key with distributional information. For this reason, the key is designated as provisional.

***Parapenia fuxi* sp. nov.**
ZooBank LSID: urn:lsid:zoobank.org:act:D575E9E1-30D7-49EE-8879-F457DEE6ED2A
Figure 3A–F, Figure 4A–H and Figure 38

**Chinese common name.** 伏羲长须叩甲

**Type locality.** China: Yunnan: Dehong Dai and Jingpo Autonomous Prefecture, Yingjiang County, Tongbiguan Township, 1000 m.

**Type material. Holotype: CHINA: Yunnan Province:** male (MYTC), Tongbiguan Township [铜壁关乡], Yingjiang County [盈江县], Dehong Dai and Jingpo Autonomous Prefecture [德宏傣族景颇族自治州], 1000 m, X.2017, no collector information. **Paratype:** 1 female (MYTC), same data as holotype.

**Diagnosis and comparison.** Bicolored species characterized by dark head (including antennae) and thorax (including scutellar shield); contrasting with reddish-yellow elytra and abdomen that darken apically (Figure 3A–C). Legs brown. Pronotum with stout anterior angle and bearing single small notch on posterior margin near base of each posterior angle (Figure 3D). Elytra/pronotum length ratio = 3.4–3.5:1; elytral apex blunt. Abdominal ventrite V apically rounded. Scutellar shield broadest at its distal half, with length equal to its maximum width. 

This new species bears a striking resemblance to *Parapenia rugosicollis* Schimmel, 2001 from India in overall coloration, but detailed examination of the *P. rugosicollis* holotype (Figure 3G) reveals several distinct morphological differences (in males): (1) pronotal anterior angles stout (versus narrowly pointed in *P. rugosicollis*); (2) posterior margin with a single small notch near each posterior angle base (versus two notches in *P. rugosicollis*); (3) antennae extending to elytral mid-length (versus not reaching elytral mid-length in *P. rugosicollis*); (4) antennomere II shorter, with antennomere II/III ratio = 1:1.5 (versus 1:1.3 in *P. rugosicollis*); (5) scutellar shield dark, widest distally, length/width ratio = 1:1 (versus brown, widest basally, length/width ratio = 1.3:1 in *P. rugosicollis*); (6) elytra more elongate, length/width ratio = 1.8:1 (versus 1.6:1 in *P. rugosicollis*); (7) elytral apices conjointly rounded (versus each separately pointed and angular in *P. rugosicollis*).

**Description.** Holotype, male: body length 10.2 mm, width 3.9 mm, antenna length 6.4 mm, pronotum length × width = 2.0 × 3.8 mm, elytra length 6.9 mm.

Body stout (elytra/pronotum length ratio = 3.5:1), bicolored, covered with moderately long pubescence. Head (including antennae and maxillary palpi), pronotum, scutellar shield, prosternum, and hypomeron blackish-brown; mandibles, meso- and metaventrite reddish-brown (metaventrite darker medially); abdomen yellowish-red with lateral portions of each segment dark brown, ventrite IV with dark brown apical margin, ventrite V entirely dark brown; elytra reddish-yellow with apical fifth dark brown; legs uniformly brown (Figure 3A,B). Pubescence yellow. 

Head smooth, nearly flat, bearing large shallow V-shaped depression; frons with nearly truncate anterior margin. Frons punctation coarse, interpuncture spaces less than one puncture diameter. Labrum rugose, with small punctures concentrated distally. Antennae extending to elytral mid-length; antennomere II/III ratio = 1:1.5 (Figure 3D).

Pronotum subtrapezoidal. Anterior angles short, stout, triangularly produced with rounded apices; lateral margins medially arcuate; posterior angles distinctly bidentate, outer tooth with rounded lateral margin and acute apex, inner tooth rounded, forming quadrate notch between teeth; single notch near posterior margin of each posterior angle. Punctures with flat and smooth intervals approximately 1–3 puncture diameters (Figure 3D).

Chin-piece with large umbilicate punctures, intervals mostly less than one puncture diameter; median portion of prosternum smooth, with transverse wrinkles and smaller, shallower punctures (intervals less than one puncture diameter). Prosternal process densely punctate ventrally, bearing large apicoventral notch in lateral view (shape as in Figure 3E). Hypomeron punctation grading sparser and smaller laterally, intervals less than one puncture diameter medially and about 1–2 puncture diameters laterally. Metaventrite with small punctures, intervals about 2–3 puncture diameters. Abdomen with punctures similar-sized but denser than metaventrite, intervals about 1–2 puncture diameters; ventrite V apically rounded. Scutellar shield widest distally, length/width ratio = 1:1, apex rounded. Elytra length/width ratio = 1.8:1; interstriae with sparse small punctures; apices rounded-subtruncate.

Abdominal tergite VIII subtrapezoidal with rounded apical angles, medially slightly concave (Figure 4A). Sternite VIII with dark portions exhibiting inner distal protrusions (Figure 4A). Tergite IX sides subparallel basally, abruptly curved medially, then linearly converging apically (Figure 4B). Sternite IX elongate, 3.0 times longer than wide (Figure 4C).

Aedeagus (Figure 4D): median lobe rugose, constricted medially, slightly expanded preapically, tapering to blunt apex. Paramere laterally concave medially; portion beyond subapical hook 3.5 times longer than wide, apex bluntly rounded. Phallobase with protruding basal angles.

Female paratype: similar to holotype male in general habitus, but with more robust body (Figure 3C). Body length 10.8 mm. Prosternal process shape slightly different, with apicoventral notch deeper and narrower (Figure 3F). Abdominal tergite VIII less sclerotized distally with rounded apex (Figure 4F); sternite VIII semioval, spiculum ventrale 3 times of sternite length (Figure 4E). Bursa copulatrix large with genus-typical sclerotized plates (Figure 4G,H).

**Distribution.** China: W Yunnan (Figure 38).

**Bionomics.** Unknown.

**Etymology.** This new species is named after Fuxi [伏羲], a male legendary figure in Chinese mythology who is regarded as one of the Three Sovereigns and the originator of human civilization, credited with teaching humanity skills such as fishing, hunting, and writing.

**Figure 6:**
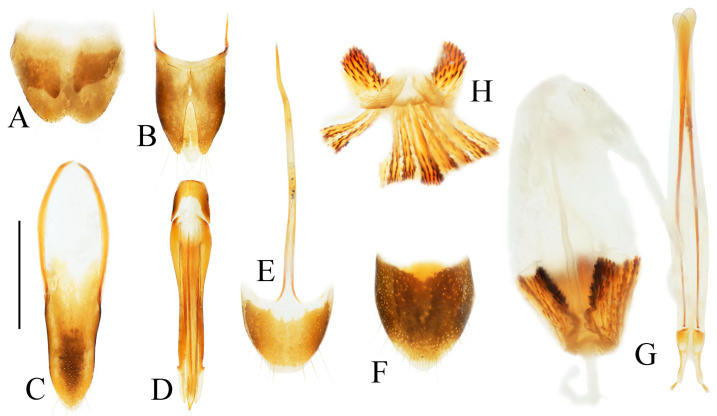
Pregenital segments and genitalia of *Parapenia nyuwa* **sp. nov.**, male, holotype (**A**–**D**) and female, paratype (**E**–**H**). (**A**) Sternite VIII and tergite VIII, ventral view; (**B**) tergites IX–X, dorsal view; (**C**) sternite IX, dorsal view; (**D**) aedeagus, ventral view; (**E**) sternite VIII, ventral view; (**F**) tergite VIII, dorsal view; (**G**) ovipositor and genital tract, ventral view; (**H**) sclerotized part of bursa copulatrix. Scale bar 1 mm.

***Parapenia nyuwa* sp. nov.**
ZooBank LSID: urn:lsid:zoobank.org:act:55B7559B-F875-4136-A769-795001380427
Figure 1E, Figure 5A–F, Figure 6A–H and Figure 38

**Chinese common name.** 女娲长须叩甲

**Type locality.** China: Yunnan: Pu’er City, Zhenyuan County, West slope of Ailaoshan Mountain, East of Nazhuang Village, Nazhuang Road, 1990–2050 m.

**Type material. Holotype: CHINA: Yunnan Province:** male (MYTC), Nazhuang Road [那壮路], East of Nazhuang Village [那壮村], West slope of Ailaoshan Mountain [哀牢山西坡], Zhenyuan County [镇沅县], Pu’er City [普洱市], 1990–2050 m, 4.VIII.2024, Lu Qiu leg. **Paratypes:** 2 specimens: 1 female (MYTC), same data as holotype; 1 female (MYTC), same data as holotype, but 5.VIII.2024.

**Diagnosis and comparison.** Species with elongate, bicolored body (Figure 5A,B). Head, antennae, pronotum, scutellar shield and entire ventral surface black to blackish brown; pronotal posterior angles light-colored; elytra yellow. Head flat. Pronotum with two small notches at posterior margin near base of each posterior angle. Antennae nearly reaching elytral mid-length. Elytra/pronotum length ratio = 3.8–3.9:1; elytral apices separately rounded. Abdominal ventrite V apically rounded. Scutellar shield longer than wide, widest basally.

This species resembles *P. yunnana* by the elongate elytra (Figure 23A,B,E,F) (both with elytral length/width ratio = 2.0:1), but can be distinguished from the latter by (1) the blackish brown to black body (versus brown to reddish brown in *P. yunnana*); (2) orange elytra with blackish margins (versus uniformly brownish yellow to brown in *P. yunnana*); (3) elytra/pronotum length ratio = 3.8–3.9:1 (versus 4.0–4.1 in *P. yunnana*).

**Description.** Holotype, male: body length 9.3 mm, width 3.3 mm, antenna length 6.0 mm, pronotum length × width = 1.7 × 2.9 mm, elytra length 6.6 mm.

Body elongate (elytra/pronotum length ratio = 3.9:1), bicolored, densely covered with long pubescence. Head (including antennae, labrum, and maxillary palpi), pronotum, scutellar shield, and entire ventral surface (hypomeron, prosternum, meso- and metaventrite, abdomen) black; mandibles reddish-brown; posterior angles of pronotum slightly reddish-brown; elytra orange, narrowly with blackish margins, apices shortly blackish. Pubescence whitish-yellow (Figure 5A).

Head smooth, with a large flattened median area. Frons anteriorly protruded and abruptly declivous medially; punctation umbilicate, interpuncture spaces flat, about 1–2 puncture diameters. Labrum with small punctures mostly confined to distal half. Antennae reaching slightly before elytral mid-length; antennomere II/III ratio = 1:1.3 (Figure 5C).

Pronotum curvilinearly subtrapezoidal (Figure 5C). Anterior angles stout, curved outward, apices rounded; lateral margins arcuate medially; posterior angles distinctly bidentate, outer tooth sharply narrowed apically, inner tooth rounded, forming an obtuse-angled notch; two notches present near posterior angles, outer one larger. Punctures with smooth flat interspaces, mostly 2–3 puncture diameters apart.

Chin-piece with dense umbilicate punctures, interspaces less than one puncture diameter. Prosternum medially smooth, with smaller punctures and intervals equal about 2–3 puncture diameters. Prosternal process ventrally densely punctate around broad median carina; apical notch large (Figure 5E). Hypomeron punctures dense and oval medially, intervals less than one puncture diameter, gradually sparser laterally. Metaventrite with fine punctures, intervals equal 2–3 puncture diameters. Abdominal punctures denser and slightly larger than on metaventrite, intervals about 1–2 puncture diameters; ventrite V apically rounded. Elytra elongate, sides subparallel; elytral length/width ratio = 2.0:1. Interstriae slightly elevated, smooth but somewhat coarse, with sparse small punctures; apices separately rounded, without spines.

Abdominal tergite VIII subtrapezoidal, apical margin with two rounded lobes, medially deeply concave (Figure 6A). Sternite VIII with two dark portions, each bearing a small distal inner protrusion (Figure 6A). Tergite IX with sides subparallel basally, abruptly curved medially, then narrowing straight to apex (Figure 6B). Sternite IX elongate, 3.6 times longer than wide (Figure 6C).

Aedeagus (Figure 6D): median lobe narrowing from base to apex, more abruptly constricted preapically, apex with small blunt protrusion. Paramere with basal portion rugose, sides convex, abruptly narrowed from basal third, then tapering straight to elongate apical portion; portion beyond subapical hook 4 times longer than wide, apex sharply pointed. Phallobase with blunt basal lateral angles.

Female paratypes: body length 10.4–11.3 mm. Similar to holotype male in general habitus, but larger in size (Figure 5B); pronotum brown (Figure 5D); prosternal process with shorter apical protrusion (Figure 5F). Tergite VIII semioval, sides converging, apex blunt (Figure 6F); sternite VIII semioval, spiculum ventrale slender, approximately 3.5 times length of sternite VIII (Figure 6E). Bursa copulatrix large, with genus-typical spinose sclerotized plates (Figure 6G,H).

**Distribution.** China: Yunnan (Figure 38).

**Bionomics.** This species inhabits broad-leaved forests and was collected by sweeping branches and foliage along roadsides during daytime (Figure 1E).

**Etymology.** The species is named after Nyuwa (also spelled Nüwa or Nvwa) [女娲], the goddess in Chinese mythology who created humanity and repaired the sky after a great catastrophe.

**Figure 7:**
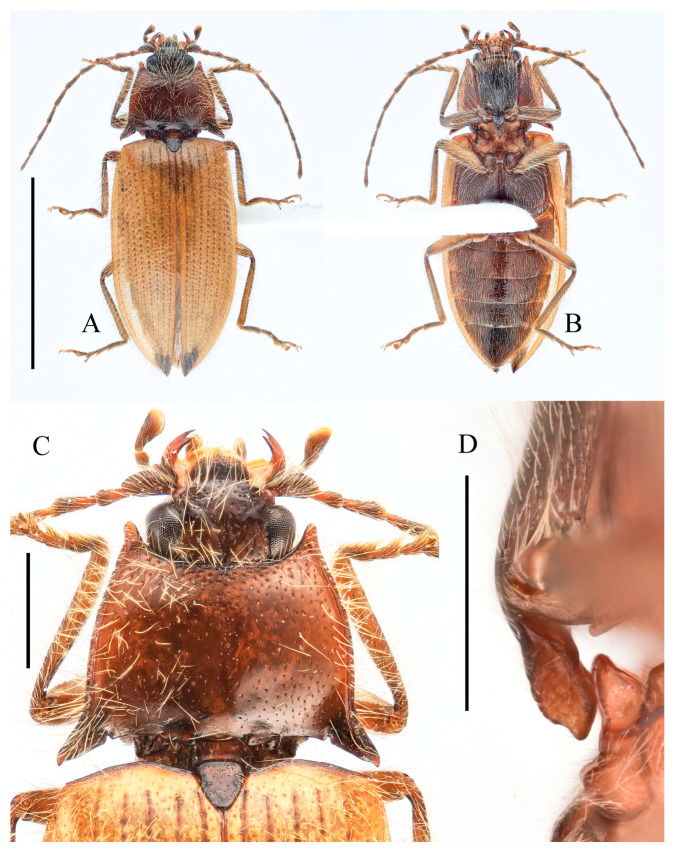
*Parapenia pangu* **sp. nov.**, male, holotype from Yunnan, Bingzhongluo. (**A**) Habitus, dorsal view; (**B**) same, ventral view; (**C**) head and pronotum, dorsal view; (**D**) prosternal process, lateral view. Scale bars 5 mm for (**A**,**B**); 1 mm for (**C**,**D**).

**Figure 8:**
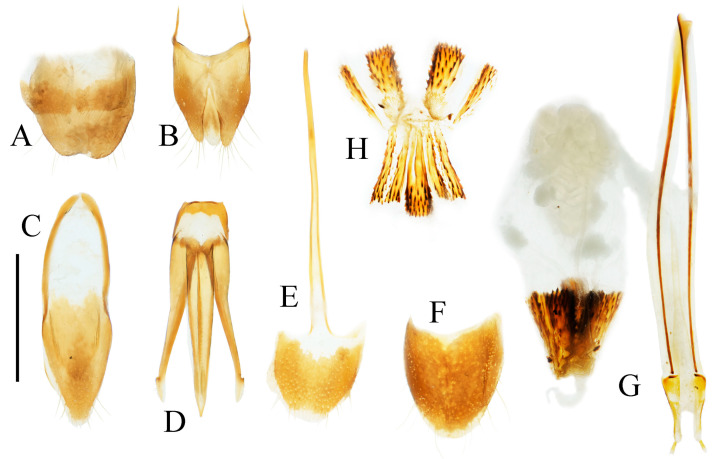
Pregenital segments and genitalia of *Parapenia pangu* **sp. nov.**, male, holotype (**A**–**D**) from Yunnan, Gongshan, Bingzhongluo and female, paratype (**E**–**H**) from Yunnan, Weixi. (**A**) Sternite VIII and tergite VIII, ventral view; (**B**) tergites IX–X, dorsal view; (**C**) sternite IX, dorsal view; (**D**) aedeagus, ventral view; (**E**) sternite VIII, ventral view; (**F**) tergite VIII, dorsal view; (**G**) ovipositor and genital tract, ventral view; (**H**) sclerotized part of bursa copulatrix. Scale bar 1 mm.

***Parapenia pangu* sp. nov.**
ZooBank LSID: urn:lsid:zoobank.org:act:DDE99EF3-37EA-4C04-B1E1-BC918C81225B
Figure 1C, Figure 7A–D, Figure 8A–H and Figure 38

**Chinese common name.** 盘古长须叩甲

**Type locality.** China: Yunnan: Nujiang Prefecture, Gongshan County, South of Bingzhongluo Town, 2115 m.

**Type material. Holotype: CHINA: Yunnan Province:** male (MYTC), South of Bingzhongluo Town [丙中洛镇], Gongshan County [贡山县], Nujiang Lisu Autonomous Prefecture [怒江傈僳族自治州], 2115 m, 14.VII.2021, Lu Qiu leg. **Paratypes:** 6 specimens: 1 male (SZPU, ex MYTC), same data as holotype; 1 male (MYTC), Lusaihe Village [鲁腮河村], Luzhang Town [鲁掌镇], Lushui County [泸水县], Nujiang Lisu Autonomous Prefecture, 2280 m, 3.VIII.2022, Yan-Dong Chen leg.; 1 male and 1 female (MYTC), Shiyueliang Township [石月亮乡], Fugong County [福贡县], Nujiang Lisu Autonomous Prefecture, 2400–2700 m, VI.2022, local leg.; 1 male and 1 female (MYTC), Ahualuohe River [阿花洛河], Zhonglu Township [中路乡], Weixi County [维西县], Diqing Zang Autonomous Prefecture [迪庆藏族自治州], 2400–2700 m, VI.2024, local leg.

**Diagnosis and comparison.** Body bicolored, generally brown; legs dull yellow-brown; elytra yellow with black apices (Figure 7A,B). Pronotum with one small notch near the base of posterior margin of each posterior angle (Figure 7C). Elytra/pronotum length ratio = 4.1:1; elytral apex spinous. Abdominal ventrite V apically rounded. Scutellar shield widest at base.

**Figure 9:**
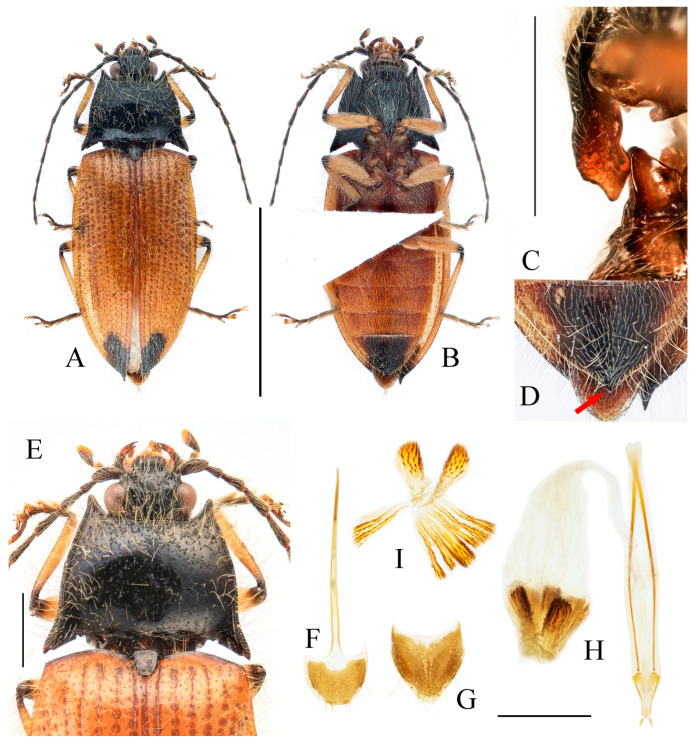
*Parapenia ruihangi* sp. nov., female, holotype from Yunnan, Mengku. (**A**) Habitus, dorsal view; (**B**) same, ventral view; (**C**) prosternal process, lateral view; (**D**) abdominal ventrite V (with red arrow indicating the spinous projection); (**E**) head and pronotum, dorsal view; (**F**) sternite VIII, ventral view; (**G**) tergite VIII, dorsal view; (**H**) ovipositor and genital tract, ventral view; (**I**) sclerotized part of bursa copulatrix. Scale bars 5 mm for (**A**,**B**); 1 mm for (**C**,**E**–**I**); (**D**) not to scale.

This new species resembles *Parapenia ruihangi* sp. nov. in general appearance, but differs in coloration and the shape of the abdominal ventrite V. In *P. pangu* sp. nov., the head, pronotum, and ventral side are uniformly brown; legs are dull yellow-brown (Figure 7A,B). In contrast, *P. ruihangi* sp. nov. has a black head and pronotum, with a bicolored ventral side (prothorax and abdominal ventrite V black, remaining parts yellowish brown), and yellow legs with black femoral–tibial joints. The abdominal ventrite V is gradually narrowed toward apex, with apical portion rounded in *P. pangu* sp. nov., but spinous in *P. ruihangi* sp. nov. (Figure 9D).

**Description.** Holotype, male: body length 9.1 mm; width 3.4 mm; antenna length 5.4 mm; pronotum length × width = 1.5 × 2.8 mm; elytra length 6.2 mm.

Body narrowed (elytra/pronotum length ratio = 4.1:1), bicolored, flat, narrow, covered with moderately long pubescence. Head (including antennae, mandibles, and maxillary palpi), pronotum, scutellar shield, prosternum, metaventrite, and abdomen brown; hypomeron brown with lateral portions gradually yellowish. Elytra and mesonotum yellow; elytral apices with triangular black spot; basal margins black. Legs brown, with some parts dull yellow. Pubescence yellow (Figure 7A,B).

Head nearly flat, smooth, with broad, shallow V-shaped depression medially. Anterior margin of frons nearly truncate. Punctures on frons dense medially (intervals less than one puncture diameter), sparser laterally (more than one puncture diameter). Labrum rugose, punctures mostly aggregated in distal half. Antennae short, not reaching elytral mid-length; antennomere II/III ratio = 1:1.4 (Figure 7C).

Pronotum subtrapezoidal (Figure 7C); anterior angles protruding outward with rounded apices. Lateral margins nearly straight, slightly arched medially. Posterior angles indistinctly bidentate; outer tooth laterally rounded, apically sharp; inner tooth reduced, forming shallow notch with outer tooth. One small notch present near base of each posterior angle on posterior margin. Puncture intervals flat, smooth, 3–6 puncture diameters.

Chin-piece with small punctures, intervals about one puncture diameter. Prosternum smooth, sparsely punctate; intervals 3–5 puncture diameters. Prosternal process with distinct median carina, ventral apex with shallow notch (Figure 7D). Hypomeron with dense punctures mesally (intervals less than one puncture diameter), becoming sparser laterally (intervals about 2 puncture diameters). Metaventrite with sparse punctures, intervals about 3 puncture diameters. Abdomen similar in puncture density and size; apex of abdominal ventrite V rounded. Scutellar shield widest at base, as long as wide, basal margin straight; apical portion narrowed, apex emarginate. Elytra elongate, elytral length/width ratio = 1.8:1; interstriae sparsely punctate. Elytral apices distinctly spinous.

Tergite VIII subtrapezoidal, apical margin concave medially (Figure 8A). Sternite VIII with two transverse dark patches; distal margin lacking protrusions (Figure 8A). Tergite IX sides straight and subparallel in basal half, abruptly curved near middle; distal half narrowed, sides straight (Figure 8B). Sternite IX elongate, 3.2 times longer than wide (Figure 8C).

Aedeagus (Figure 8D): median lobe narrowed from base to apex, apical portion more abruptly narrowed; apex tapered, forming small blunt protrusion. Paramere with rough basal portion; lateral sides subparallel, bent outward at basal third, then tapered straightly toward apex; apical portion slightly curved inward, portion beyond subapical hook 2.9 times longer than wide; apex tapered. Phallobase with small basal protrusions.

Paratypes: body length 8.3–9.4 mm. Sexual dimorphism slight; males and females generally similar to holotype. Some individuals with narrower or smaller pronotum; some with darker pronotum, scutellar shield, and ventral surface. Size of dark apical spot on elytron variable.

Female paratypes with tergite VIII less sclerotized apically, apex rounded (Figure 8F); sternite VIII semioval; spiculum ventrale 3.5 times longer than sternite VIII (Figure 8E). Bursa copulatrix with genus-typical sclerotized plates (Figure 8G,H).

**Distribution.** China: Yunnan (Figure 38).

**Bionomics.** The material from Bingzhongluo was collected by beating branches and shrubs along a mountain road (Figure 1C).

**Etymology.** This new species is named after Pangu [盘古], the giant in Chinese mythology who created the world by separating heaven and earth.

***Parapenia ruihangi* sp. nov.**
ZooBank LSID: urn:lsid:zoobank.org:act:D4859E56-A8D8-4DBB-ACAF-41F7552A6811
Figure 9A–I and Figure 38

**Chinese common name.** 瑞航长须叩甲

**Type locality.** China: Yunnan: Lincang City, Shuangjiang County, Mengku Town, 1800 m.

**Type material. Holotype: CHINA: Yunnan Province:** female (MYTC), Mengku Town [勐库镇], Shuangjiang County [双江县], Lincang City [临沧市], 1800 m, 13.VII.2019, Rui-Hang Dong leg.; **Paratype:** 1 female (MYTC), Southeast of Xima Town [昔马镇], Yingjiang County, Dehong Dai and Jingpo Autonomous Prefecture, 1693 m, 28.VII.2019, Quan-Yu Ji leg.

**Diagnosis and comparison.** A highly distinctive species with striking bicolored pattern: mesoventrite, metaventrite, elytra, legs, and abdomen bright yellow; head, antennae, prothorax, abdominal ventrite V, femoro-tibial joints, and elytral apices black (Figure 9A,B). Head with V-shaped depression. Pronotum with single notch near base of each posterior angle on posterior margin. Elytral/pronotum length ratio = 3.8:1, each apically spinous. Abdominal ventrite V spinous apically (Figure 9D). Scutellar shield widest at base. Comparison see under *Parapenia pangu* sp. nov.

**Description.** Holotype, female: body length 8.7 mm; width 3.3 mm; antenna length 5.2 mm; pronotum length × width = 1.6 × 2.8 mm; elytra length 6.0 mm.

Body elongate (elytra/pronotum length ratio = 3.8:1), bicolored, narrow, covered with moderately long pubescence. Head, pronotum, scutellar shield, prosternum, and hypomeron black; labrum and antennae dark brown; mandibles and maxillary palpi brown; prosternal process, mesonotum, metaventrite, and abdomen yellowish brown; ventrite V blackish brown with lateral portions yellowish brown. Legs yellow, with joints between femora and tibiae black; tarsi and claws dark brown. Elytra orange, with basal margin dark and apices bearing a black heart-shaped spot. Pubescence yellow (Figure 9A,B).

Head smooth, almost flat, with a large, shallow V-shaped depression. Anterior margin of frons protruding medially in a blunt triangle. Intervals between punctures on frons approximately 1–2 puncture diameters. Labrum with small punctures mostly distributed along the distal margin. Antennae reaching slightly before the middle of elytra; antennomere II/III ratio = 1:1.5 (Figure 9E).

Pronotum subtrapezoidal (Figure 9E). Anterior angles slender, protruding straight and tapered, apices rounded. Lateral margins nearly straight, very slightly arched at mid-length. Posterior angles indistinctly bidentate: outer tooth with rounded lateral margin and sharp apex; inner tooth reduced, forming a shallow notch with the outer tooth. One notch present near base of each posterior angle on posterior margin. Puncture intervals on pronotum flat and smooth, approximately 3–5 puncture diameters.

Chin-piece with small umbilicate punctures, intervals between punctures approximately 1–2 puncture diameters. Middle of prosternum smooth, weakly wrinkled, with smaller punctures than on chin-piece, intervals approximately 2 puncture diameters. Prosternal process with distinct median carina, shallowly notched ventro-apically; shape as in Figure 9C. Hypomeron with oval punctures, intervals subequal to or less than one puncture diameter. Metaventrite sparsely punctate, intervals approximately 2 puncture diameters. Abdominal punctures similar in size and spacing to those on metaventrite. Apex of ventrite V spinous (Figure 9D). Scutellar shield widest at base, slightly longer than wide at its broadest point; basal margin convex medially; apical portion narrowed, apex rounded. Elytral length/width ratio = 1.8:1. Interstriae sparsely punctate. Elytral apices with long, sharp spines (Figure 9A).

Tergite VIII semi-oval, lateral margins of distal half narrowing straightly toward apex, apex rounded (Figure 9G). Sternite VIII semi-oval; spiculum ventrale slender, 3.6 times longer than sternite VIII length (Figure 9F). Ovipositor slender. Bursa copulatrix with genus-typical spinous sclerotized plates (Figure 9H,I).

Female paratype: body length 8.8 mm. Apical black spot on elytra smaller and slightly different in shape from holotype; elytral apical spines slightly incurved. Otherwise similar to the holotype.

**Distribution.** China: Yunnan (Figure 38).

**Bionomics.** The specimen from Xima was collected by sweep-netting vegetation along a roadside.

**Etymology.** The species is named in honor of Mr. Rui-Hang Dong [董瑞航], the collector of the holotype.

**Figure 10:**
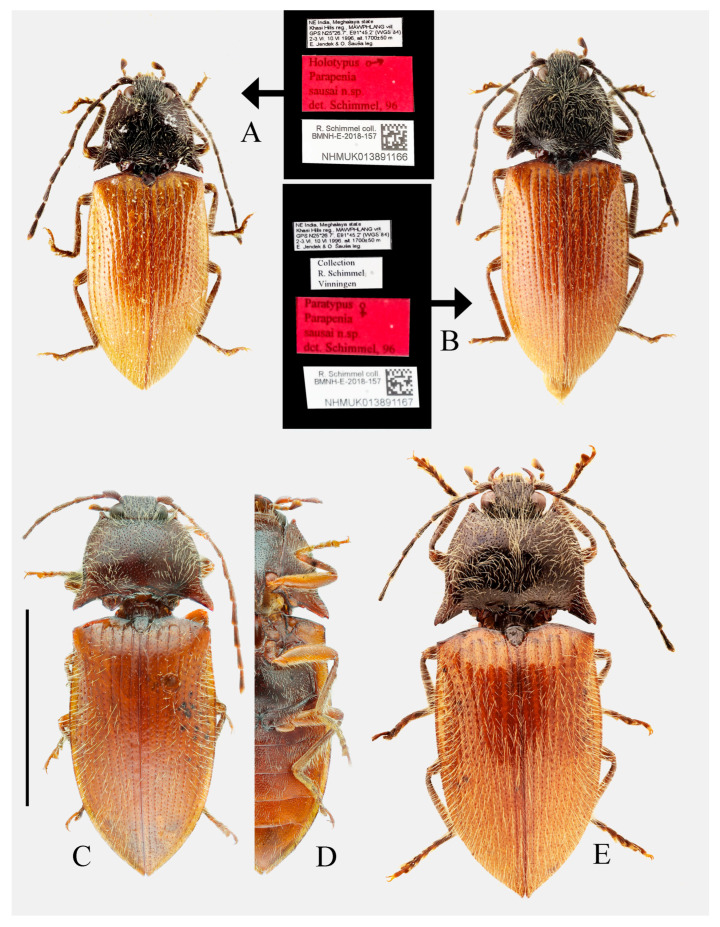
*Parapenia sausai.* (**A**) Male, holotype and its labels; (**B**) female, paratype and its labels; (**C**) male from Xizang, Zayü, dorsal view; (**D**) same, ventral view; (**E**) female from Xizang, Mêdog, dorsal view. (**A**,**B**) photographed by Keita Matsumoto, copyright BMNH. Scale bar 5 mm.

**Figure 11:**
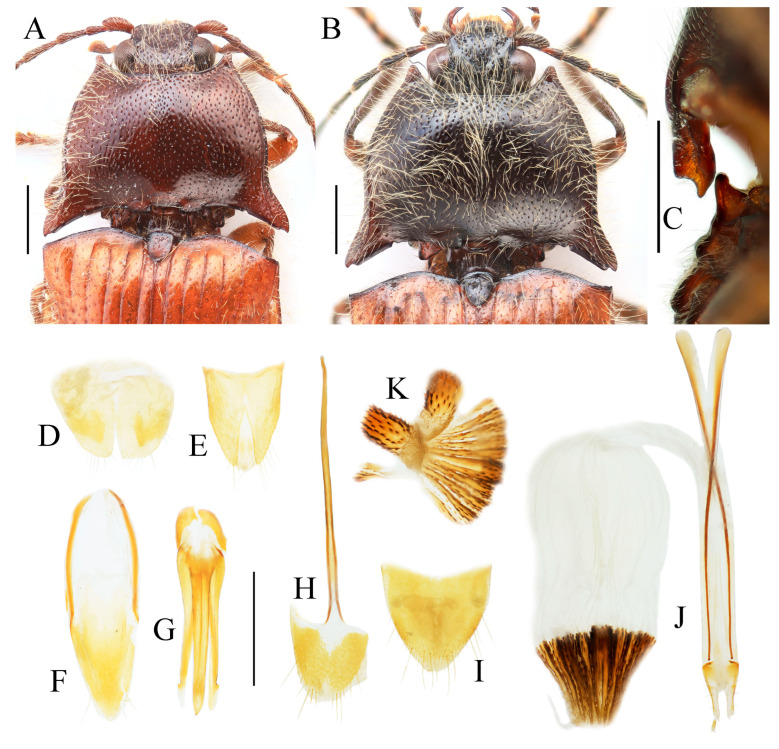
Characters of *Parapenia sausai*, male from Xizang, Zayü (**A**,**C**–**G**) and female from Xizang, Mêdog (**B**,**H**–**K**). (**A**) Head and pronotum, dorsal view; (**B**) same; (**C**) prosternal process, lateral view; (**D**) sternite VIII and tergite VIII, ventral view; (**E**) tergites IX–X, dorsal view; (**F**) sternite IX, dorsal view; (**G**) aedeagus, ventral view; (**H**) sternite VIII, ventral view; (**I**) tergite VIII, dorsal view; (**J**) ovipositor and genital tract, ventral view; (**K**) sclerotized part of bursa copulatrix. Scale bars 1 mm.

***Parapenia sausai* Schimmel, 1998**
Figure 10A–E, Figure 11A–K and Figure 38

*Parapenia sausai* Schimmel, 1998: 152 [9] (original description); Kundrata et al. 2018: 33 [3] (catalogue).

**Chinese common name.** 绍氏长须叩甲

**Type locality.** NE India: Meghalaya, Khasi Hills, Mawphlang, 1700 m.

**Type material examined. Holotype** of *Parapenia sausai* Schimmel, 1998: male (NHMUK), “Holotypus [print] ♂/*Parapenia* [print]/*sausai* n. sp. [print]/det. Schimmel, 96 [print]”, “NE India, Meghalaya state/Khasi Hills reg., MAWPHLANG vill./GPS N25°26.7′, E91°45.2′ (WGS 84)/2–3.VI. 10.VI 1996, alt. 1700 ± 50 m/E. Jendek & O. Šauša leg.” [print], “R. Schimmel coll./BMNH-E-2018-157/NHMUK013891166” [print, with a QR code]. **Paratype** of *Parapenia sausai* Schimmel, 1998: 1 female (NHMUK), “Paratypus [print] ♀/*Parapenia* [print]/*sausai* n.sp. [print]/det. Schimmel, 96 [print]”, “NE India, Meghalaya state/Khasi Hills reg., MAWPHLANG vill./GPS N25°26.7′, E91°45.2′ (WGS 84)/2–3.VI. 10 VI 1996, alt. 1700 ± 50 m/E. Jendek & O. S Šauša leg.” [print], “Collection/R. Schimmel/Vinningen” [print], “R. Schimmel coll./BMNH-E-2018-157/NHMUK013891167” [print, with a QR code].

**New material examined. CHINA: Xizang Autonomous Region:** 2 males (HBUM), Lower Zayü Town [下察隅镇], Zayü County [察隅县], Nyingchi City [林芝市], 8.VIII.2002, Ming-Sheng Zhu et al. leg.; 1 female (MYTC), Gelin Village [格林村], Mêdog County [墨脱县], Nyingchi City, 1500 m, 22.VIII. 2020, Yu-Chen Zheng leg.

**Diagnosis.** Body length 9.8–10.7 mm, width 3.8–4.2 mm, antenna length 5.8–6.0 mm, pronotum length × width = 2.0–2.1 × 3.6–3.8 mm, elytra length 6.7–6.9 mm (measurements are based on the Xizang specimens only). Body robust (elytra/pronotum length ratio = 3.3–3.4:1), bicolor (Figure 10A–E), head (including antennae, mandibles and maxillary palpi), pronotum, scutellar shield, prosternum, hypomeron, meso- and metaventrite reddish brown to black. Elytra and abdomen orange. Legs yellowish brown to brown. Pubescence yellow. Head with V-shaped depression. Antennae reaching slightly before middle of elytra. Pronotum subtrapezoidal (Figure 11A,B). Anterior angle of pronotum protruding forward or slightly outward, apex rounded. Lateral margins of pronotum arched. Posterior angle of pronotum bidentate, outer tooth with lateral margin rounded, apex sharp. One small notch present near posterior angle. Intervals between punctures of pronotum approximate 2–4 puncture diameters. Prosternal process laterally shaped as Figure 11C. Elytral length/width ratio = 1.6–1.8:1; apices each slightly spinous independently. Ventrite V triangular, apex narrowly blunt.

Male: tergite VIII semioval (Figure 11D). Sternite VIII with two darkened areas; inner margins of distal end weakly protruded (Figure 11D). Sides of tergite IX straight and subparallel in basal half, abruptly bent near middle; distal half straight, narrowed toward apices (Figure 11E). Sternite IX elongate, 3.3 times longer than wide (Figure 11F). Aedeagus typical of the genus (Figure 11G).

Female: abdominal tergite VIII subtriangular, apex blunt (Figure 11I). Sternite VIII semi-oval; spiculum ventrale slender, approximately 3.3 times longer than sternite VIII length (Figure 11H). Bursa copulatrix large, with genus-typical sclerotized plates (Figure 11J,K).

**Distribution.** China [**new record**]: Xizang; India (Figure 38).

**Bionomics.** Unknown.

**Remarks.** The examined male and female specimens were collected from two geographically close localities in Xizang (Zayü and Mêdog counties). The two males exhibit identical external morphology but differ slightly from the female, primarily in body coloration outside the elytra, legs and abdomen (Figure 10C–E and Figure 11A,B): males are reddish-brown, while the female is black. Additionally, the males’ pronotum anterior angles are slightly outward, whereas the female’s are directed forward. Despite these differences, all three specimens share key diagnostic characters of *P. sausai*, including a stout body, orange elytra with dark pronotum and head, slightly spinous elytral apices, and a posterior pronotum margin bearing a single notch near the posterior angle (Figure 10A,B). Despite their overall consistency with *P. sausai*, the Xizang specimens show slight morphological differences, including a larger body size (9.8–10.7 mm vs. 8.1–8.6 mm of India specimens) and more convex pronotal margins. Due to the currently limited specimens, these differences can only be considered as intraspecific variation. However, questions remain unresolved because, unlike the Indian population, which shows no sexual color dimorphism, the Xizang specimens potentially exhibit such variation. Additional specimens, especially from intermediate regions, are needed to confirm their conspecificity.

**Figure 12:**
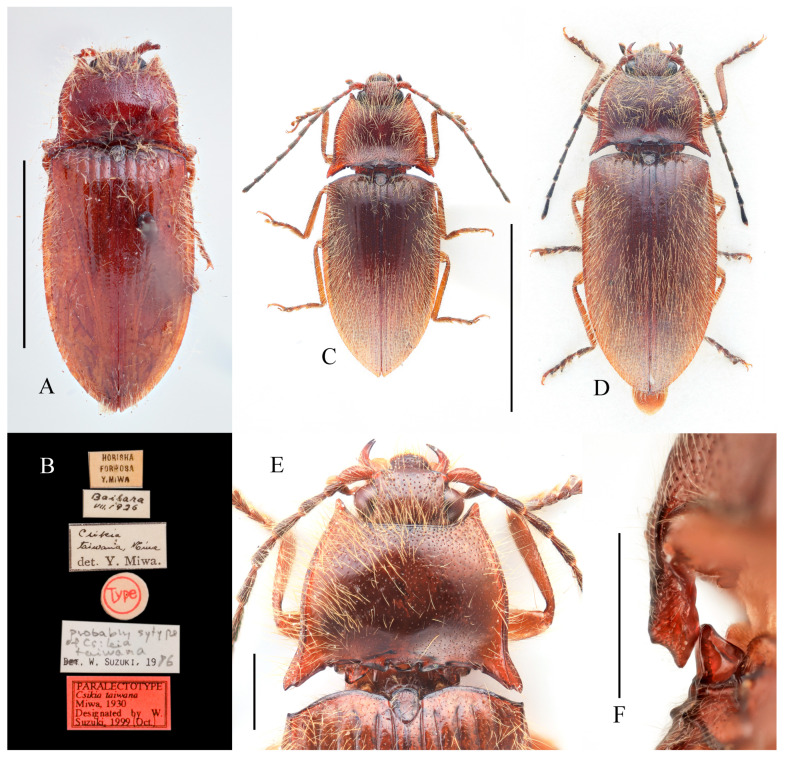
*Parapenia taiwana.* (**A**,**B**) Sex unknown, paralectotype and its labels; (**C**) male from Dahanshan, dorsal view; (**D**) female from Alishan, dorsal view; (**E**) head and pronotum, male from Taidong, dorsal view; (**F**) prosternal process, male from Dahanshan, lateral view. Scale bars 5 mm for (**A**,**C**,**D**); 1 mm for (**E**,**F**).

**Figure 13:**
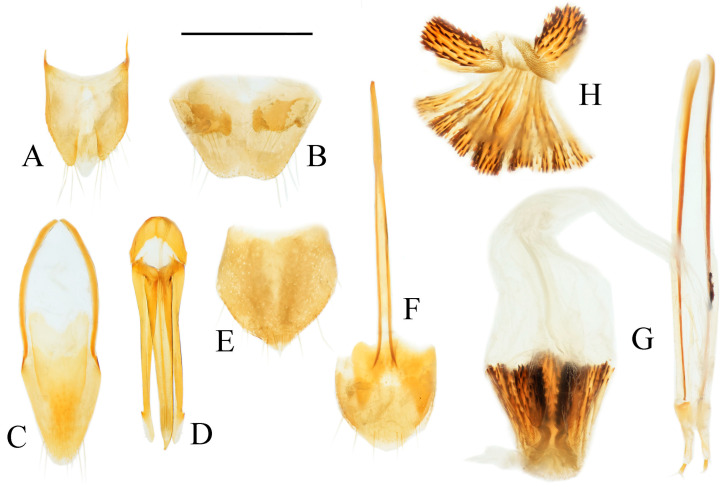
Pregenital segments and genitalia of *Parapenia taiwana*, male from Dahanshan (**A**–**D**) and female from Wufeng, Hsinchu (**E**–**H**). (**A**) Tergites IX–X, dorsal view; (**B**) sternite VIII and tergite VIII, ventral view; (**C**) sternite IX, dorsal view; (**D**) aedeagus, ventral view; (**E**) tergite VIII, dorsal view; (**F**) sternite VIII, ventral view; (**G**) ovipositor and genital tract, ventral view; (**H**) sclerotized part of bursa copulatrix. Scale bar 1 mm.

***Parapenia taiwana* (Miwa, 1930)**
Figure 12A–F, Figure 13A–H and Figure 39

*Csikia taiwana* Miwa, 1930: 93 [6], holotype from “Taihorinsho [=Dalin, Chiayi嘉义大林]”, paratypes from “Mt. Arisan [=Alishan嘉义阿里山]”, “Kwarenko [=Hualien花莲港]”, and “Baibara [Hsinseng, Renai, Nantou南投仁爱新生]” (original description); Miwa 1931: 141 [32] (catalogue); Miwa 1934: 228 [33] (checklist and key); Ôhira 1972: 7 [34], records from “Hassenzan [=Baxianshan, Heping, Taichung台中和平八仙山]” and “Bukai [=Fazhi, Ren’ai, Nantou南投仁爱法制]”; Bouwer 1991: 57 [35] (checklist).

*Parapenia taiwana*: Suzuki 1982: 89 [1] (new combination); Kishii 1991: 3 [36] (record from Kao-hsiung高雄); Jiang 1993: 146 [37] (checklist); Schimmel 1996: 158 [2] (diagnosis); Kishii 1997: 11 [38] (illustration); Suzuki 1999: 121 [28] (catalogue); Hua 2002: 86 [39] (catalogue); Cate 2007: 184 [29] (catalogue); Platia & Schimmel 2007: 58 [40] (records from Taitung台东, Kaoshiung高雄, Ilan宜兰); Kundrata et al. 2018: 33 [3] (catalogue); Jiang & Yang 2023: 45 [31] (catalogue).

**Chinese common name.** 宝岛长须叩甲

**Type locality.** China: Taiwan: “Taihorinsho” [Chiayi County, Dalin Township].

**Type material examined. Paralectotype** of *Csikia taiwana* Miwa, 1930: 1 individual, sex unknown (TARI), “Baibara [南投仁爱新生] VII.1926 [handwriting]”, “Horisha [埔里]/Formosa/Y. Miwa [print]”, “*Csikia* [handwriting]/*taiwana*, Miwa [handwriting]/det. Y. Miwa. [print]”, “Type [print in red with red circle]”, “Probably Syntype [handwriting]/of *Csikia* [handwriting]/*taiwana* [handwriting]/Det. Suzuki, 19 [print] 86 [handwriting]”, “PARALECTOTYPE/*Csikia taiwana*/Miwa, 1930/Designated by W./Suzuki, 1999 (Oct.)” [print].

**New material examined. CHINA: Taiwan Province:** 2 males and 1 female (MYTC), Xueba Farm [雪霸农场], Wufeng Township [五峰乡], Hsinchu County [新竹县], 1900 m, 12.IX.2017, Wen-I Chou leg.; 2 males (MYTC), Mt. Dahanshan [大汉山], Chunri Township [春日乡], Pingtung County [屏东县], 1380 m, 13.VI.2018, Wen-I Chou leg.; 1 female (MYTC), Erwanping [二万坪], Alishan Mountain [阿里山], Chiayi County [嘉义县], 2000 m, 13.IX.2017, Wen-I Chou leg.; 1 male and 1 female (MYTC), Liyuan [栗园], Haiduan Township [海端乡], Taitung County [台东县], 1850 m, 27.IX.2017, Wen-I Chou leg.; 2 female (HNHM), “TAIWAN, Ilan country [misspelling of “county”],/Fu-Shan Botanical/garden, 700 m, at light [台湾宜兰县福山植物园]”, “25–27.IX.2000, leg./L.Papp, L. Peregovites/& L. Ronkay”, “PARAPENIA/taiwana (Miwa)/det. Platia 2006”; 1 male (PCRK), “TAIWAN, Kaohsiung/county, Shanping Forest/Recreation Area,/near Liukuei [台湾高雄市六龟区扇平森林游乐区]”, “22°58′16″ N, 120°41′15″ E,/at light, 19–21.IX.2002,/L. Ronkay & O. Merkl”, “PARAPENIA/taiwana (Miwa)/det. Platia 2006”; 1 female (PCRK), “TAIWAN, Hualien co./Rei Suei, [台湾花莲县瑞穗乡]/logging road 1500 m”, “23°21′09 N, 121°16′49 E,/31.VIII.2003,/G. Csorba & Z. Korsós”; 4 ex. (NHMUK), “TAIWAN, Nantou County/N24°02.530′; E121°12.555′/beating, etc., 1920 m/6.viii.2008, M.V.L. Barclay,/H. Mendel & R. Ewers/BMNH(E) 2008-85”; 2 ex. (NHMUK), “TAIWAN, Kaohsiung County/Jongjhihguan [高雄桃园玉山中之关], 131 km post/N 23°17.229 E 120°53.777/12.viii.2008, 1934 m, H. Mendel/M.V.L. Barclay & L.C. Shih/BMNH(E) 2008-85”; 1 female (NHMUK), “TAIWAN, I-Lan/Hsien, Yuenshan/village, Fushan/Botanical Garden”, “23–24 July 2005/A C Galsworthy”, “BMNH {E}/2005-165/A G Galsworthy”; 1 sex unknown (TARI), “C. Taiwan: Yu-shih/1750 m, Nantou Hsien [台湾南投县鱼池乡]/4.VIII.1981/T. Lin & W. S. Tang”, “*Parapenia*/*taiwana*/(Miwa, 1930)/Det. W. Suzuki”.

**Diagnosis.** Body length 9.0–10.7 mm. Body usually stout (elytra/pronotum length ratio = 3.3–3.4:1). Coloration reddish brown to dark brown, with legs paler and antennae and tarsi blackish (Figure 12A,C,D). Antennae reaching before middle of elytra. Head and pronotum with small and sparse punctures; intervals between punctures approximately 3–5 puncture diameters. Pronotum (Figure 12E) with one to two notches near posterior angle. Metaventrite with much smaller and shallower punctures than pronotum. Elytral length/width ratio = 1.7–1.8:1. Elytral apices each slightly pointed or blunt. Male: Tergite VIII subtrapezoidal, apical margin shallowly concave medially (Figure 13B). Aedeagus typical of the genus (Figure 13D). Bursa copulatrix large, with genus-typical spinous sclerotized plates (Figure 13G,H).

**Distribution.** China: Taiwan (Figure 39).

**Bionomics.** This species inhabits broad-leaved forests and can be caught by sweeping net through roadside branches and leaves. It is attracted to light at night.

**Remarks.** This species closely resembles *P. tonkinensis*, with only subtle differences between the two. In *P. taiwana*, the posterior angle of pronotum appears less divergent and more sharply pointed in the outer tooth, and the elytral apices are slightly pointed (sometimes not obvious). Although we provisionally treat them as distinct species, these diagnostic differences may represent intraspecific variation, particularly considering their dull coloration and the highly similar male genitalia and sclerotized plates of the bursa. Clarification of their taxonomic status requires further investigation, such as molecular analyses.

**Figure 14:**
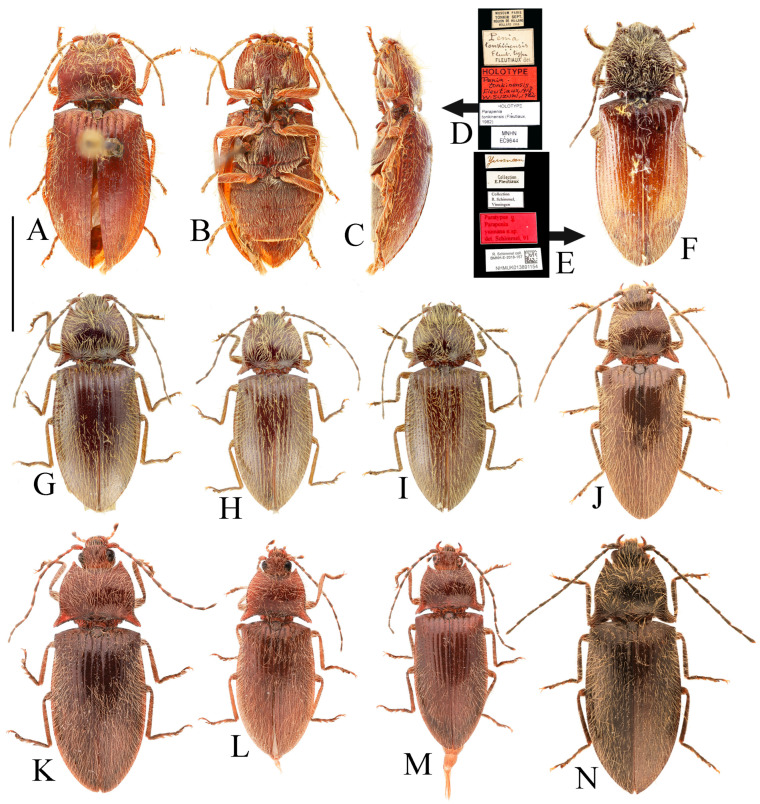
*Parapenia tonkinensis.* (**A**–**D**) Holotype of *Penia tonkinensis* Fleutiaux, 1918, female: (**A**) dorsal view; (**B**) ventral view; (**C**) lateral view; (**D**) labels; (**E**,**F**) paratype of *P. yunnana Schimmel*, 1993, female: (**E**) labels; (**F**) dorsal view; (**G**–**I**) individuals from Vietnam, Sa Pa: (**G**) female, dorsal view; (**H**,**I**) males, dorsal view; (**J**) male from Yunnan, Baihualing; (**K**–**M**) individuals from Sichuan, Dafengding: (**K**,**L**) females, dorsal view; (**M**) male, dorsal view; (**N**) female from Yunnan, Daweishan. (**A**–**D**) photographed by Christophe Rivier, copyright MNHN; (**E**,**F**) by Keita Matsumoto, copyright BMNH. Scale bar 5 mm.

**Figure 15:**
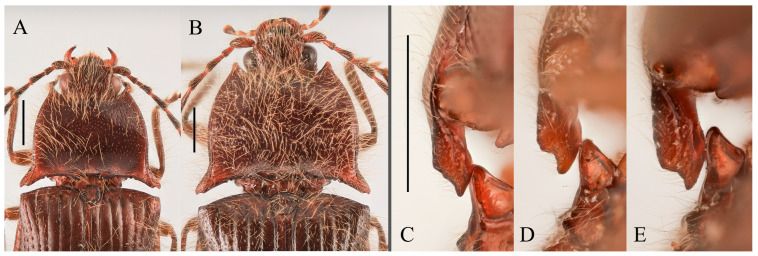
Characters of *Parapenia tonkinensis* from Sichuan, Dafengding. (**A,B**) Head and pronotum, dorsal view; (**A**) male; (**B**) female; (**C**–**E**) variation in prosternal process, lateral view; (**C**,**D**) male; (**E**) female. Scale bars 1 mm.

**Figure 16:**
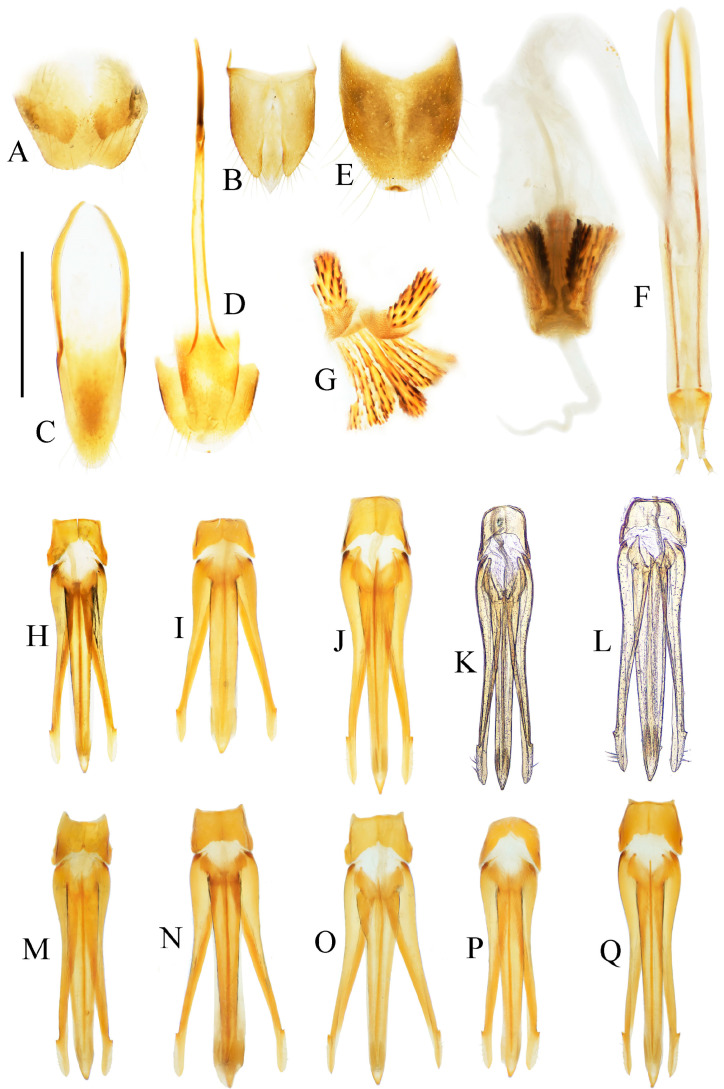
Pregenital segments and genitalia of *Parapenia tonkinensis*, male ((**A**–**C**) from Sichuan, Dafengding, (**H**–**Q**) from various localities) and female ((**D**–**G**) from Dafengding). (**A**) Sternite VIII and tergite VIII, ventral view; (**B**) tergites IX–X, dorsal view; (**C**) sternite IX, dorsal view; (**D**) sternite VIII, ventral view; (**E**) tergite VIII, dorsal view; (**F**) ovipositor and genital tract, ventral view; (**G**) sclerotized part of bursa copulatrix; (**H**–**Q**) aedeagi, ventral view: (**H**) Dafengding; (**I**–**J**) Yunnan, Daweishan; (**K,L**) Vietnam, Sa Pa; (**M**) Sichuan, Gengda; (**N**) Sichuan, Guangyuan, Tangjiahe; (**O**) Sichuan, Emeishan; (**P**) Sichuan, Yibin, Laojunshan; (**Q**) Yunnan, Baihualing. Scale bar 1 mm.

***Parapenia tonkinensis* (Fleutiaux, 1918)**
Figure 1F, Figure 14A–N, Figure 15A–E, Figure 16A–Q and Figure 39

*Penia tonkinensis* Fleutiaux, 1918: 226 [4] (original description); Schenkling 1927: 419 [41] (catalogue); Fleutiaux 1936: 291 [5] (record from “Chapa” [=Sa Pa]); Bouwer 1991: 237 [42] (catalogue).

*Parapenia tonkinensis*: Suzuki 1982: 92 [1] (new combination); Suzuki 1989: 212 [43] (new record from Mengtze [=Mengzi蒙自], Yunnan, China); Schimmel 1996: 159 [2] (diagnosis); Cate 2007: 184 [29] (catalogue); Kundrata et al. 2018: 34 [3] (catalogue); Jiang & Yang 2023: 45 [31] (catalogue).

*Pristilophus tonkinensis* Fleutiaux, 1924: 138 [44]; Schenkling 1927: 404 [41] (catalogue). Synonymized by Suzuki [43].

**Chinese common name.** 暗色长须叩甲

**Type locality.** North Vietnam: Cao Bằng: Hạ Lang.

**Type material examined. Holotype** of *Penia tonkinensis* Fleutiaux, 1918: female (MNHN), “HOLOTYPE [print]/*Penia* [handwriting]/*tonkinensis* [handwriting]/Fleutiaux, 1918 [handwriting]/W. SUZUKI, 1982 [handwriting]”, “*Penia* [handwriting]/*tonkinensis* [handwriting]/Fleut. Type [handwriting]/FLEUTIAUX det. [print]”, “MUSEUM PARIS/TONKIN SEPT./RÉGION DE HA-LANG/MOLLAED 1906” [handwriting], “HOLOTYPE/*Parapenia/tonkinensis* (Fleutiaux,/1982)” [misprint, should be “1918”], “MNHN/EC9644” [print]. **Paratype** of *Parapenia yunnana* Schimmel, 1993, 1 female (NHMUK), “Paratypus ♀/*Parapenia*/*yunnana* n. sp./det. Schimmel, 91” [print], “yunnan” [handwriting], “Collection/R. Schimmel/Vinningen” [print], “R. Schimmel coll./BMNH-E-2018-157/NHMUK013891154” [print, with a QR code].

**New material examined. CHINA: Sichuan Province:** 1 male (MYTC), Chenjiawuji [陈家屋脊], Laojunshan Mountain [老君山], Zhongdu Town [中都镇], Pingshan County [屏山县], Yibin City [宜宾市], 1300 m, 9.VII.2024, Yi-Shu Wang leg.; 2 males and 3 females (MYTC), Nieshijue [捏史觉], Dafengding N.R. [大风顶自然保护区], Yonghong Township [永红乡], Mabian County [马边县], Liangshan Prefecture [凉山州], 1458 m, 20–24.VIII.2023, Tian-Long He leg.; 1 ex. (MYTC), Wanniansi [万年寺], Mt. Emei [峨眉山], Leshan City [乐山市], 1020 m, 12.VIII.2024, Zhi-Yu Zhang leg.; 1 male (SCU), Mt. Emei, Leshan City, 4.VII.2010, Yun-Chun Li & Yong-Shen Pan; 1 ex (MYTC), E-Hong Road [峨洪路], Mt. Emei, Leshan City, 24.VII.2018, Lu Qiu & Jing-Fei Han leg.; 1 male (MYTC), Gengda Town [耿达镇], Wenchuan County [汶川县], Aba Zang and Qiang Autonomous Prefecture [阿坝藏族羌族自治州], 23.VII.2012, Yong Zhou leg.; 2 females (MYTC), near Jiucaiyuan [韭菜园], Guixi Town [桂溪镇], Jiangyou County-Level City [江油市], Mianyang City [绵阳市], 1560 m, 104°40′34″ E, 31°52′22″ N, 26.VIII.2024, Lu Qiu leg.; 1 male and 2 ex (MYTC), Maoxiangba [毛香坝], Tangjiahe N.R. [唐家河自然保护区], Qingchuan County [青川县], Guangyuan City [广元市], 6.IX.2021, Lu Qiu leg.; 1 ex. (MYTC), Caijiaba [蔡家坝], Tangjiahe N.R., Qingchuan County, Guangyuan City, 7.IX.2021, Cheng-Bin Wang leg.; 2 females (MYTC), Qingping Road [青平路], near Weiba Village[魏坝村], Qingxi Town [清溪镇], Qingchuan County, Guangyuan City, 1363 m, 32.4310 N, 104.7577 E, 25.VII.2024, Yong Zhou, Tian-Xuan Gu, Zi-Xu Yin leg.; 1 ex. (NHMW, ex. coll. P. Cate), “CHINA, SICHUAN Prov./70 km NW Chengdu, 1435 m/Qingcheng Hou Shan mts. [四川成都青城后山]/9.–14.VII.2004, S. Murzin leg.”; 1 male (HNHM), “25/8: CHINA, Sichuan, Ya’an, Yucheng Distr.,/Bifengxia Town, Houyan/Village [雅安市雨城区碧峰峡镇后盐村], hillside forest,”, “923 m, 30°06′39.7″ N/103°02′53.5″ E,/25.VII.2011, D. Rédei”; 1 male (NHMUK), “CHINA, Sichuan 12.-14.VII./Baoxing env. 1995/cca 50 km NNW of Yaan [雅安市宝兴县]/30″20 N 102″50′ E/M. Tryzna et O. Safranek lg”, “Colletion/R. Schimmel,/Vinningen”, “Parapenia/yunnana Schim./det. Schimmel, 96”, “R. Schimmel coll./BMNH-E-2018-157/NHMUK013891158 [with a QR code]”; **Yunnan Province:** 1 male (NHMUK), “CH, Yunnan 22.5-2.6.93 100 km W of Kunming, DIAOLIN NAT. RESERV. [=Diaolinshan N.R.雕翎山自然保护区, in Lufeng County禄丰县, Chuxiong Yi Autonomous Prefecture楚雄彝族自治州], E. Jendek, O. Sausa leg”, “Colletion/R. Schimmel,/Vinningen”, “Parapenia/yunnana Schim./det. Schimmel, 96”, “R. Schimmel coll./BMNH-E-2018-157/NHMUK013891155 [with a QR code]”; 2 females and 1 ex. (DLU), Mt. Daweishan [大围山], Pingbian County [屏边县], Honghe Hani and Yi Autonomous Prefecture [红河哈尼族彝族自治州]; 1500–2100 m, 15–17.VII.2018, Ji-Shan Xu leg.; 1 male and 2 females (MYTC), ibid, but 2050 m, 29.V–3.VI.2021, Hao Xu, Xin-Yuan Zhang & Xin-Ying Liu leg.; 1 male (MYTC), idid, 3–5.VIII.2021, Hao-Yi Liu leg.; 1 female (MYTC), Baihualing [百花岭], Mt. Gaoligongshan [高黎贡山], Baoshan City [保山市], 1400–1900 m, 20–23.VI.2020, Lu Qiu leg.; 1 male (MYTC), ibid, but 1500 m, 8.VI.2020, Yu-Chen Zheng & Jia-Zhi Zhang leg.; 1 female (MYTC), South of Bingzhongluo Town, Gongshan County, Nujiang Prefecture, 2115 m, 14.VII.2021, Lu Qiu leg.; **VIETNAM: Lào Cai Province:** 2 males, 5 females (ZISP), “Вьетнам, горы у Ша-Па, 1600–2000 м, 7.8.1963 г., Кабаков [Vietnam, mountains near Sa Pa, 1600–2000 m, 7.VIII.1963, O. N. Kabakov leg.]; 1 female (ZISP), N Vietnam, Lao Cai Prov., Sapa env., 1500–2000 m, 25.V.1963, O. N. Kabakov leg.; 1 male (DEMSU), N Vietnam, Lao Cai Prov., near Sa Pa Town, Xin Chai Vill., N 22°21′7.2″, E 103°48′12″; N 22°21′08″, E 103°47′59″, h = 1437–1464 m, 16.V.2018, P. V. Romantsov leg.; 1 female (DEMSU), N Vietnam, Lao Cai Prov., near Sa Pa Town, Chapi Homestay, N 22°19′52.3″, E 103°49′45.2″, h = 1285 m, at light, 10–21.V.2018, P. V. Romantsov leg.; 1 female (DEMSU), N Vietnam, Lao Cai Prov., near Sa Pa Town, Cat Cat Vill., N 22°19′35.3″, E 103°49′47.8″–N 22°19′12.2″, E 103°49′17.9″, h = 1280–1337 m, 21.V.2018, P. V. Romantsov leg.; 1 male (DEMSU), N Vietnam, Lao Cai Prov., near Sa Pa Town, Xin Chai Vill., N 22°21′7.2″, E 103°48′12″–N 22°21′08″, E 103°47′59″, h = 1437–1464 m, 16.V.2018, P. V. Romantsov leg.; 1 female (PCJHo), “VIETNAM N./SaPa-1550 m/25.V.–9.VI.91/Strnad Jan lgt.”.

**Diagnosis.** Body length 8.3–10.6 mm. Body stout (elytra/pronotum length ratio = 3.3–3.6:1), brown, reddish brown to dark brown in general (Figure 14). Antennae dark brown, with each antennomere reddish brown basally. Legs usually with slightly lighter color. Antennae reaching slightly before the middle of elytra. Pronotum subtrapezoidal (Figure 15A,B). Anterior angle of pronotum protruding forward, apex rounded. Lateral margins of pronotum slightly arched in the middle. Posterior angle of pronotum distinctly bidentate, outer tooth with lateral margin rounded, apex sharp, pointing laterally; inner tooth blunt, forming shallow notch with outer tooth. Two small notches present at posterior margin near posterior angle, inner one sometimes indistinct or absent. Intervals between punctures on pronotal disc equal approximately 3–6 puncture diameters. Prosternal process shape variable, usually with notch ventrally at apex (Figure 15C–E). Elytral length/width ratio = 1.6–1.8:1; apices blunt, sometimes each weakly pointed. Aedeagus typical for this genus, phallobase with basal margin straight, sometimes lateral corners protruded (Figure 16H–Q). Bursa copulatrix large, with genus-typical spinous sclerotized plates (Figure 16F,G).

**Distribution.** China: Yunnan, Sichuan [**new record**]; Vietnam: Cao Bằng, Lào Cai (Figure 39).

**Bionomics.** This species inhabits broad-leaved forests and can be caught by sweeping nets through roadside branches and leaves during daytime. The species is also active at night (Figure 1F) and shows attraction to light (Lu Qiu, personal observation).

**Remarks.** *P. tonkinensis* was originally described from northern Vietnam [4], and later recorded from China (Mengzi, Yunnan) by Suzuki [43]. Based on the examination of numerous specimens, the present study confirms that the species is widely distributed from northern Vietnam to northern Sichuan, China.

This species is morphologically close to *P. taiwana*, though their relationship remains unresolved (see remarks under *P. taiwana*). It also resembles *P. yunnana* in its dull coloration, but can be distinguished by the elytra/pronotum length ratio (3.3–3.7 in *P. tonkinensis* versus 4.0 in *P. yunnana* holotype). This is, so far, the most evident diagnostic difference between the two species. Based on the examination of the paratype (with an imprecise locality information of “Yunnan”) (Figure 14E,F) and additional specimens (two specimens, respectively, from Baoxing, Sichuan and Diaolinshan, Yunnan) of *P. yunnana* preserved in NHMUK, we found that the paratype designated by Schimmel, as well as the specimens he identified should be assigned to *P. tonkinensis*. These reassignments are supported by their similar elytra/pronotum length ratio (3.6 in paratype of *P. yunnana*, 3.7 in Yunnan specimen and 3.5 in Sichuan specimen).

The species exhibits considerable intraspecific variation, with notable differences observed even among individuals collected from the same locality. Not only does the shape of the apical portion of parameres vary greatly, but the length of both median lobe and parameres shows distinct differences, regardless of whether the specimens were collected from different localities or from the same site (e.g., Figure 16I,J from Daweishan, Yunnan; 16K–L from Sa Pa, Vietnam). The shape of the prosternal process also varies significantly (Figure 15C–E), rendering it unreliable as a diagnostic character for this species. Males sometimes differ from females mainly in body size: some males are noticeably smaller, with smaller pronotum (Figure 14M and Figure 15A). However, this is not consistent, as very small females have also been observed (Figure 14L).

In spite of the extensive morphological variation, some populations stand out and warrant further investigation. For instance, individuals from Daweishan Mountain, Yunnan, tend to have darker body coloration, with some specimens appearing nearly black and exhibiting more narrowed elytral apices (Figure 14N). Specimens from northwestern Yunnan (Gongshan and Baoshan) (Figure 14J show narrower pronotum (lateral margins less convex) with denser punctures than those from other regions in males, with puncture intervals of about 2–4 diameters, versus 3–6 diameters elsewhere (Figure 14J). These populations may represent cryptic species; however, given the currently limited material and the considerable degree of intraspecific variation, we provisionally treat all of them as *P. tonkinensis*.

**Figure 17:**
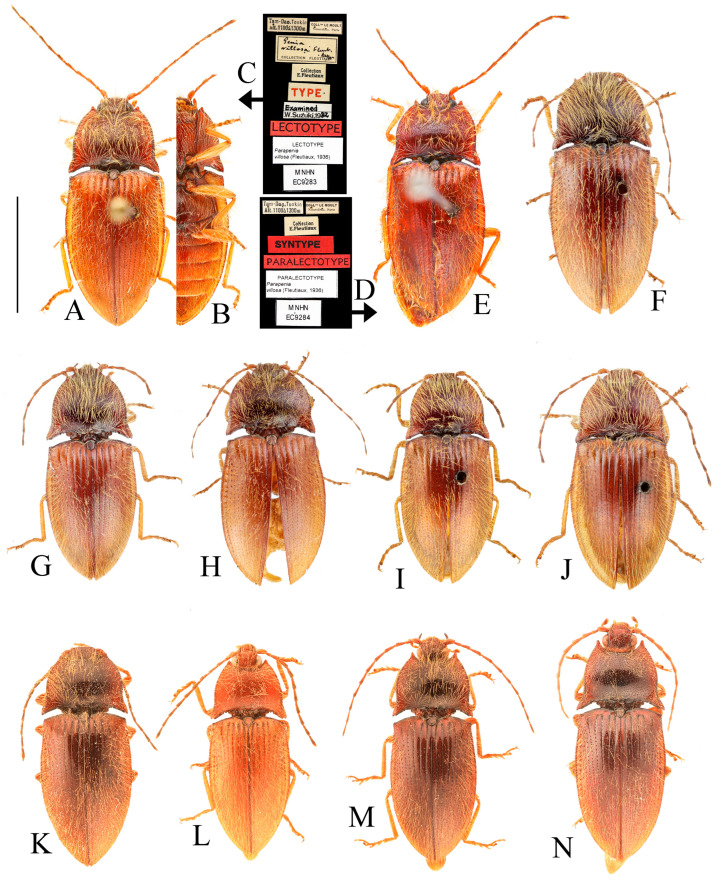
*Parapenia villosa.* (**A**–**C**) Lectotype of *Penia villosa* Fleutiaux, 1936, sex undetermined: (**A**) dorsal view; (**B**) ventral view; (**C**) labels; (**D**,**E**) paralectotype of *Penia villosa* Fleutiaux, 1936, sex undetermined: (**D**) labels; (**E**) dorsal view; (**F**–**J**) individuals from Vietnam, Tam Dao ((**F**,**J**) females; (**G**–**I**) males); (**K**) male from Hainan, Wuzhishan; (**L**) female from Hainan, Jiangfengling; (**M**) female from Guizhou, Maolan; (**N**) male from Guizhou, Maolan. (**A**–**E**) photographed by Christophe Rivier, copyright MNHN. Scale bar 5 mm.

**Figure 18:**
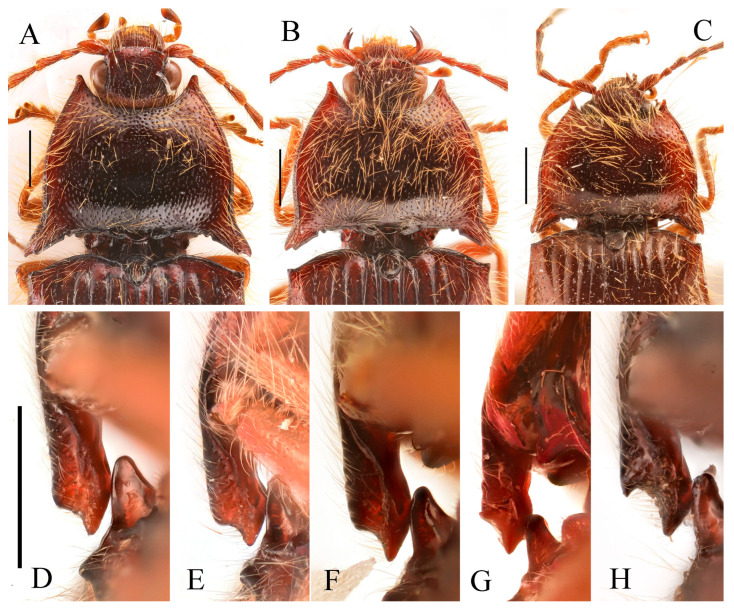
Characters of *Parapenia villosa*. (**A**–**C**) Head and pronotum, dorsal view: (**A**) male from Guizhou, Maolan; (**B**) female from the same locality as (**A**); (**C**) female from Hainan, Bawangling; (**D**–**H**) variation in prosternal process, lateral view: (**D**) male from Guizhou, Maolan, Shishangsenlin; (**E**) female from the same locality as (**D**); (**F**) female from Guizhou, Maolan, Duoyinshan; (**G**) female from Bawangling; (**H**) male from Hainan, Wuzhishan. Scale bars 1 mm.

**Figure 19:**
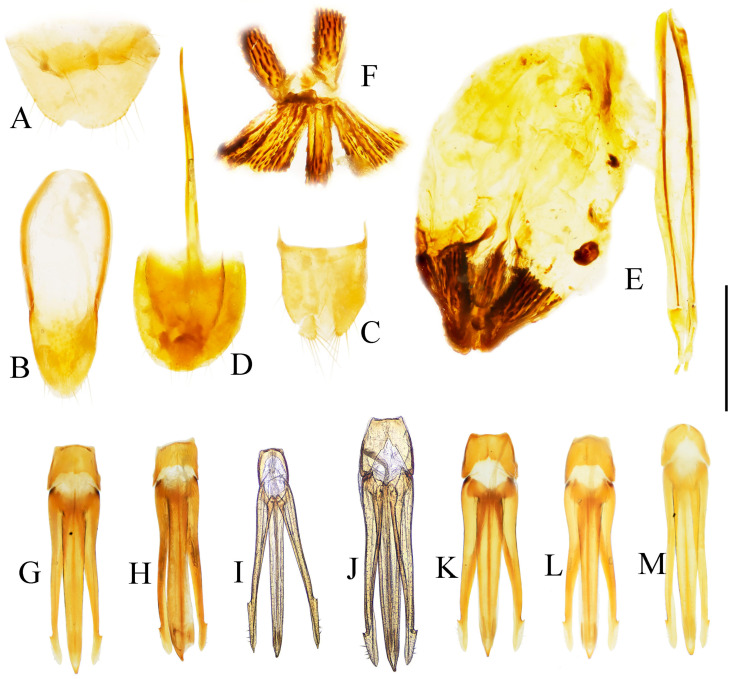
Pregenital segments and genitalia of *Parapenia villosa*, male ((**A**–**C**) from Hainan, Wuzhishan, (**G**–**M**) from various localities) and female ((**D**–**F**) from Hainan, Bawangling). (**A**) Sternite VIII and tergite VIII, ventral view; (**B**) sternite IX, dorsal view; (**C**) tergites IX–X, dorsal view; (**D**) sternite VIII and tergite VIII, dorsal view; (**E**) ovipositor and genital tract, ventral view; (**F**) sclerotized part of bursa copulatrix; (**G**–**M**) aedeagi, ventral view: (**G**) Hainan, Wuzhishan; (**H**) Hainan, Jiangfengling; (**I**,**J**) Vietnam, Tam Dao ((**I**) comes from specimen in Figure 17I; (**J**) from Figure 17G); (**K**) Guizhou, Maolan, Duoyinshan; (**L**) Guizhou, Maolan, Shishangsenlin; (**M**) Guangxi, Napo. Scale bar 1 mm.

***Parapenia villosa* (Fleutiaux, 1918)**
Figure 1A, Figure 17A–N, Figure 18A–H, Figure 19A–M and Figure 39

*Penia villosa* Fleutiaux, 1936: 290 [5] (original description); Bouwer 1991: 237 [42] (catalogue).

*Parapenia villosa*: Suzuki 1982: 93 [1] (new combination, lectotype designation); Schimmel 1996: 160 [2] (diagnosis); Kundrata et al. 2018: 34 [3] (catalogue).

**Chinese common name.** 多毛长须叩甲

**Type locality.** Vietnam: Vĩnh Phúc: Tam Dao.

**Type material examined. Lectotype** of *Penia villosa* Fleutiaux, 1936: sex undetermined (MNHN), “Tam-Dao. Tonkin/Alt. 1100á1300 m” [print], “COLL on LE MOULT/Naturaliste, Paris” [print], “*Penia* [handwriting]/*villosa* Fleut. [handwriting]/type [handwriting]/COLLECTION FLEUTIAUX [print]”, “Collection/E.Fleutiaux” [print], “TYPE” [print in red], “Examined [handwriting]/.W.Suzuki, 19 [print]82 [handwriting]”, “LECTOTYPE” [print in red background], “LECTOTYPE/*Parapenia*/*villosa* (Fleutiaux, 1936)” [print], “MNHN/EC9283” [print]. **Paralectotype** of *Penia villosa* Fleutiaux, 1936: sex undetermined (probably female) (MNHN), “Tam-Dao. Tonkin/Alt. 1100á1300 m” [print], “COLL on LE MOULT/Naturaliste, Paris” [print], “*Penia* [handwriting]/*villosa* Fleut. [handwriting]/type [handwriting]/COLLECTION FLEUTIAUX [print]”, “Collection/E.Fleutiaux” [print], “SYNTYPE” [print in red background], “PARALECTOTYPE” [print in red background], “PARALECTOTYPE/*Parapenia*/*villosa* (Fleutiaux, 1936)” [print], “MNHN/EC9284” [print].

**New material examined. CHINA: Guangxi Zhuang Autonomous Region:** 1 ex. (HBUM), Jiuren [久仁], Jiuwanshan N. R. [九万山自然保护区], Huanjiang County [环江县], Liuzhou City [柳州市], 1000 m, 3.VIII.2003, Xiu-Juan Yang leg.; 1 female (MYTC), Pohe Township [坡荷乡], Napo County [那坡县], Baise City [百色市], 1400 m, 26.VII.2024, Lu Qiu & Yu-Tang Wang leg.; 1 male (IZCAS), Defu Village [德孚村], Delong Township [德隆乡], Napo County, Baise City, 1350 m, 19.VI.2000, Jun Chen leg.; 1 female (IZCAS), Defu Village, Delong Township, Napo County, Baise City, 1350 m, 19.VI.2000, Wen-Zhu Li leg.; **Guizhou Province:** 1 male (MYTC), the botanical garden of Shishangsenlin Scenery Spot [石上森林景区植物园], Maolan N. R. [茂兰自然保护区], Wengang Township [翁昂乡], Libo County [荔波县], Qiannan Buyi and Miao Autonomous Prefecture [黔南布衣族苗族自治州], 16.VI.2019, Lu Qiu leg.; 1 male and 1 female (MYTC), ibid, but 21.VII.2018, Ming-Zhi Zhao leg; 1 male and 1 female (MYTC), Duoyinshan [多银山], Maolan N. R., Libo.

County, Qiannan Buyi and Miao Autonomous Prefecture, 20.VII.2018, Ming-Zhi Zhao leg.; 1 female (MYTC), Getou Village [格头村], Leigongshan Mountain [雷公山], Leishan County [雷山县], Qiandongnan Miao and Dong Autonomous Prefecture [黔东南苗族侗族自治州], 1030 m, 24.VII.2016, Ming-Zhi Zhao leg.; 1 male (MYTC), Leigongshan Mountain, Leishan County, Qiandongnan Miao and Dong Autonomous Prefecture, 1600–2100 m, 28–30.VII.2017, Zi-Hao Yang leg.; **Hainan Province:** 4 ex (MYTC), Bawangling Mountain [霸王岭], Cangjiang County [昌江县], 1.IX.2003, no collector recorded; 1 male (MYTC), Wuzhishan Mountain [五指山], Shuiman Township [水满乡], Wuzhishan City, 21.V.2014, Jian-Yue Qiu leg.; 1 male (MYTC), Mingfenggu [鸣凤谷], Jianfengling Mountain [尖峰岭], Ledong County [乐东县], 997 m, canopy fogging, 18.VIII.2010, Guo Zheng leg.; 1 female (MYTC), Tianchi [天池], Jianfengling Mountain, Ledong County, 970 m, 5.VIII.2023, Sheng-Tong Jin leg.; 3 females (PCJHo), “CHINA /HAINAN Is. /Minfeng Valey [Valley] /Jingfeng Tawnship [Jianfeng Township] /950 m”, “Ledong Li Autonomous /County. 18°44.3898′ N /108°50.3929′ E /12.vi.2018 /P. Viktora leg.”; 1 female (PCRK), “CHINA, Hainan /Jianfengling /Nature Reserve /09/05/2011” (PCRK); **VIETNAM: Vĩnh Phúc Province:** 1 male (ZISP), N Vietnam, Tamdao, 900 m, 30.V.1963, Kabakov; 3 males and 2 females (ZISP), “Вьетнам, курорт Там-Дао, 900 м., 2.9.1963 г., Кабаков [Vietnam, Tam Dao resort, 900 m, 2.IX.1963, O. N. Kabakov leg.]”; 1 male (ZISP), Vietnam-N (Tam Dao), 55 km NNW Hanoi, Tam Dao vill. env., 27.07–11.08.1998, h = 800–900 m, Lg. A. Napolov.; 1 ex. (PCJHo), “N Vietnam, 6.1991 /Vinh Phu distr. /Tam Dao, 900 m /Duong Tat Tu leg.”

**Diagnosis.** Body length 9.7–10.2 mm. Body stout (elytra/pronotum length ratio = 2.9–3.2:1, with some extreme cases can reach 3.7:1), yellowish brown to slightly reddish brown in general, abdomen and lateral margins of pronotum paler. Antennae dark brown, with basal two to three antennomeres yellowish brown. Legs and lateral margins of elytra yellow to yellowish brown (Figure 17). Pronotum subtrapezoidal. Lateral margins arched. Posterior angle of pronotum distinctly bidentate. Two notches present near base of each posterior angle on posterior margin. Intervals between punctures of pronotum oval, approximately 2–5 puncture diameters, sometimes intervals weakly wrinkled at lateral sides of disc (Figure 18A–C). Prosternal process shape variable, usually with large notch at ventral apex (Figure 18D–H). Elytral length/width ratio = 1.6–1.8:1; apical portion tapered, apice slightly pointed. Aedeagus typical of this genus, phallobase with basal margin straight, lateral corners usually protruded, forming two buds (Figure 19G–M). Bursa copulatrix with genus-typical spinous sclerotized plates (Figure 19E,F).

**Distribution.** China [**new record**]: Guangxi, Guizhou, Hainan; Vietnam: Vĩnh Phúc (Figure 39).

**Bionomics.** This species inhabits broad-leaved forests and can be collected by sweeping roadside vegetation (Figure 1A) or light trapping. It also has record of being collected by canopy fogging (one specimen from Hainan, Mingfenggu collected by Guo Zheng).

**Remarks.** This species has a short and broad body with relatively pale coloration, and its legs and elytral margins are near yellowish (Figure 17). It exhibits some morphological variability, such as the presence or absence of weak wrinkles between pronotal punctures and the sharpness of elytral apex. Suzuki [1] regarded the presence of wrinkles on pronotum as an important diagnostic character for this species. However, based on the examination of additional specimens, we found that this character is not stable. Even in individuals exhibiting such wrinkles, the degree of wrinkling is very weak and difficult to observe. Moreover, similar weak wrinkles have also been observed in *P. wulingshanensis*, which was described after Suzuki’s [1] study.

This species also exhibits geographical variation in body size. Among the specimens examined, individuals from northern Vietnam (Figure 17A–J), Hainan (Figure 17K,L), and the border region of western Guangxi typically exhibit a relatively short and robust body, with an elytra/pronotum length ratio of 2.9–3.2, representing the typical form of the species (Figure 17A–L). In contrast, specimens from Southern Guizhou and northern Guangxi show greater variation in body shape, with the elytra/pronotum length ratio reaching up to 3.7 in some individuals (n = 3) (Figure 17M,N).

As in *P. tonkinensis*, the male aedeagus and prosternal process of *P. wulingshanensis* conform to the typical form of the genus but exhibit considerable variation (Figure 18D–H and Figure 19G–M). Remarkable differences in both the shape and size of the parameres and phallobase have been observed even among specimens from the same locality. For example, the aedeagi shown in Figure 19I and Figure 19J correspond to specimens in Figure 17I and Figure 17G, respectively, both collected from Vietnam, Tam Dao. Although these two specimens are nearly identical in external morphology, their aedeagi differ significantly, representing a rather extreme case of intraspecific variation.

*P. wulingshanensis* is likely the closest relative of *P. villosa*. The two species are externally indistinguishable. Notably, the male genitalia of *P. wulingshanensis* exhibits a unique morphology within *Parapenia*, distinguishing it from *P. villosa* and all other congeners (see Remarks under *P. wulingshanensis*). At present, this character provides the only reliable basis for separating the two species.

**Figure 20:**
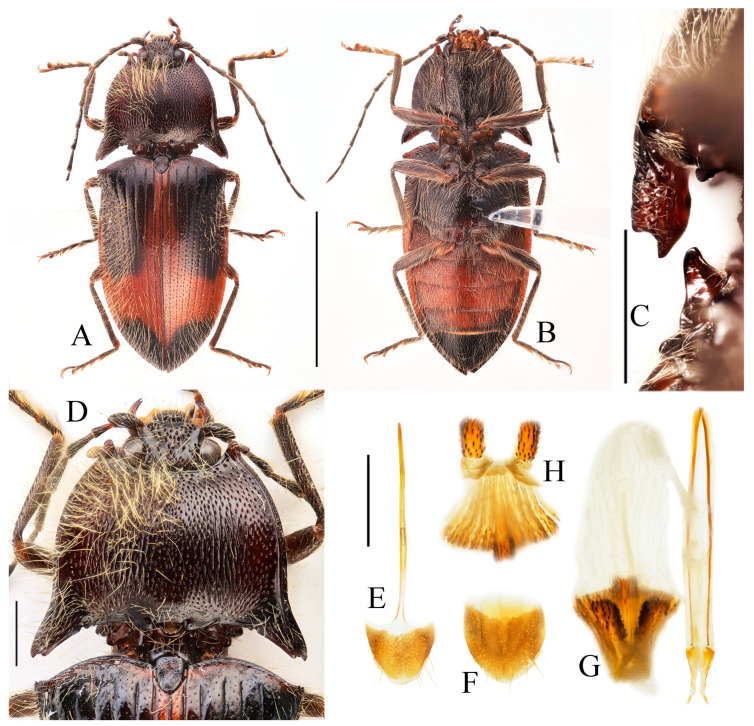
*Parapenia wuchaoi* sp. nov., female, holotype from Yunnan, Dulongjiang. (**A**) Dorsal view; (**B**) ventral view; (**C**) prosternal process, lateral view; (**D**) head and pronotum, dorsal view; (**E**) sternite VIII, ventral view; (**F**) tergite VIII, dorsal view; (**G**) ovipositor and genital tract, ventral view; (**H**) sclerotized part of bursa copulatrix. Scale bars 5 mm for (**A**,**B**); 1 mm for (**C**–**H**).

***Parapenia wuchaoi* sp. nov.**
ZooBank LSID: urn:lsid:zoobank.org:act:64723B20-5F4A-451F-8877-CE8CF39031B2
Figure 1D, Figure 20A–H and Figure 38

**Chinese common name.** 吴氏长须叩甲

**Type locality.** China: Yunnan: Nujiang Lisu Autonomous Prefecture, Gongshan County, Dulongjiang Township, Qinlangdang, 1350 m.

**Type material. Holotype: CHINA: Yunnan Province:** 1 female (MYTC), Qinlangdang [钦郎当], Dulongjiang Township [独龙江乡], Gongshan County [贡山县], Nujiang Lisu Autonomous Prefecture, 1350 m, 4–7.IX.2023, Chao Wu leg.

**Diagnosis and Comparison.** This is a well-recognized species that can be unequivocally distinguished from its congeners by its characteristic elytral color pattern and the presence of strong longitudinal wrinkles on pronotum (Figure 20A,B,D).

**Description.** Holotype, female: body length 10.8 mm; width 4.2 mm; antenna length 6.8 mm; pronotum length × width = 2.3 × 4.3 mm; elytra length 7.0 mm.

Body stout (elytra/pronotum length ratio = 3.0:1), bicolored, densely covered with long pubescence. Elytra and abdomen bicolored; elytra with suture from base to distal fifth dark reddish brown, and with wide dark reddish-brown transverse band near distal three-fourths; elytral epipleuron and lateral margins medially reddish brown. Legs dark brown, slightly reddish around trochanters. Abdomen dimly reddish brown, with lateral portions of each ventrite dark brown; apical margin of ventrite IV and entire ventrite V dark-brown. Remaining parts of body brownish-black. Pubescence whitish-yellow (Figure 20A,B).

Head smooth, nearly flat, with a large but shallow V-shaped depression. Anterior margin of frons slightly protruding medially. Frons with large, unevenly distributed umbilicate punctures; intervals between punctures along V-shaped depression and at head base very small, nearly confluent or less than one puncture diameter. Area at edge of V-shaped depression almost impunctate. Labrum with small umbilicate punctures mainly on distal half. Antennae reaching slightly before middle of elytra; antennomere II/III ratio = 1:1.5 (Figure 20D).

Pronotum curvilinearly trapezoidal (Figure 20D). Anterior angles stoutly protruding, apices rounded. Lateral margins distinctly arched medially. Posterior angles distinctly bidentate: outer tooth sharply narrowed, inner tooth quadrate. Two small notches present near each posterior angle on the posterior margin, the outer one larger. Pronotal surface densely covered with longitudinal elevated wrinkles, gradually flattening laterally. Punctures dense, especially along midline; intervals equal to or smaller than one puncture diameter. Toward lateral margins, punctures sparser.

Chin-piece with umbilicate punctures, intervals less than one puncture diameter. Middle of prosternum shallowly wrinkled with small punctures; intervals approximately equal to one puncture diameter. Prosternal process with distinct median carina, ventral apex with large notch (shape as in Figure 20C). Hypomeron with oval punctures; intervals subequal to or slightly less than one puncture diameter. Metaventrite with dense punctures; intervals slightly exceed one or reach two puncture diameters. Abdomen with punctures size and spacing similar to those of metaventrite. Abdominal ventrite V with apex blunt. Scutellar shield widest at base, longer than wide at widest points; basal margin slightly convex medially; apical portion narrowed, apex rounded. Elytral length/width ratio = 1.7:1; apices narrowed and blunt. Elytral shoulders as wide as the widest area near middle. Interstriae sparsely punctured with a mix of large and small punctures.

Tergite VIII semi-oval, apex rounded (Figure 20F). Sternite VIII semi-oval; spiculum ventrale slender, about 3.2 times longer than sternite VIII length (Figure 20E). Bursa copulatrix large, with genus-typical spinous sclerotized plates (Figure 20G,H).

Male unknown.

**Distribution.** China: Yunnan (Figure 38).

**Bionomics.** The holotype of this new species was found at night resting on the leaves of shrubs (Chao Wu, personal communication) (Figure 1D).

**Etymology.** Named after Mr. Chao Wu [吴超] (Beijing), the collector of this new species.

**Figure 21:**
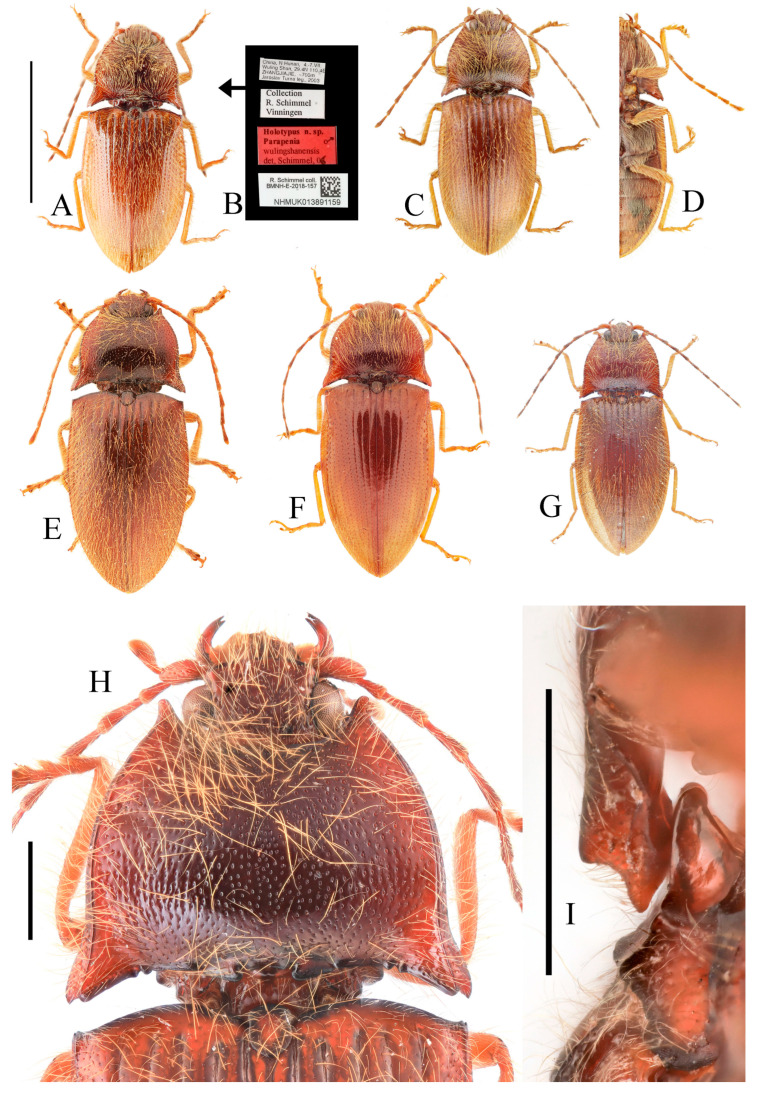
*Parapenia wulingshanensis.* (**A**,**B**) Holotype, male: (**A**) dorsal view; (**B**) labels; (**C**,**D**) paratype, female: (**C**) dorsal view; (**D**) ventral view; (**E**) male from Guangxi, Damingshan Mountain, dorsal view; (**F**,**G**) individuals from Guangdong, Nanling Mountain, dorsal view: (**F**) male; (**G**) female; (**H**) head and pronotum, male from Hunan, Dupangling mountain, dorsal view; (**I**) prosternal process, same male from Dupangling, lateral view. (**A**,**B**) photographed by Keita Matsumoto, copyright BMNH. Scale bars 5 mm for (**A**–**G**); 1 mm for (**H**,**I**).

**Figure 22:**
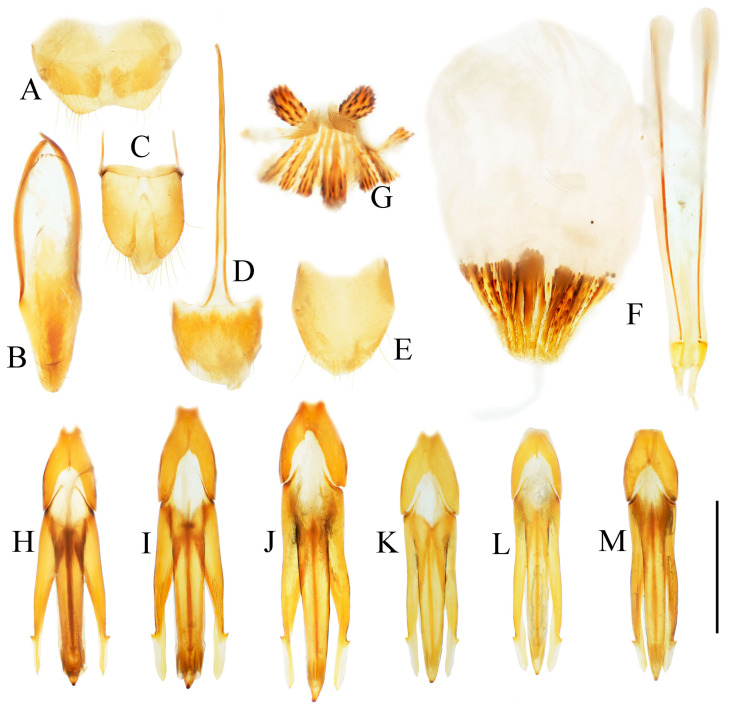
Pregenital segments and genitalia of *Parapenia wulingshanensis*, male ((**A**–**C**) from Guangdong, Nanling Mountain, (**H**–**M**) from various localities) and female ((**D**–**G**) from Nanling Mountain). (**A**) Sternite VIII and tergite VIII, ventral view; (**B**) sternite IX, dorsal view; (**C**) tergites IX–X, dorsal view; (**D**) sternite VIII dorsal view; (**E**) tergite VIII, dorsal view; (**F**) ovipositor and genital tract, ventral view; (**G**) sclerotized part of bursa copulatrix; (**H**–**M**) aedeagi, ventral view: (**H**,**I**) Nanling Mountain; (**J**) Guangxi, Damingshan Mountain; (**K**) Hunan, Mangshan N.R.; (**L**) Fujian, Dehua; (**M**) Zhejiang, Fengyangshan mountain. Scale bar 1 mm.

***Parapenia wulingshanensis* Schimmel, 2006**
Figure 1B, Figure 21A–I, Figure 22A–M and Figure 39

*Parapenia wulingshanensis* Schimmel, 2006: 153 [11] (original description); Kundrata et al. 2018: 34 [3] (catalogue).

**Chinese common name.** 武陵长须叩甲

**Type locality.** China: N Hunan: Wuling Shan, Zhangjiajie, 700 m, 29.4 N, 110.4 E [湖南武陵山张家界].

**Type material examined. Holotype** of *Parapenia wulingshanensis* Schimmel, 2006: male (NHMUK), “China, N Hunan, 4–7. VII./Wuling Shan, 29.4N 110.4E/ZHANGJIAJIE, ~700 m/Jaroslav Turna leg., 2003” [print], “Collection/R. Schimmel/Vinningen” [print], “Holotypus n. sp./Parapenia ♂/wulingshanensis/det. Schimmel 06” [print in red background, with handwriting for “♂” and “6”], “R. Schimmel coll./BMNH-E-2018-157/NHMUK013891159” [print, with a QR code]; **paratypes** of *Parapenia wulingshanensis* Schimmel, 2006: 3 males and 2 females (1 female in DEMSU; the rest in NHMUK), same data as holotype, NHMUK specimens with numbers NHMUK013891160–013891163.

**Material examined. CHINA: Anhui Province:** 2 males and 1 female (MYTC), Hougu Scenery spot [猴谷景区], Huangshan Mountain [黄山], Taokou Town [汤口镇], Huangshan City [黄山市], 530 m, 12–13.VII.2013, Hai-Tian Song leg.; **Fujian Province:** 1 male (MYTC), Shuikou Town [水口镇], Dehua County [德化县], Quanzhou City [泉州市], 650 m, 10.VII.2020, Yu-Chen Zheng leg.; 1 ex. (IZCAS), Lishan [里山], Longqishan N. R. [龙栖山自然保护区], Jiangle County [将乐县], Sanming City [三明市], 650 m, 17.VIII.1991, Xiao-Chun Chuan leg.; 1 male (SZPU), Buyun Township [步云乡], Shanghang County [上杭县], Longyan City [龙岩市], 21.VII.1988, Bang-Kan Huang leg.; 1 male (SZPU), Meihuashan N. R. [梅花山自然保护区], Longyan City [龙岩市], 8.IX.2001, Ming-Yi Tian leg.; 1 ex. (HBUM), Wuyishan N. R. [武夷山自然保护区], Nanping City [南平市], 21.VII.2003, Guo-Dong Ren & Ming Bai leg.; 1 male (HBUM), Guadun [挂墩], Wuyishan N. R. [武夷山自然保护区], Nanping City [南平市], 18.VII.2003, Ming Bai leg.; 1 male (MYTC), ibid, but 1000 m, 20.VIII.2020, Han Gao leg.; **Hunan Province:** 1 ex. (MYTC), Fangkongshao [防空哨], Dupangling N. R. [都庞岭自然保护区], Qianjiadong Township [千家峒乡], Jiangyong County [江永县], Yongzhou City [永州市], 633 m, 29.VIII.2020, Lu Qiu leg.; 1 ex. (MYTC), Fangkongshao, Dupangling N. R., Qianjiadong Township, Jiangyong County, Yongzhou City, 633 m, 29.VIII.2020, Wei Cao leg.; 1 female (MYTC), Tianpingshan [天平山], Wudaoshui Town [五道水镇], Sangzhi County [桑植县], Zhangjiajie City [张家界市], 1400 m, 30–31.VII.2018, Jian-Yue Qiu & Hao Xo leg.; 1 female (HBUM), Jiemuxi [借母溪], Yuanling County [沅陵县], Huaihua City [怀化市], 2–11.VIII.2004, Jian-Feng Wang & Ji-Liang Wang leg.; 1 male and 1 female (MYTC), Xiangsikeng [相思坑], Mangshan Mountian [莽山], Yizhang County [宜章县], Chenzhou City [郴州市], 1430 m, light trap, Ren-Zhi Zhang leg.; **Guangdong Province:** 16 ex (MYTC), Ruyang [乳阳], Nanling N. R. [南岭自然保护区], Shaoguan City [韶关市], Guangdong Province, 980 m, 5.IX.2020, Qing-Bo Huo leg.; 1 ex. (SZPU), Babaoshan [八宝山], Nanling N. R., Shaoguan City, 4.X.2013, Zhen Qin leg.; 1 male (MYTC), ibid, but 1100 m, 15.VI.2024, Ming-Zhi Zhao leg.; 4 males and 7 females (SZPU), Nanling N. R., Shaoguan City, VII–VIII.2008, Lei Gao & Kai-Xuan Chen leg.; 1 male (SZPU), Nanling N. R., Shaoguan City, IV–V.2008, Lei Gao & Kai-Xuan Chen leg.; 1 male (SZPU), Nanling N. R., Shaoguan City, 17.VI.2009, Ding Chen & Zi-Ye Meng leg.; 1 male (SZPU), Nanling N. R., Shaoguan City, 21.VI.2009, Ding Chen & Zi-Ye Meng leg.; 1 ex. (SZPU), Nanling N. R., Shaoguan City, 18.VI.2009, Ding Chen leg.; 1 female (MYTC), Chebaling N.R. [车八岭自然保护区], Shaoguan City, 1–5.X.2024, Ming-Zhi Zhao leg.; **Guangxi Zhuang Autonomous Region:** 1 female (MYTC), Maoershan N. R. [猫儿山自然保护区], Guilin City [桂林市], 475–1158 m, 25–27.VIII.2020, Lu Qiu leg.; 1 male (HBUM), Damingshan Mountain [大明山], Nanning City [南宁市], 5–9.VIII.2011, Zhen-Xing Zhang & Bin-Ji Liang leg.; 1 male and 1 female (MYTC), ibid, but 14.IX.2022, no collector recorded; 2 males (MYTC), Shengtangshan Mountain [圣堂山], Dayaoshan N.R. [大瑶山自然保护区], Jinxiu County [金秀县], Laibin City [来宾市], 1200 m, 18.VII.2025, Lu Qiu leg.; 2 females (MYTC), ibid, but Yu-Tang Wang leg.; 2 males and 2 females (MYTC), ibid, but 700 m, 19.VII.2025, Gan-Yan Yang & Hong-Liang Shi leg.; 1 male (MYTC), ibid, but 1173 m, 18.VII.2025, Gan-Yan Yang & Hong-Liang Shi leg.; 1 female (MYTC), ibid, but 1200 m, 19.VII.2025, Guan-Lin Xie leg.; 2 males and 3 females (MYTC), Shengtang Grand Canyon [圣堂大峡谷], Dadeng [大凳], Liuxiang Township [六巷乡], Jinxiu County, Laibin City, 1031 m, 22.VII.2025, Gan-Yan Yang & Hong-Liang Shi leg.; 1 male (MYTC), Baleshan Mountain [巴勒山], Jinxiu County, Laibin City, 950 m, 12.VII.2025, Hong-Liang Shi leg.; 8 males and 3 females (MYTC), Yinshan Park [银衫公园], Dayaoshan N.R., Jinxiu County, Laibin City, 1200–1300 m, 15.VII.2025, Hong-Liang Shi leg.; 2 males (MYTC), ibid, but 1200 m, Lu Qiu leg.; **Jiangxi Province:** 1 female (IZCAS), Jiulianshan N. R. [九连山自然保护区], Ganzhou City [赣州市], 30.IX.1979, no collector recorded; **Zhejiang Province:** 1 male and 1 ex. (HBU), Fengyangshan N. R. [凤阳山自然保护区], Lishui City [丽水市], 25.VII–1.VIII.2007, Hao-Yu Liu & Zhen-Hua Gao leg.; 1 ex. (MYTC), Fengyangshan N. R., Lishui City, 27.VIII.2019, Hong-Yi Zheng-Xu leg.

**Diagnosis.** The external morphology is almost indistinguishable from that of *P. villosa*. Body length 8.5–11.4 mm. Elytra/pronotum length ratio = 3.0–3.2:1. Apex of elytron weakly pointed. Abdominal ventrite V triangularly narrowed apically, apex rounded. Scutellar shield widest at base.

**Distribution.** China: Anhui [**new record**], Fujian [**new record**], Guangdong [**new record**], Guangxi [**new record**], Hunan, Jiangxi [**new record**], Zhejiang [**new record**] (Figure 39).

**Bionomics.** This species inhabits broad-leaved forests and is commonly collected during the day by sweeping roadside vegetation. It has also been observed visiting flowers of Fagaceae during daylight hours (Gan-Yan Yang & Hong-Liang Shi, personal communication). At night, it is attracted to light (Figure 1B) and may be found crawling on shrub leaves.

**Remarks.** This is a quite common species in South and East China. There are no significant external differences between *P. wulingshanensis* and *P. villosa*, as both species have stout body and similar coloration. Initially, we considered the two species to be conspecific. However, a comparison of the aedeagus of the holotype of *P. wulingshanensis* with that of the topotype specimens of *P. villosa* revealed that they are different species. The aedeagus of *P. wulingshanensis* exhibits distinctive characters that clearly differentiate it from all other congeners. Its parameres are robust, curved, and distinctly tapered, with the apical hooked portion positioned far from the paramere tip (Figure 22H–K). Its phallobase is markedly V-shaped, with a strongly protruding basal region and a median concavity (Figure 22H–K). In contrast, other *Parapenia* species (including *P. villosa*) possess slender parameres with the hook situated closer to the apex, along with a smaller phallobase that typically shows truncate or slightly convex basal margin.

Similarly to *P. villosa* and *P. tonkinensis*, this species also exhibits substantial variation in the aedeagus among individuals (Figure 22H–M). Marked differences can be observed even among specimens from the same locality (e.g., specimens from Nanling N. R., Figure 22H,I). A general clinal trend is evident: populations from Center Guangxi (Figure 22J) to the Nanling Mountain ranges (Figure 22H,I) have a robust aedeagus with elongated apical hooked portion and strongly protruding V-shaped phallobase. While it progressively shows smaller size and reduced robustness in both parameres and phallobase when shifting eastward to Zhejiang and Fujian provinces (Figure 22L,M), In other words, populations from Fujian, Zhejiang, and nearby regions possess aedeagi that more closely resemble the typical form of the genus. Nevertheless, the relatively elongated apices of the parameres and the strongly protruding phallobase indicate that these populations all belong to the current definition of *P. wulingshanensis*.

**Figure 23:**
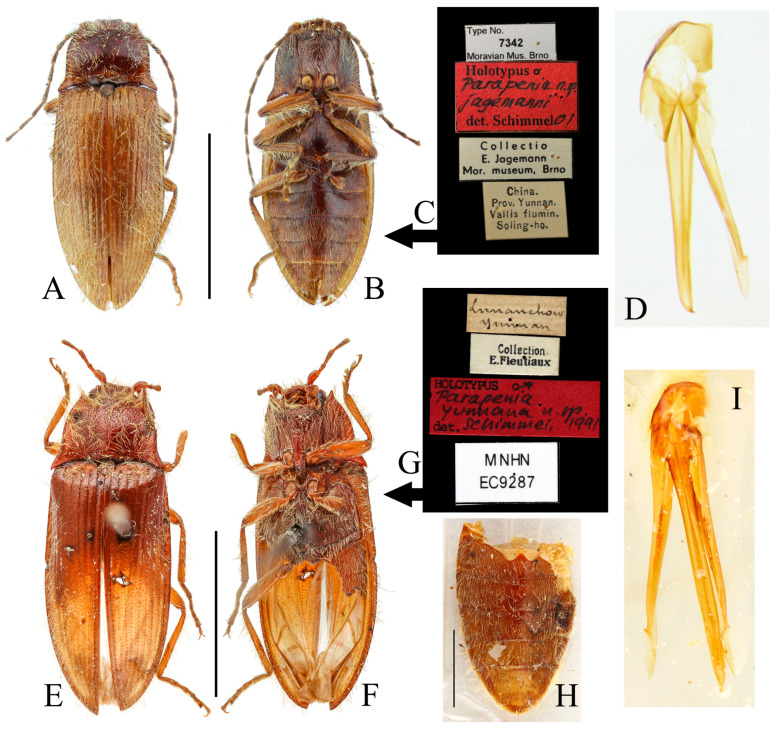
*Parapenia yunnana*. (**A**–**D**) Holotype of *P. jagemanni* syn. nov., male: (**A**) dorsal view; (**B**) ventral view; (**C**) labels; (**D**) aedeagus, dorsal view; (**E**–**I**) holotype of *P. yunnana*, male: (**E**) dorsal view; (**F**) ventral view; (**G**) labels; (**H**) abdomen, ventral view; (**I**) aedeagus, ventral view. (**A**,**B**,**D**) photographed by Gabriela Pačková; (**C**) photographed by Jiří Procházka, copyright MZMB; (**E**–**I**) photographed by Christophe Rivier, copyright MNHN. Scale bars 5 mm for (**A**,**B**,**E**,**F**); 2 mm for (**H**); (**D**,**I**) not to scale.

***Parapenia yunnana* Schimmel, 1993**
Figure 23A–I and Figure 38

*Parapenia yunnana* Schimmel, 1993: 249 [7] (original description); Schimmel 1996: 160 [2] (diagnosis); Schimmel 2006: 153 [11] (misspelling as “*yunnanensis*”); Cate 2007: 185 [29] (catalogue); Kundrata et al. 2018: 34 [3] (catalogue); Jiang & Yang 2023: 45 [31] (catalogue).

*Parapenia jagemanni* Schimmel, 2001: 211 [10] (original description); Cate 2007: 184 [29] (catalogue); Kundrata et al. 2018: 32 [3] (catalogue); Jiang & Yang 2023: 45 [31] (catalogue). **Syn. nov.**

**Chinese common name.** 云南长须叩甲

**Type locality.** China: Yunnan: Lunanchow [Lunan Zhou/路南州, =Shilin County in Kunming City/昆明市石林县].

**Type material examined. Holotype** of *Parapenia yunnana* Schimmel, 1993, male (MNHN), “HOLOTYPUS [print] ♂/*Parapenia* [handwriting] /*yunnana* n. sp. [handwriting]/det. [print] Schimmel, 1991 [handwriting]”, “Collection [handwriting]/E. Fleutiaux [print]”, “lunanchow [=Lunan Zhou, now Shilin County near Kunming City]/yunnan” [handwriting], “MNHN EC9287” [print]. **Holotype** of *Parapenia jagemanni* Schimmel, 2001: male (MZMB), “Holotypus ♂ [print]/Parapenia n. sp. [handwriting]/jagemanni [handwriting]/det. Schimmel [print] 01 [handwriting]”, “Collectio/E. Jagemann/Mor. Museum, Brno” [print], “China./Prov. Yunnan,/Vallis flumin./Soling-ho.” [print], “Type No./7342/Moravian Mus. Brno” [print].

**Diagnosis.** Body length 8.8–10.0 mm, width 3.3 mm (elytra) (Schimmel 1993; 2001). Head, antennae, pronotum, scutellar shield, ventral side brown; elytra and legs yellow to yellowish brown (Figure 23A,B,E,F). Elytra/pronotum length ratio = 4.0–4.1:1. Elytral length/width ratio = 2.0:1. Elytra with apices together blunt. Abdominal ventrite V rounded apically (Figure 23H).

**Distribution.** China: Yunnan (Figure 38).

**Bionomics.** Unknown.

**Remarks.** This species possesses extremely elongate elytra and a short, small pronotum, resulting in a distinctly slender body. The elytra/pronotum length ratio exceeds 4.0, and the elytral length/width ratio reaches 2.0, which is exceptionally rare within the genus *Parapenia*. Most species in the genus have a relatively stout body, with elytra/pronotum ratios not exceeding 3.7 and elytral length/width ratio mostly ranging from 1.6 to 1.8. The only species with a comparable body form is *Parapenia nyuwa* sp. nov., which also exhibits an elytral length/width ratio of 2.0. However, *P. nyuwa* is distinctly bicolored (body black to blackish brown, with light-colored pronotal posterior angles; elytra yellow, with narrow, inconspicuous black margin along the edges) and features a longer pronotum (elytra/pronotum length ratio 3.8–3.9), in contrast to the nearly uniformly yellowish-brown body and shorter pronotum of the present species.

Based on the similar elytra/pronotum length ratios (4.1 in *P. jagemanni* versus 4.0 in *P. yunnana*), we consider *P. jagemanni* to be a junior synonym of the latter, despite the holotype of *P. jagemanni* having somewhat paler elytra. The two species also occur in geographically proximate regions in central Yunnan. *Parapenia jagemanni* was described based a male specimen collected from “Vallis flumin, Soling-ho,” now identified as the Longchuanjiang River valley [龙川江河谷] near Yuanmou County [元谋县], Chuxiong Yi Autonomous Prefecture. The holotype of *P. yunnana* was collected from Shilin County, near Kunming City. Schimmel [10] noted no visible hooks on the parameres of *P. jagemanni*. However, upon reexamination of the holotype housed in MZMB, we found that the specimen does possess hooks on parameres (Figure 23D), consistent with *P. yunnana* (Figure 23I) and all other species of *Parapenia*.

**Figure 24:**
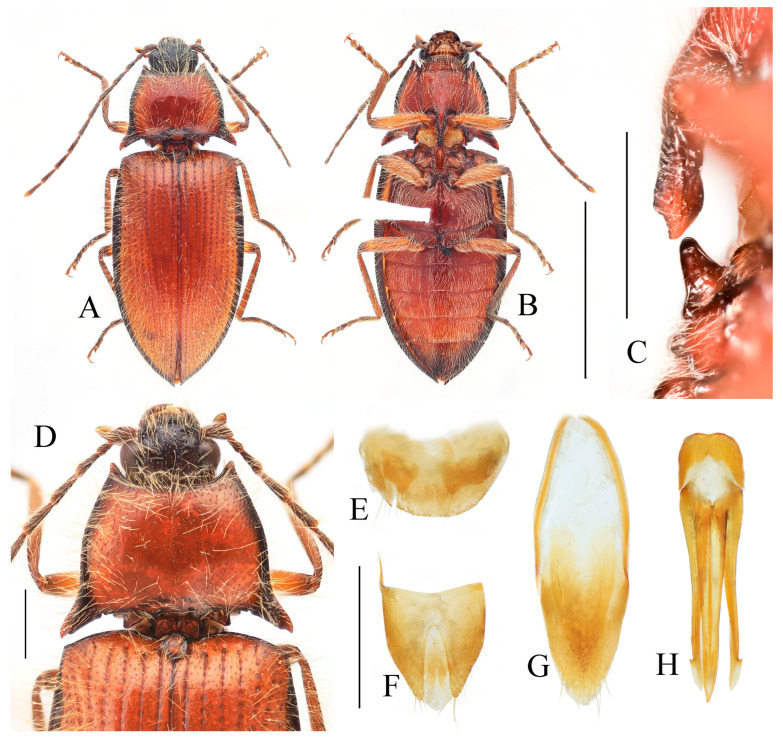
*Parapenia zhengi* **sp. nov.**, male, holotype from Xizang, Zayü. (**A**) Habitus, dorsal view; (**B**) same, ventral view; (**C**) prosternal process, lateral view; (**D**) head and pronotum, dorsal view; (**E**) sternite VIII and tergite VIII, ventral view; (**F**) tergites IX–X, dorsal view; (**G**) sternite IX, dorsal view; (**H**) aedeagus, ventral view. Scale bars 5 mm for (**A**,**B**); 1 mm for (**C**–**H**).

***Parapenia zhengi* sp. nov.**
ZooBank LSID: urn:lsid:zoobank.org:act:1DB583BA-3114-4362-86D5-0D0FE9B7F182
Figure 24A–H and Figure 38

**Chinese common name.** 郑氏长须叩甲

**Type locality.** China: Xizang: Nyingchi City, Zayü County, Way from Upper Zayü Town to Lower Zayü Town, 1600–1900 m.

**Type material. Holotype:** male (MYTC), Way from Upper Zayü Town to Lower Zayü Town [下察隅镇], Zayü County, Nyingchi City, Xizang Autonomous Region, China, 1600–1900 m, 9–10.VII. 2016, Jian-Yue Qiu & Hao Xu leg.; **Paratype:** 1 male (MYTC), Upper Zayü Town, Zayü County, Nyingchi City, Xizang Autonomous Region, China, 2000 m, 11.VIII. 2020, Yu-Chen Zheng leg.

**Diagnosis and comparison.** Bicolored species with overall reddish-brown body; head, antennae, and apical portion of abdomen black; margins of pronotum and elytra black; legs yellow (Figure 24A,B). Head slightly convex. Pronotum with single small notch near each posterior angle on posterior margin. Elytral/pronotum length ratio = 3.7:1. Apex of elytron spinous. Abdominal ventrite V triangularly narrowed apically, apex blunt. Scutellar shield widest at base. Tergite VIII semi-oval, apical margin broadly convex.

This new species resembles *Parapenia nigroapicalis* Suzuki, 1982, *Parapenia pangu* sp. nov., and *Parapenia ruihangi* sp. nov. in having sharply spinous elytral apices. However, it can be readily distinguished from the latter three by its unique coloration: pronotum and elytra margined with black, head and apical portion of abdomen black, and legs yellow.

**Description.** Holotype, male: body length 9.8 mm; width 3.8 mm; antenna length 5.9 mm; pronotum length × width = 1.9 × 3.4 mm; elytra length 6.8 mm.

Body stout (elytra/pronotum length ratio = 3.6:1), bicolored, narrow, covered with moderately long pubescence. Head including antennae, mandibles, and maxillary palpi black. Pronotum, scutellar shield, and elytra reddish brown, with black margins; anterior and posterior angles of pronotum black, with apices slightly paler. Ventral side reddish brown, with all sclerites (e.g., prosternum, hypomeron, mesonotum, metaventrite, metacoxal plates, lateral margins of abdomen) bordered with black. Legs yellowish brown, joints between femora and tibiae darkened. Pubescence yellow (Figure 24A,B).

Head smooth and slightly convex; median depression indistinct. Anterior margin of frons slightly arched. Frons with evenly distributed small punctures, puncture intervals approximately 1–2 puncture diameters. Labrum coarsely punctate throughout. Antennae reaching slightly before middle of elytra; antennomere II/III ratio = 1:1.7 (Figure 24D).

Pronotum subtrapezoidal (Figure 24D). Anterior angles triangularly protruding, slightly outward, apices rounded. Lateral margins slightly arched at middle. Posterior angles indistinctly bidentate; outer tooth with rounded lateral margin and sharp apex; inner tooth reduced, forming shallow notch with outer tooth. One small notch present near posterior angle. Pronotal punctures separated by flat and smooth intervals, about 5–6 puncture diameters wide.

Chin-piece densely punctate with small umbilicate punctures; intervals wrinkled and less than one puncture diameter. Median area of prosternum smooth, with sparser and smaller punctures, puncture intervals about 3–4 diameters. Prosternal process with indistinct median carina; ventral-apically with large notch (Figure 24C). Hypomeron punctate, with punctures becoming sparser laterally; puncture intervals less than one diameter medially, about two diameters laterally. Metaventrite with small punctures, intervals about two diameters. Abdominal punctation similar to metaventrite, with puncture intervals 1–2 puncture diameters. Abdominal ventrite V triangularly narrowed apically, apex rounded. Scutellar shield widest at base, as long as wide, apex rounded. Elytral length/width ratio = 1.8:1; elytral/pronotum length ratio = 3.6:1; interstriae with sparse, irregularly sized punctures. Apices of elytra distinctly spinous, with spines directed inward.

Tergite VIII semi-oval, apical margin broadly convex (Figure 24E). Sternite VIII with darkened areas bearing protrusions on inner sides of distal margin (Figure 24E). Tergite IX with sides straight and subparallel in basal half, abruptly curved near middle; distal half narrowed apically with straight lateral margins (Figure 24F). Sternite IX elongate, 2.9 times longer than wide (Figure 24G).

Aedeagus with sides of median lobe narrowed from base to middle, slightly expanded subapically; apex forming a small blunt tip. Paramere with lateral sides concave in middle; apical portion beyond subapical hook 2.8 times longer than wide, apex sharply tapered. Basal angles of phallobase rounded (Figure 24H).

Male paratype: similar to holotype in general. Body length 9.7 mm.

Female unknown.

**Distribution.** China: Xizang (Figure 38).

**Bionomics.** Unknown.

**Etymology.** The new species is named in honor of Dr. Yu-Chen Zheng [郑昱辰] (China Agricultural University), a friend of the first author, in recognition of his generous support for this research, including the donation of *Parapenia* specimens.

### 3.2. Descriptions of New Genera Connected with Parapenia

The term “connected” used here does not refer to a phylogenetic relationship, as the tribe Dimini still lacks a comprehensive phylogenetic framework. The association of new genera with *Parapenia* is based on morphological similarities or historical taxonomic placements.

*Parapenioides* **gen. nov.** shares certain external characters with *Parapenia*, such as bidentate posterior angles of pronotum and anterior angles of elytra. However, it differs in lacking the prominent protruding anterior angles of pronotum which are typical for *Parapenia*. *Sinopenia* **gen. nov.**, established based on *Parapenia significata* Schimmel, 1998, is similar to *Parapenioides* in possessing bidentate posterior angles of pronotum and anterior angles of elytra, and likewise lacks pronounced anterior angles of pronotum. Its prosternal process is distinctly widened dorsoventrally, featuring a broadened apical notch, which differs markedly from those of *Parapenia* and *Parapenioides*. All three above-mentioned genera can be further separated by their genital structures.

**Figure 25:**
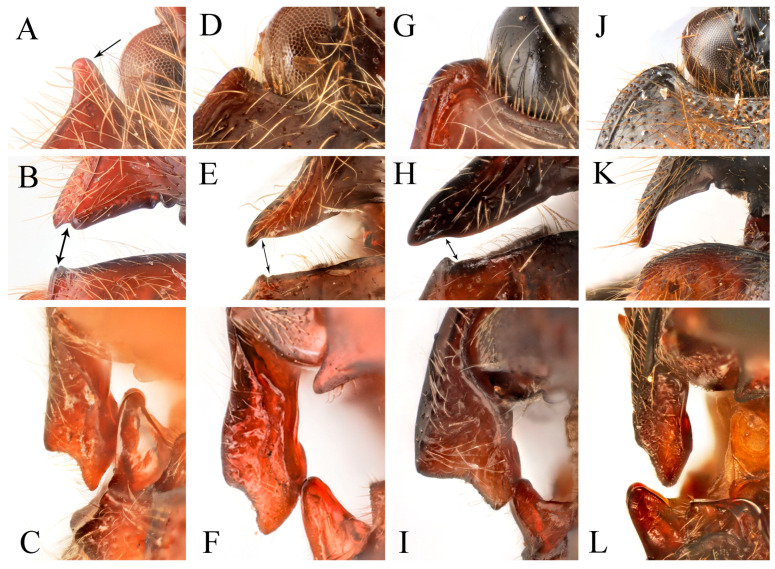
Distinctive characters of *Parapenia* (**A**–**C**), *Parapenioides* **gen. nov.** (**D**–**F**), *Sinopenia* **gen. nov.** (**G**–**I**), and *Megapenia* **gen. nov.** (**J**–**L**). (**A**,**D**,**G**,**J**) Anterior angle of pronotum, arrow in (**A**) indicates the narrowly protruded part; (**B**,**E**,**H**,**K**) hind angle of pronotum, bidirectional arrows in (**B**,**E**,**H**) indicate the bidentate parts of hind angle of pronotum and anterior angle of elytron; (**C**,**F**,**I**,**L**) prosternal process. Not to scale.

In contrast, *Megapenia* **gen. nov.** does not possess any bidentate structures in pronotal posterior angles and elytral base. Its connection to *Parapenia* lies in its type species, *Parapenia marginalis* (Fleutiaux, 1942), which appears to have been mistakenly placed in *Parapenia*. This species was originally described under *Penia*, and along with *Penia cruciata* Bouwer, 1991, which remains in *Penia*, reflects a relatively complex historical taxonomic background. Despite these differences in external morphology, genital dissection reveals that *Megapenia* possesses strongly sclerotized plates in bursa copulatrix, a feature shared with *Parapenia*, *Parapenioides,* and *Sinopenia*. Nevertheless, the sclerotized plates demonstrate remarkable structural divergence among genera.

A comparative diagnosis of these genera is provided in Table 1 and Figure 25.

#### 3.2.1. *Parapenioides* gen. nov.

**Figure 26:**
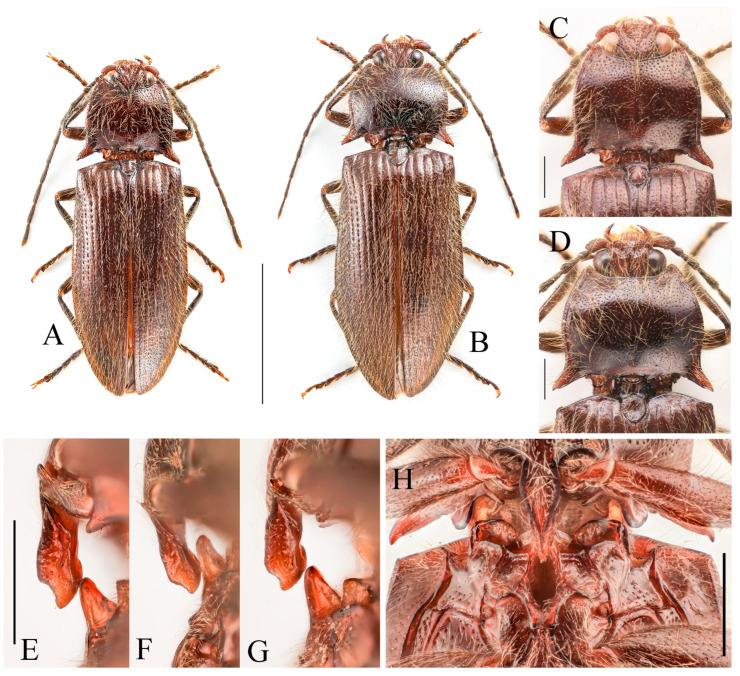
*Parapenioides zidani* **gen. & sp. nov.** from Sichuan, Dafengding. (**A**) Male, holotype, dorsal view; (**B**) female, paratype, ventral view; (**C**) male, holotype, head and pronotum, dorsal view; (**D**) female, paratype, head and pronotum, dorsal view; (**E**–**G**) prosternal process, lateral view: (**E**) male, holotype; (**F**) male, paratype; (**G**) female, paratype; (**H**) posterior part of prothorax and mesothorax, ventral view. Scale bars 5 mm for (**A**,**B**); 1 mm for (**C**–**H**).

**Figure 27:**
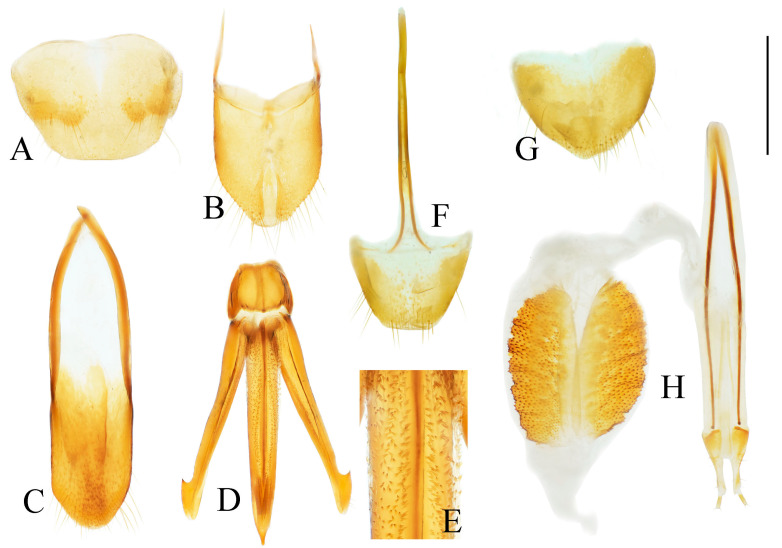
Pregenital segments and genitalia of *Parapenioides zidani* **gen. & sp. nov.**, male, holotype (**A**–**E**) and female, paratype (**F**–**H**) from Sichuan, Dafengding. (**A**) Sternite VIII and tergite VIII, ventral view; (**B**) tergites IX–X, dorsal view; (**C**) sternite IX, dorsal view; (**D**) aedeagus, ventral view; (**E**) microspines on the surface of median lobe; (**F**) sternite VIII, ventral view; (**G**) tergite VIII, dorsal view; (**H**) ovipositor and genital tract, ventral view. Scale bar 1 mm, (**E**) not to scale.

ZooBank LSID: urn:lsid:zoobank.org:act:6EBF9102-B10B-452F-83D9-5B89F9C8B672
Figure 25D–F, Figure 26A–H, Figure 27A–H and Figure 40

**Type species.** *Parapenioides zidani* **sp. nov.**; here designated.

**Chinese common name.** 拟长须叩甲属

**Diagnosis.** Antennomeres elongate filiform; antennomere II shortest, distinctly shorter than antennomere III. Pronotum with anterior angles normally constricted (Figure 25D). Posterior angles protruding laterally, apex indistinctly bidentate (Figure 25E), with a small notch near each posterior margin of posterior angle. Prosternal process with distinct median carina (Figure 26H), and ventral-apical notch (Figure 25F). Elytral anterior angles bidentate (Figure 25E). Tarsomeres III–IV lobed. Aedeagus with elongate median lobe and parameres; parameres expanded apically, with a distinct subapical hook. Ovipositor elongate. Bursa copulatrix with a pair of large, symmetrical, sclerotized plates (Figure 27H).

This genus resembles *Parapenia* and *Sinopenia* in having bidentate posterior angles of the pronotum and bidentate anterior angles of the elytra (Figure 25B,E,H). However, it can be readily distinguished from both by the structure of the anterior angles of the pronotum (Figure 25A,D,G), as well as by the morphology of the aedeagus and the sclerotized plates of the bursa copulatrix. For detailed differences, see Table 1.

**Etymology.** The generic name is derived from its morphological resemblance to *Parapenia*. Gender: masculine.

**Composition.** Monospecific, with *P. zidani* sp. nov. only.

**Distribution.** China (Figure 40).

***Parapenioides zidani* sp. nov.**
ZooBank LSID: urn:lsid:zoobank.org:act:37170BB3-C69A-4539-B4FF-5541BF542C0A
Figure 26A–H, Figure 27A–H and Figure 40

**Chinese common name.** 子聃拟长须叩甲

**Type locality.** China: Sichuan: Liangshan Prefecture, Mabian County, Yonghong Township, Dafengding Nature Reserve, Nieshijue, 1458 m.

**Type material. Holotype:** male (MYTC), Nieshijue [捏史觉], Dafengding Nature Reserve [大风顶自然保护区], Yonghong Township [永红乡], Mabian County [马边县], Liangshan Yi Autonomous Prefecture [凉山彝族自治州], Sichuan Province, China, 1458 m, 20–24.VIII.2023, Tian-Long He leg. **Paratypes:** 9 specimens: 3 males and 4 females (2 males and 3 females in MYTC; 1 male and 1 female in SZPU), same data as holotype; 1 male (CWNU), Guanmengou [关门沟], Wolong Nature Reserve [卧龙自然保护区], Wenchuan County [汶川县], Aba Zang and Qiang Autonomous Prefecture [阿坝藏族羌族自治州], Sichuan Province, China, 10.VIII.2015, Zhong-Hua Wei, Feng Wang & Hua Zhang leg.; 1 male (MYTC), Miaoping [庙坪], North-East of Huangguan Town [皇冠镇], Ningshan County [宁陕县], Ankang City [安康市], Shaanxi Province, China, 1469 m, 33.6091988°N, 108.45307799°E, 29.VIII.2024, Jia-Cheng Zhang leg.

**Diagnosis and comparison**. See above.

**Description.** Holotype, male: body length 12.2 mm; width 4.1 mm; antenna length 7.6 mm; pronotum length × width = 2.2 × 3.7 mm; elytra length 8.6 mm.

Body elongate (elytra/pronotum length ratio = 3.9:1), unicolorous, dark reddish brown, elongate, densely covered with long pubescence, erect on dorsal surface and semi-erect on ventral surface. Pubescence yellow (Figure 26A).

Head with large V-shaped depression. Anterior margin of frons slightly convex anteriorly, sharply carinate laterally, narrowly declined in middle. Frons densely punctate with large umbilicate punctures; puncture intervals uneven, usually 1–3 puncture diameters. Labrum with small, evenly distributed punctures. Antennae extending slightly beyond the middle of elytra; antennomere II/III ratio = 1:1.5 (Figure 26C).

Pronotum subtrapezoidal. Lateral margins slightly arched medially. Posterior angles narrow, with outer tooth curved backward apically; inner tooth reduced, forming a shallow notch with the outer tooth. Intervals between pronotal punctures flat and smooth, approximately 2–3 puncture diameters (Figure 26C).

Chin-piece, prosternum, hypomeron, and metaventrite with similar punctation to pronotum; puncture intervals about equal to or slightly greater than one puncture diameter. Prosternal process laterally shaped as in Figure 26E. Scutellar shield widest at base, as long as wide at the broadest point; basal margin convex, apical portion narrowed, apex blunt; surface with sparse micropunctures. Elytral length/width ratio = 2.1:1; Elytral striae distinctly grooved; interstriae sparsely punctate. Elytral apices each independently rounded.

Apex of abdominal ventrite V rounded. Abdominal tergite VIII subtrapezoidal, apical margin truncate medially (Figure 27A). Sternite VIII with two transverse darkened areas, inner sides of distal margin slightly protruding (Figure 27A). Tergite IX with sides straight and subparallel, abruptly curved distally; distal half with sides straight, tapering toward apices (Figure 27B). Sternite IX elongate, 4.0 times longer than wide (Figure 27C).

Aedeagus (Figure 27D,E): median lobe with sides subparallel, tapering apically to a small, blunt protrusion; surface bearing microspines. Paramere slender, with large subapical hook and rounded apex.

Male paratypes. Body length 11.1–12.2 mm, elytra/pronotum length ratio = 3.8–4.0:1. Similar to holotype male. Shape of prosternal process variable (Figure 26F).

Female paratypes. Slightly larger than males (Figure 26B); body length 12.9–13.0 mm, elytra/pronotum length ratio = 3.9–4.1:1. Pronotum with more convex lateral margins in comparison to males (Figure 26D). Shape of prosternal process variable (Figure 26G). Abdominal tergite VIII with blunt apex (Figure 27G); sternite VIII semioval, spiculum ventrale 3.0 times longer than sternite VIII length (Figure 27F). Ovipositor overall 5.6 times longer than coxite length. Styli distinct, cylindroid, apically attached. Bursa copulatrix with a pair of large, flat sclerotized plates; surface densely covered with small spines (Figure 27H).

**Distribution.** China: Sichuan and Shaanxi (Figure 40).

**Bionomics.** Adults can be collected by light trapping.

**Etymology.** The species is named in honor of Mr. Zi-Dan Xu [许子聃] (Chengdu, China), the colleague of the collector of the holotype, Mr. Tian-Long He, in recognition of his support and assistance during the collecting expedition to Dafengding.

#### 3.2.2. *Sinopenia* gen. nov.

**Figure 28:**
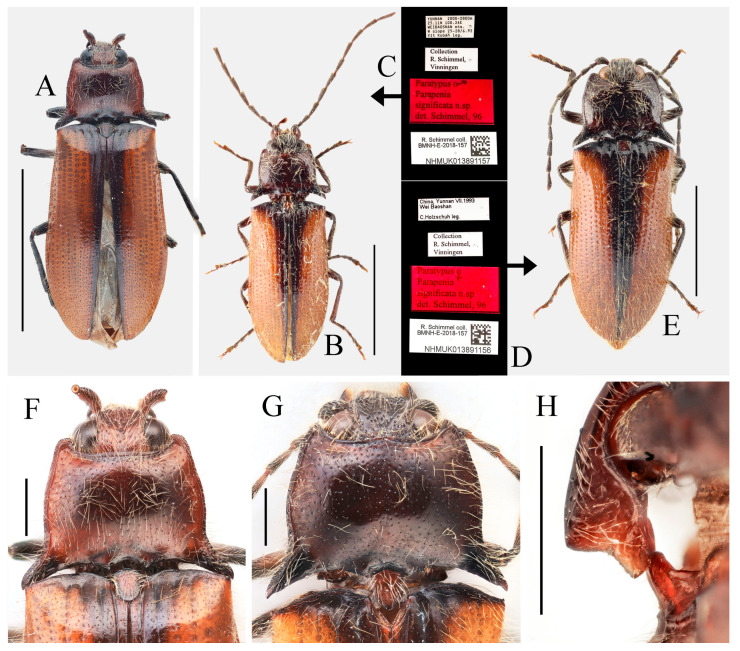
*Sinopenia* **gen. nov.** and *S. significata* (Schimmel, 1998) **comb. nov.** (**A**) Male from Yunnan, Yingjiang, dorsal view; (**B**,**C**) holotype, male (dorsal view) and its labels; (**D**,**E**) paratype, female (dorsal view) and its labels; (**F**) head and pronotum, male from Yingjiang, dorsal view; (**G**) head and pronotum, female from Yunnan, Dali, Yongping, dorsal view; (**H**) prosternal process, male from Yingjiang, lateral view. (**B**–**E**) photographed by Keita Matsumoto, copyright BMNH. Scale bars 5 mm for (**A**,**B**,**E**); 1 mm for (**F**–**H**).

**Figure 29:**
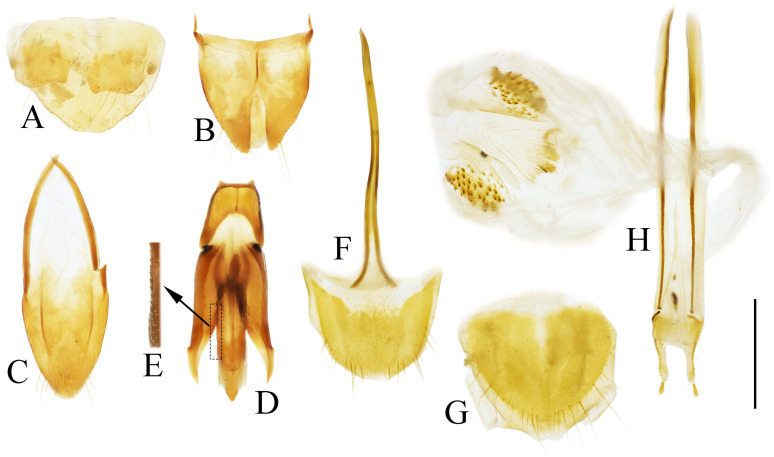
Pregenital segments and genitalia of *Sinopenia significata* (Schimmel, 1998) comb. nov., male from Yunnan, Yingjiang (**A**–**E**) and female from Yunnan, Dali, Yongping (**F**–**H**). (**A**) Sternite VIII and tergite VIII, ventral view; (**B**) tergites IX–X, dorsal view; (**C**) sternite IX, dorsal view; (**D**) aedeagus, ventral view; (**E**) micro serration of median lobe at lateral margins; (**F**) sternite VIII, ventral view; (**G**) tergite VIII, dorsal view; (**H**) ovipositor and genital tract, ventral view. Scale bar 1 mm, (**E**) not to scale.

ZooBank LSID: urn:lsid:zoobank.org:act:19F4D894-0F36-48E5-9DBC-61365BFE0752
Figure 25G–I, Figure 28A–H, Figure 29A–H and Figure 40

**Type species.** *Parapenia significata* Schimmel, 1998; here designated.

**Chinese common name.** 华薄叩甲属

**Diagnosis.** Antennomeres elongate filiform; antennomere II shortest, distinctly shorter than III. Pronotum with quadrate anterior angles, each bearing one shallow, meniscate depression. Posterior angles projecting laterally, indistinctly bidentate at apex, with one small, indistinct notch near each posterior margin of posterior angle. Prosternal process carinated laterally, media concaved, apical portion with short carination medially; widened in lateral view, apex with shallow but wide notch. Elytral anterior angles bidentate. Tarsomeres III–IV lobed. Aedeagus with short median lobe and parameres; parameres tapering apically, each with a distinct subapical hook. Ovipositor elongate. Bursa copulatrix bearing a large sclerotized region composed of microspines, differentiated into three distinct zones: two large lateral zones with heavily sclerotized spines, and one small basal zone with finer spines.

This genus resembles *Parapenia* in having bidentate posterior angles of pronotum and bidentate anterior angles of elytra, but can be readily distinguished by the shape of the pronotal anterior angles, the structure of the aedeagus, and the pattern of sclerotized structures in the bursa copulatrix. Both *Sinopenia* **gen. nov.** and *Parapenioides* **gen. nov.** have weakly bidentate posterior angles of the pronotum, but differ from each other in the shape of the pronotal anterior angles, the aedeagus, and the structure of the sclerotized plates of the bursa copulatrix. See Table 1 for detailed comparisons among these genera.

**Etymology.** The generic name is a combination of “Sino” (Chinese) and “*Penia*” (a genus in Dimini). Gender: feminine.

**Distribution.** China (Figure 40).

***Sinopenia significata* (Schimmel, 1998) comb. nov.**
Figure 28A–H, Figure 29A–H and Figure 40

*Parapenia significata* Schimmel, 1998: 103 [9] (original description); Cate 2007: 184 [29] (catalogue); Kundrata et al. 2018: 33 [3] (catalogue).

**Chinese common name.** 壮丽华薄叩甲

**Type locality.** China: Yunnan: Dali Bai Autonomous Prefecture, Weishan County, West slope of Weibaoshan Mountain, 2000–2800 m.

**Type material examined. Paratypes** of *Parapenia significata* Schimmel, 1998: 1 male (NHMUK), “Paratypus [print] ♂/Parapenia [print]/significata n. sp. [print]/det. Schimmel, 96 [print]”, “YUNNAN 2000–2800 m/25.11 N 100.24 E/WEIBAOSHAN mts./W slope 25–28/6.92/Vít Kubáň leg.” [print], “Collection/R. Schimmel,/Vinningen” [print], “R. Schimmel coll./BMNH-E-2018-157/NHMUK013891157” [print, with a QR code]; 1 female (NHMUK), “Paratypus [print] ♀/Parapenia [print]/significata n. sp. [print]/det. Schimmel, 96 [print]”, “China, Yunnan VII.1993/Wei Baoshan/C.Holzschuh leg.” [print], “Collection/R. Schimmel,/Vinningen” [print], “R. Schimmel coll./BMNH-E-2018-157/NHMUK013891156” [print, with a QR code].

**New material examined.** 1 male (MYTC), Bangba Forest Farm [邦巴林场], Yingjiang County, Dehong Dai and Jingpo Autonomous Prefecture, Yunnan Province, China, VII. 2019, local leg.; 1 female (DLU), Jinguangsi Temple [金光寺], Yongping County [永平县], Dali Bai Autonomous Prefecture, Yunnan Province, China, 28.VII.2019, Ji-Shan Xu leg.

**Diagnosis.** Body length 10.9–12.5 mm; width 3.1–4.2 mm; antenna length 7.9 mm (male) and 6.8 mm (female); pronotum length × width = 1.9–2.2 × 3.7–4.2 mm; elytra length 7.7–9.1 mm. Body elongate (elytra/pronotum length ratio = 4.0–4.1:1), bicolored: head, pronotum, scutellar shield, prosternum, hypomeron, meso- and metaventrite, abdomen, and legs reddish brown to black; elytra yellow, with basal region and sutures brown to blackish (Figure 28A–E). Males usually exhibit a paler coloration than females. Pubescence yellow. Head with a distinct V-shaped depression. Antennae reaching beyond the middle of elytra in males, and merely reaching the middle of elytra in females. Pronotum subtrapezoidal (Figure 28F,G). Anterior angles stout; lateral margins slightly arched. Posterior angles narrowed with sharp apices. Pronotal punctures small; interspaces between punctures wide, approximately 3–5 puncture diameters. Prosternal process laterally widened, apical portion with a broad notch, shaped as in Figure 28H. Elytral length/width ratio = 2.2–2.5:1. Elytral apices jointly rounded. Abdominal ventrite V rounded apically.

Male: tergite VIII semi-oval (Figure 29A). Sternite VIII with two darkened areas; inner sides of distal end protruded (Figure 29A). Tergite IX semi-oval, apices rounded (Figure 29B). Sternite IX elongate, oval, 2.8 times longer than wide (Figure 29C). Aedeagus stout; median lobe short, with subparallel sides, microspines present laterally (Figure 29E), apex blunt and slightly extending beyond the apices of parameres. Parameres stout, apex abruptly narrowed, with a small subapical hook (Figure 29D).

Female: slightly larger than males (Figure 28E), tergite VIII semi-oval, apex blunt (Figure 29G). Sternite VIII semi-oval; spiculum ventrale 2.7 times longer than sternite VIII (Figure 29F). Bursa copulatrix with a nearly membranous sclerotized plate; surface slightly wrinkled and densely covered with microspines; two dark-colored lateral zones with large spines, basal zone thickened (Figure 29H).

**Distribution.** China: Yunnan (Figure 40).

**Bionomics**. Unknown.

**Remarks.** Schimmel [9] noted this species as striking within *Parapenia* due to its coloration and aedeagal structure. However, based on examination of the type photos and newly collected material, we found that this species differs significantly from *Parapenia* by the absence of the narrow projection of anterior angle of pronotum, and the presence of the stout aedeagus and large, membrane-like sclerotized region in the bursa copulatrix. These diagnostic characters together justify the removal of this species from *Parapenia* and warrant the establishment of a new genus.

#### 3.2.3. *Megapenia* gen. nov.

ZooBank LSID: urn:lsid:zoobank.org:act:A2A6DFA2-9E3C-4262-893D-7A971D88C228
Figure 25J–L, Figure 30A–F, Figure 31A–D, Figure 32A–F, Figure 33A–E, Figure 34A–H, Figure 35A–C, Figure 36A–D, Figure 37A–H and Figure 40

**Type species:***Penia marginalis* Fleutiaux, 1942, here designated.

**Chinese common name**. 硕薄叩甲属

**Diagnosis.** Antennomeres elongate filiform; antennomere II shortest, distinctly shorter than III. Pronotum with anterior angles laterally rounded, each bearing a shallow impression. Posterior angles elongate, extending posterolaterally before curving to point directly posteriorly; apices of posterior angles with a cluster of erect, elongate setae along the lateral margins (Figure 25K). Prosternal process with median carina (Figure 31B, Figure 33D and Figure 36B), narrowed in lateral view, apex tapered. Elytra with anterior angles carinate; base laterally weakly concave to accommodate the posterior angles of the pronotum. Tarsomeres III–IV lobed. Tarsomere IV with conspicuously long bilateral setal tufts whose curved distal parts contact beneath tarsomere V, forming a visible annulus. Aedeagus with variable shapes of the median lobe and parameres. Female with shortened spiculum ventrale and ovipositor. Bursa copulatrix with three sclerotized plates, two small plates overlapping one large plate (except in *M. tianlongi* **sp. nov.**, in which the sclerotized plates are reduced to small membranous structures bearing several spines); the large plate typically T-shaped, the smaller ones band-like.

**Etymology.** The generic name refers to the relatively large body size of the genus. Gender: feminine.

**Distribution.** China, Myanmar, Thailand, Vietnam (Figure 40).

**Figure 30:**
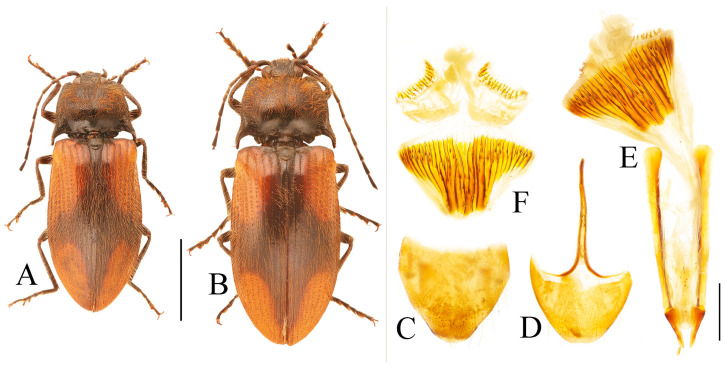
*Megapenia* **gen. nov.** and *M. cruciata* (Bouwer, 1991) **comb. nov.** (**A**) Female from Yunnan, Dulongjiang, dorsal view; (**B**–**F**) female from Yunnan, Yingjiang: (**B**) habitus, dorsal view; (**C**) tergite VIII, dorsal view; (**D**) sternite VIII, ventral view; (**E**) ovipositor and genital tract, ventral view; (**F**) sclerotized part of bursa copulatrix. Scale bars 5 mm for (**A**,**B**); 1 mm for (**C**–**F**).

**Remarks.** This genus closely resembles *Penia* in overall morphology, but can be readily distinguished by the presence of large sclerotized plates in the bursa copulatrix (Figure 30F and Figure 34H), a feature absent in *Penia* and many other genera within Dimini (e.g., *Dima*, *Sinodima*, *Neodima*, and *Neocsikia*) [13,14,15,16,19,20,21,22]. Although some species of *Penia* and *Dima* possess bursal sclerotizations (e.g., *Penia takasago* Kishii, 1997 and *Dima* species from the Balkan Peninsula), these are exclusively composed of spinose elements rather than large, plate-like structures [13,45,46]. Compared to the aforementioned genera, the bursa copulatrix of *Platiana* bears a circular sclerotized structure with a central or subcentral long spine, differing from that of *Megapenia* [47].

This genus exhibits relatively consistent interspecific morphology, particularly in the shape of the pronotum and the presence of long setae or pubescence on the posterior angles of the pronotum and on the fourth tarsomeres. Although the male genitalia vary notably among species (Figure 34D and Figure 37E), females consistently possess a short spiculum ventrale (1.4–2.8 times of sternite VIII length) and ovipositor (Paraproct/coxite length ratio 2.2–4.0:1), both comparatively shorter than those found in *Parapenia*, *Parapenioides*, and *Sinopenia* (e.g., spiculum ventrale more than 2.7 times of sternite VIII length; paraproct/coxite length ratio 4.6–5.8:1). *Megapenia cruciata* (Bouwer, 1991) **comb. nov.** and *M. marginalis* (Fleutiaux, 1942) **comb. nov.** share similar bursa copulatrix structures, each with one large and two small sclerotized plates. *M. tianlongi* **sp. nov.** is unique in having a markedly reduced sclerotized portion (Figure 37F), yet its inclusion in this genus is unambiguous based on other diagnostic characters as noted above.

This genus is further distinguished by its relatively large body size compared to other Dimini genera. All known species can exceed 15 mm in body length. Notably, the female paratype of *Megapenia tianlongi* **sp. nov.** reaches 19.1 mm, which may represent the largest known individual in Dimini. While other genera do include exceptionally large species, such as *Dima bruhai* Mertlik, Németh & Kundrata, 2017 (17.1 mm) and *Penia pendleburyi* Fleutiaux, 1934 (16.0 mm) [2,45], these are outliers; most species in both *Dima* and *Penia* rarely exceed 15 mm, and many are substantially smaller (often under 10 mm).

**Key to species of *Megapenia***

1. Elytra with four large yellow maculae laterally, two at basal and two at apical portions……………………………………………………………………‥‥ *M. cruciata* (Bouwer, 1991) **comb. nov.**
- Elytra lacking maculae but exhibiting bicolored pattern between median and lateral areas…………………………………………….……………………………………………………. 2
2. Elytra with sharply demarcated black median stripe and yellow lateral fields; aedeagus with median lobe gradually tapered; bursa copulatrix with large, well-developed sclerotized plates…………………………………….……‥ *M. marginalis* (Fleutiaux, 1942) **comb. nov.**
- Elytra with gradual color transition from brown median area to yellowish-brown laterally; apical portion of median lobe arrowhead-shaped; bursa copulatrix with sclerotized plates highly reduced, retaining only minute and scattered spines………………………………………………………………………‥ *M. tianlongi* **sp. nov.**

**Figure 31:**
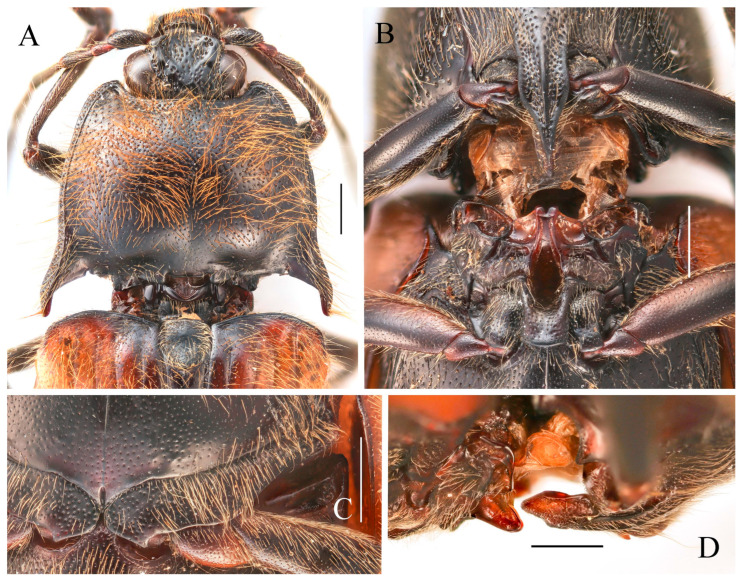
Characters of *Megapenia cruciata* (Bouwer, 1991) **comb. nov.**, female from Yunnan, Yingjiang. (**A**) Head and pronotum, dorsal view; (**B**) posterior part of prothorax and mesothorax, ventral view; (**C**) metacoxal plate, ventral view; (**D**) prosternal process, lateral view. Scale bar 1 mm.

***Megapenia cruciata* (Bouwer, 1991) comb. nov.**
Figure 1H, Figure 30A–F, Figure 31A–D and Figure 40

*Penia cruciata* Bouwer, 1991: 234 [42] (original description); Schimmel 1996: 168 [2] (remark); Kundrata et al. 2018: 40 [3] (catalogue).

**Chinese common name.** 四斑硕薄叩甲

**Type locality.** N Thailand: Chiang Mai: Doi Inthanon.

**New material examined.** 1 female (MYTC), Qinlangdang, Dulongjiang Township, Gongshan County, Nujiang Lisu Autonomous Prefecture, Yunnan Province, China, 1350 m, 4–7.IX.2023, Chao Wu leg.; 2 females (MYTC), Yingjiang County, Dehong Dai and Jingpo Autonomous Prefecture, Yunnan Province, China, VII–VIII.2019, local leg.; 1 female (MYTC), Xima Town, Yingjiang County, Dehong Dai and Jingpo Autonomous Prefecture, Yunnan Province, China, VI–VII.2018, Wei-Zong Yang leg.

**Diagnosis.** Male body length 16.0 mm, width 6.0 mm [42]; female body length 14.2–16.4 mm, width 5.7–6.3 mm, antenna length 9.0–9.1 mm, pronotum length × width = 2.8–3.3 × 5.2–5.9 mm, elytra length 10.4–11.5 mm. Body elongate (Elytra/pronotum length ratio = 3.5–3.7/1), dark brown to black; trochanters and antennomere 2 reddish brown; elytra bicolor, dark brown to black with paired large yellow maculae laterally on basal and apical portions. Pubescence yellow (Figure 30A,B). Head with central depression. Antenna beyond posterior angle of pronotum about apical three antennomeres, not reaching elytral mid-length. Pronotum with round, deep punctures, intervals between punctures about 2–3 puncture diameter, punctures smaller and sparser posteriorly (Figure 31A). Elytra widest at distal half, apices each rounded.

All our Chinese specimens are females. While lacking the aedeagus, they otherwise completely match the original description and illustrations. The sole discrepancy lies in the elytral basal spots, our specimens show larger spots compared to the smaller ones depicted in the original illustration, though this character proves variable in our material. Notably, Bouwer’s [42] illustration correctly depicts the curved elongate setae on the fourth tarsomere, which is one of the diagnostic characters for genus *Megapenia*.

**Figure 32:**
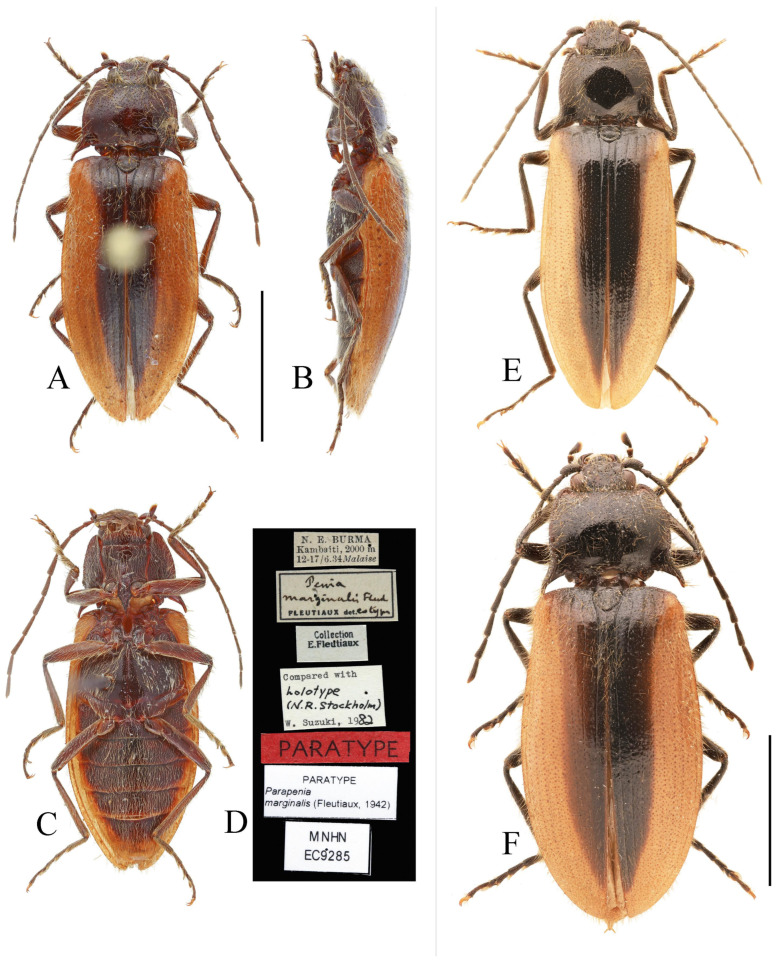
*Megapenia* **gen. nov.** and *M. marginalis* (Fleutiaux, 1942) **comb. nov.** (**A**–**D**) Paratype of *Penia marginalis* Fleutiaux, 1942, sex undetermined (probably male) and its labels; (**A**) dorsal view; (**B**) lateral view; (**C**) ventral view; (**D**) labels; (**E**) male from Yunnan, Fugong, Shiyueliang, dorsal view; (**F**) female from the same locality as (**E**), dorsal view. (**A**–**E**) photographed by Christophe Rivier, copyright MNHN. Scale bar 5 mm.

**Figure 33:**
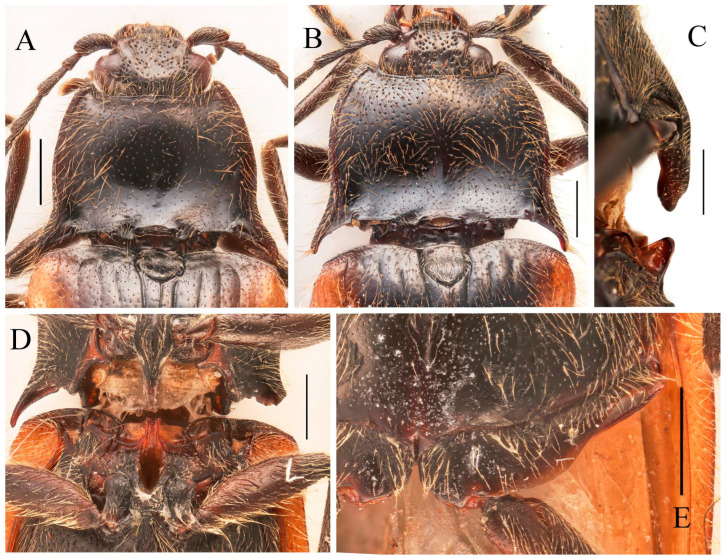
Characters of *Megapenia marginalis* (Fleutiaux, 1942) **comb. nov.**, male from Yunnan, Fugong, Shiyueliang and female from Yunnan, Yingjiang, Laomahe. (**A**) Head and pronotum, male, dorsal view; (**B**) head and pronotum, female, dorsal view; (**C**) prosternal process, female, lateral view; (**D**) posterior part of prothorax and mesothorax, female, ventral view; (**E**) metacoxal plate, female, ventral view. Scale bar 1 mm.

**Figure 34:**
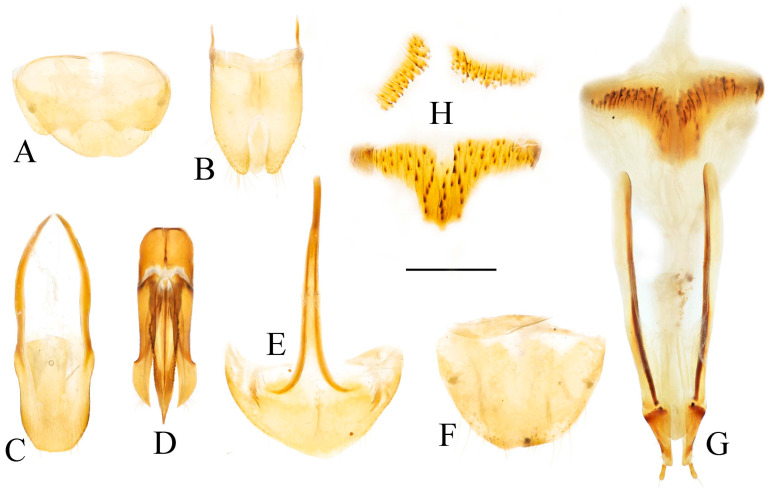
Pregenital segments and genitalia of *Megapenia marginalis* (Fleutiaux, 1942) **comb. nov.**, male from Yunnan, Fugong, Shiyueliang (**A**–**D**) and female from Yunnan, Yingjiang, Laomahe (**E**–**H**). (**A**) Sternite VIII and tergite VIII, ventral view; (**B**) tergites IX–X, dorsal view; (**C**) sternite IX, dorsal view; (**D**) aedeagus, ventral view; (**E**) sternite VIII, ventral view; (**F**) tergite VIII, dorsal view; (**G**) ovipositor and genital tract, ventral view; (**H**) sclerotized part of bursa copulatrix. Scale bar 1 mm.

***Megapenia marginalis* (Fleutiaux, 1942) comb. nov.**
Figure 1G, Figure 32A–F, Figure 33A–E, Figure 34A–H and Figure 40

*Penia marginalis* Fleutiaux, 1942: 17 [48] (original description); Bouwer 1991: 237 [42] (catalogue); Schimmel 1996: 177 [2] (diagnosis).

*Parapenia marginalis* (Fleutiaux, 1942): Schimmel 1996: 169 [12] (new combination); Kundrata et al. 2018: 32 [3] (catalogue).

**Chinese common name.** 黄边硕薄叩甲

**Type locality.** N. E. Myanmar: Kachin: Kambaiti, 2000 m.

**Type material examined. Paratype** of *Penia marginalis* Fleutiaux, 1942: sex undetermined (probably male) (MNHN), “N. E. BURMA/Kambaiti, 2000 m/12–17/6.34 *Malaise*” [print], “Collection/E.Fleutiaux” [print], “Penia [handwriting]/marginalis Fleut. [handwriting]/Fleutiaux det. [print] cotype [handwriting]”, “PARATYPE” [print in red background], “Compared with [print]/holotype [handwriting]/(N.R. Stockholm) [handwriting]/W. Suzuki, 19 [print] 82 [handwriting]”, “PARATYPE/*Parapenia*/*marginalis* (Fleutiaux, 1942)” [print], “MNHN/EC9285” [print].

**New material examined.** 3 females (MYTC), Laomahe [老马河], Sudian Township [苏典乡], Yingjiang County, Dehong Dai and Jingpo Autonomous Prefecture, Yunnan Province, China, 2200 m, 16.VIII.2018, Zi-Chun Xiong leg.; 1 male and 2 females (MYTC), Shiyueliang Township [石月亮乡], Fugong County [福贡县], Nujiang Lisu Autonomous Prefecture, Yunnan Province, China, 2400–2700 m, VI.2022, local leg.; 1 female (MYTC), Pianma Town [片马镇], Lushui City [泸水市], Nujiang Lisu Autonomous Prefecture, Yunnan Province, China, 2300 m, 15.IX.2023, Chao Wu leg.

**Diagnosis.** Female slightly larger than male, body length 11.3–15.2 mm (Schimmel [2] recorded the paratype of *Penia marginalis* from MNHN as 9 mm, however, it is actually about 11.3 mm in length), width 4.5–6.0 mm, antenna length 7.2–8.1 mm, pronotum length× width = 2.1–2.7 × 3.8–4.8 mm, elytra length 9.4–10.8 mm. Body elongate (Elytra/pronotum length ratio = 3.8:1 in male, 3.8–4.1:1 in females), dark brown to black, legs reddish brown, abdomen dark brown, with lateral and apical portions reddish brown, elytra bicolor, middle part (between striae 4 of each side) dark brown to black, the dark portion gradually narrowed from base to apices, rest portions yellow (Figure 32E,F). Pubescence yellow. Head depressed. Antennae not reaching middle of elytra. Pronotum subtrapezoidal (Figure 33A,B). Anterior angle of pronotum stout, apex rounded. Lateral margins of pronotum slightly arched. Posterior angle of pronotum elongate, narrow, tapered, curved and pointing backward. Punctures small, intervals between punctures of pronotum spaced, approximately 4–6 puncture diameters. Prosternal process laterally narrowed, ventral-apical portion with shallow notch (Figure 33C). Elytra widest at distal half; striae well delimited by punctures; apices each rounded. Male: abdominal ventrite V truncated apically. Tergite VIII semioval, with emargination apically (Figure 34A). Sternite VIII with two dark portions, each with two protrusions distally (Figure 34A). Sternite IX elongate, apex somewhat truncate, 3.0 times longer than wide (Figure 34C). Aedeagus stout, median lobe tapered, with microspines at sides, apex beyond apices of parameres. Paramere stout, apical portion enlarged, apex sharp, lateral margin convex (Figure 34D).

Female: abdominal tergite VIII semioval (Figure 34F); sternite VIII semi-oval, spiculum ventrale 2.8 times longer than sternite VIII length (Figure 34E). Paraproct/coxite length ratio = 4.0/1; Bursa copulatrix with three sclerotized plates, large one T shaped, two small ones band-like, all plates with sparse spines (Figure 34G,H).

**Distribution.** China: Yunnan; Myanmar; Vietnam (Schimmel [2] recorded it from Vietnam, but with no further information) (Figure 40).

**Bionomics.** Adult can be found at daytime (Chao Wu, personal observation of a single specimen).

**Remarks.** This species was originally described as *Penia* [48]. Later, based solely on the presence of erect pubescence, Schimmel [2] transferred it to *Parapenia*, considering that the bidentate posterior angles of *Penia marginalis* were rudimentarily present and should be regarded as a case of reduction. However, based on newly examined material and type photographs, we found that the pronotum of *P. marginalis* is distinct from that of *Parapenia*, lacking the narrow protrusion at anterior angles and the bidentate posterior angles. Additionally, the anterior angles of the elytra are not bidentate, and both the aedeagus and the large sclerotized plates of the bursa copulatrix markedly differ from those of *Parapenia*. Consequently, we confirm that this species does not belong to *Parapenia*, nor can it be transferred back to *Penia*, as species of *Penia* are known to lack large sclerotized plates in the bursa copulatrix [13,15]. Unique characters of bursa copulatrix sclerotization and relatively large body size warrant its placement in a new genus.

**Figure 35:**
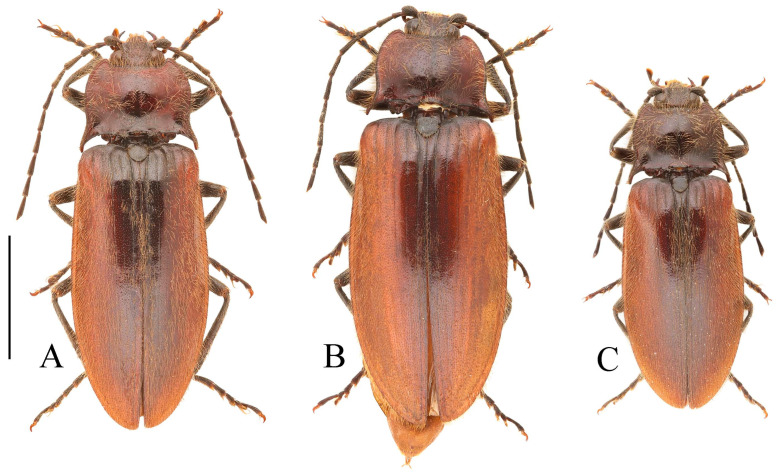
*Megapenia tianlongi* gen. & sp. nov. from Yunnan, Daweishan Mountain, dorsal view. (**A**) Male; (**B**,**C**) female. Scale bar 5 mm.

**Figure 36:**
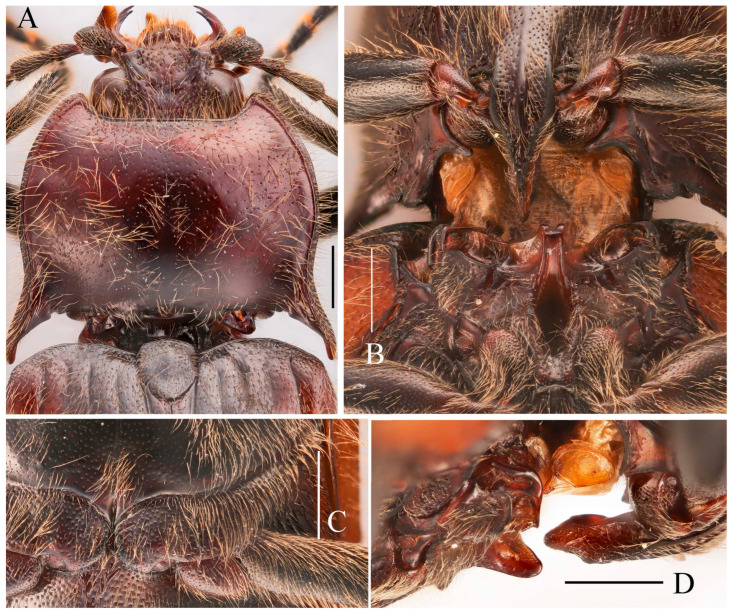
Characters of *Megapenia tianlongi* gen. & sp. nov., male from Yunnan, Daweishan Mountain. (**A**) Head and pronotum, dorsal view; (**B**) posterior part of prothorax and mesothorax, ventral view; (**C**) metacoxal plate, ventral view; (**D**) prosternal process, lateral view. Scale bar 1 mm.

**Figure 37:**
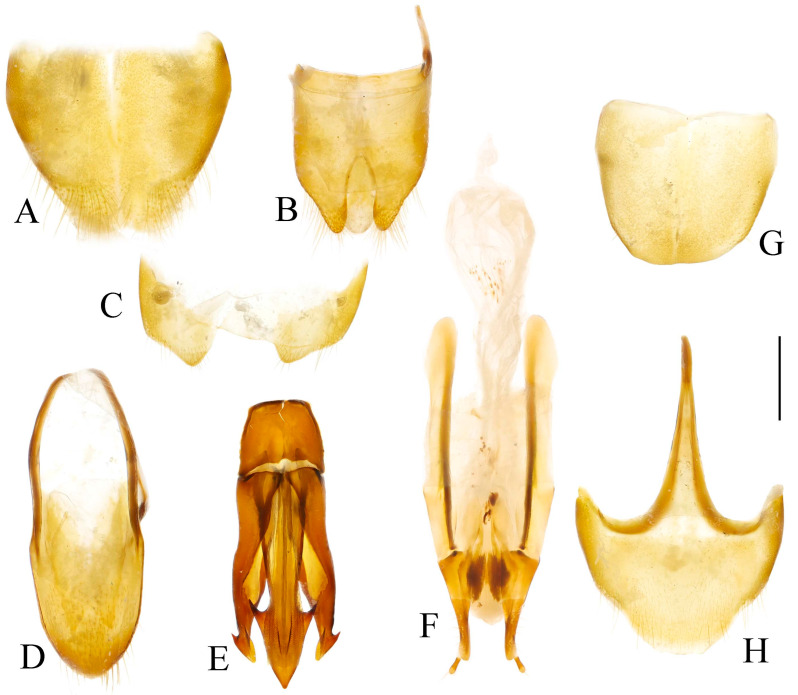
Pregenital segments and genitalia of *Megapenia tianlongi* gen. & sp. nov., male (**A**–**E**) and female (**F**–**H**) from Yunnan, Daweishan Mountain. (**A**) Tergite VIII, dorsal view; (**B**) tergites IX–X, dorsal view; (**C**) sternite VIII, ventral view; (**D**) sternite IX, dorsal view; (**E**) aedeagus, ventral view; (**F**) ovipositor and genital tract, ventral view; (**G**) tergite VIII, dorsal view; (**H**) sternite VIII, ventral view. Scale bar 1 mm.

***Megapenia tianlongi* sp. nov.**
ZooBank LSID: urn:lsid:zoobank.org:act:1701B6BC-98AC-42BE-B84B-87CEED3C4564
Figure 35A–C, Figure 36A–D, Figure 37A–H and Figure 40

**Chinese common name.** 天龙硕薄叩甲

**Type locality.** China: Yunnan: Honghe Hani and Yi Autonomous Prefecture, Pingbian County, Mt. Daweishan, 2050 m.

**Type material. Holotype:** male (MYTC), Mt. Daweishan, Pingbian County, Honghe Hani and Yi Autonomous Prefecture, Yunnan Province, China, 2050 m, 1–2.VI.2022, Tian-Long He leg. **Paratype:** 2 specimens: 1 female (MYTC), same data as holotype; 1 female (MYTC), same locality as holotype, but 1900–2080 m, E 103°41′53″, N 22°54′16″, 18.V.2016, Lu Qiu leg.

**Diagnosis.** Body length 15.3–19.1 mm. Body bicolored, dorsal part reddish brown; legs, antennae, and ventral surface dark brown; elytra gradually paler from dark brown medially to reddish brown laterally. Antennae extend beyond posterior angles of pronotum by approximately four apical antennomeres, reaching slightly before mid-length of elytra. Pronotum with small and sparse punctures, interspaces flat and smooth, approximately 4–7 puncture diameters. Elytra 4.0–4.5 times longer than pronotum length, widest at distal half. Striae 1–5 mainly formed by grooved lines rather than punctures, lines mostly shallow except basal portion. Apices together rounded. Median lobe with apical portion arrowhead-shaped. Bursa copulatrix with sclerotized part highly reduced, containing a group of sparse spines.

**Description.** Holotype, male: body length 18.3 mm; width 6.4 mm; antenna length 9.7 mm; pronotum length × width = 3.2 × 5.5 mm; elytra length 13.4 mm.

Body elongate (elytra/pronotum length ratio = 4.0:1), bicolored, elongate, densely covered with long pubescence. Dorsal part reddish brown; legs, antennae, and ventral surface dark brown; elytra gradually paler from dark brown medially to reddish brown laterally. Pubescence brownish yellow (Figure 35A).

Head smooth, with broad median depression. Anterior margin of frons protruding, abruptly declined medially. Frons with small punctures; interspaces 2–4 puncture diameters. Antennae extend beyond posterior angles of pronotum by approximately four apical antennomeres, reaching slightly before mid-length of elytra. Ratio of antennomeres II to III = 1:1.5 (Figure 36A).

Pronotum broadly rounded. Anterior margin straight; anterior angles with outer margin arched and inner margin truncate. Lateral margins evenly arched; posterior angles elongate, directed posteriorly. Surface smooth, with small and sparse punctures; interspaces flat and smooth, 4–7 puncture diameters (Figure 36A).

Chin-piece with umbilicate punctures, interspaces subequal to or less than one puncture diameter, sparser medially. Median part of prosternum smooth and flat, with punctures smaller than those on chin-piece; interspaces about 3 puncture diameters. Prosternum with puncture size and spacing similar to pronotum. Median carina shallow but broad. Prosternal process with apex narrowed and slightly notched ventrally (Figure 36D). Hypomeron with dense oval punctures; punctation similar to that of prosternum. Metaventrite with fine punctures and smooth interspaces, separated by 1–2 puncture diameters. Abdominal surface with coarser punctures than on metaventrite; interspaces similar but slightly wrinkled. Elytral length/width ratio = 2.0:1; Striae 1–5 deeply grooved at base, becoming shallower toward apex; striae 6–9 represented by rows of punctures. Interstriae flat and smooth, with fine punctures spaced 4–6 puncture diameters. Elytral apices each rounded, without spines.

Apex of ventrite V rounded. Tergite VIII subtrapezoidal, with truncate apical margin and shallow median concavity (Figure 37A). Sternite VIII with two darkened areas and slight protrusions on the inner apical margin (Figure 37C). Tergite IX with sides straight and subparallel in basal half, abruptly narrowed distally; narrowed portion with straight sides and rounded apices (Figure 37B). Tergite X with rounded apex. Sternite IX elongate, apex rounded, 2.7 times longer than wide (Figure 37D).

Median lobe narrowing from base to apex; apical portion modified, arrowheaded with pointed tip and two prominent lateral hooks. Parameres with undulate lateral margins; apices curved inward and tapered, with sharp subapical hooks. Phallobase subtrapezoidal (Figure 37E).

Paratype females: body length 15.3–19.1 mm, elytra/pronotum length ratio = 4.0–4.5:1. No significant difference compared to male (Figure 35B,C). Tergite VIII semioval, apex blunt (Figure 37G); sternite VIII semioval, apical portion slightly protruded, rounded, spiculum ventrale 1.4 times longer than sternite VIII length (Figure 37H). Ovipositor short, paraproct/coxite length ratio = 2.2:1; coxite gradually tapered, apices slightly widened; styli distinct, cylindrical, inserted subapically. Bursa copulatrix with sclerotized part highly reduced, containing a group of sparse spines (Figure 37F).

**Distribution.** China: Yunnan (Figure 40).

**Bionomics.** Unknown.

**Etymology.** Named after Mr. Tian-Long He [贺天龙] (Anhui, China), collector of the holotype of the new species.

**Remarks.** The large body size, the curved and elongate posterior angles of the pronotum bearing tufts of long setae, and the distinct pair of curved long setae on both sides of the fourth tarsomere, short ovipositor strongly support the placement of this new species within the genus *Megapenia*. Interestingly, this species exhibits a notable reduction in sclerotized structures in the bursa copulatrix, which is only weakly sclerotized and bears sparse, small spines. Nevertheless, the weakly sclerotized area remains pleated, resembling the characteristic pleated sclerotized plates found in its congeners. Despite the reduction in the internal sclerotization, the external morphological characters are consistent with those of *Megapenia*, justifying its generic assignment.

### 3.3. Checklist of Known Species of Parapenia, Parapenioides, Megapenia, and Sinopenia

***Parapenia* Suzuki, 1982:**

*Parapenia assamensis* Suzuki, 1982 (India)
*Parapenia fuxi* **sp. nov.** (China)
*Parapenia nigroapicalis* Suzuki, 1982 (Thailand)
*Parapenia nyuwa* **sp. nov.** (China)
*Parapenia pangu* **sp. nov.** (China)
*Parapenia rugosicollis* Schimmel, 2001 (India)
*Parapenia ruihangi* **sp. nov.** (China)
*Parapenia sausai* Schimmel, 1998 (China, India)
*Parapenia spicula* Schimmel, 2001 (Laos)
*Parapenia taiwana* (Miwa, 1930) (China)
*Parapenia thailandica* Suzuki, 1982 (Thailand)
*Parapenia tonkinensis* (Fleutiaux, 1918) (China, Vietnam)
*Parapenia villosa* (Fleutiaux, 1936) (China, Vietnam)
*Parapenia wuchaoi* **sp. nov.** (China)
*Parapenia wulingshanensis* Schimmel, 2006 (China)
*Parapenia yunnana* Schimmel, 1993 [=*Parapenia jagemanni* Schimmel, 2001 **syn. nov.**] (China)
*Parapenia zhengi* **sp. nov.** (China)

  ***Parapenioides*** **gen. nov.:**

*Parapenioides zidani* **sp. nov.** (China)

  ***Sinopenia* gen. nov.:**

*Sinopenia significata* (Schimmel, 1998) **comb. nov.** (China)

  ***Megapenia* gen. nov.:**

*Megapenia cruciata* (Bouwer, 1991) **comb. nov.** (China, Thailand)
*Megapenia marginalis* (Fleutiaux, 1942) **comb. nov.** (China, Myanmar, Vietnam)
*Megapenia tianlongi* **sp. nov.** (China)

**Figure 38:**
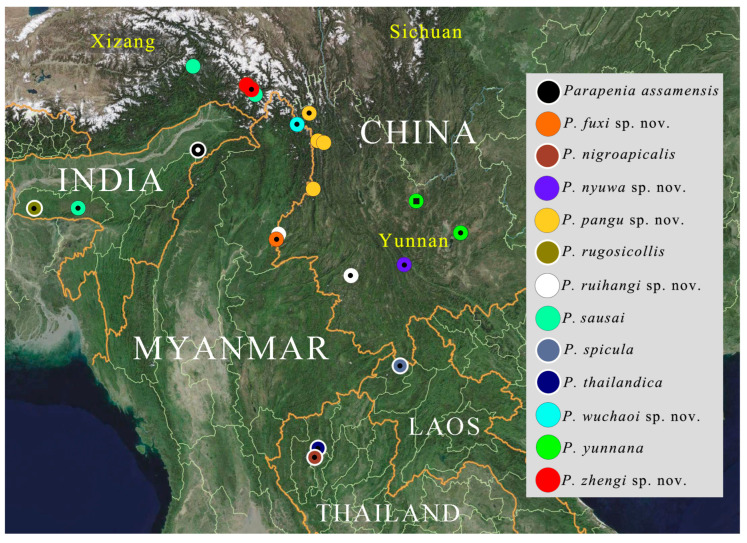
Distribution of *Parapenia* species in China (Yunnan, Xizang), India, Laos, and Thailand. Type localities are marked by a black dot at the center of each circle (black square for *P. jagemanni* Schimmel, 2001 **syn. nov.**; white dot for *P. assamensis*). White-bordered circles denote species recorded outside China.

**Figure 39:**
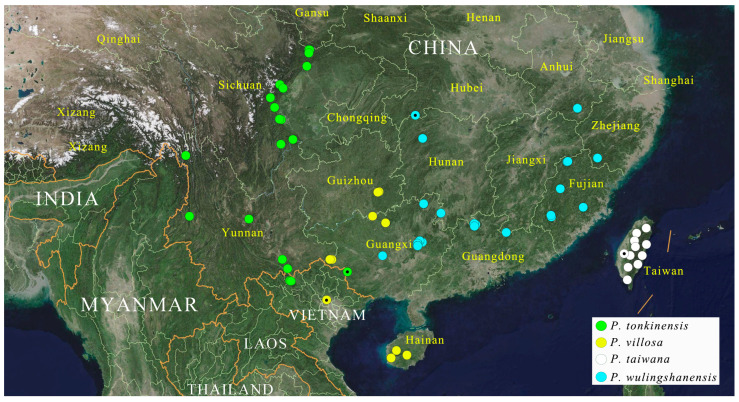
Distribution of *Parapenia tonkinensis*, *P. villosa*, *P. taiwana*, and *P. wulingshanensis*. Type localities are marked by a black dot at the center of each circle.

**Figure 40:**
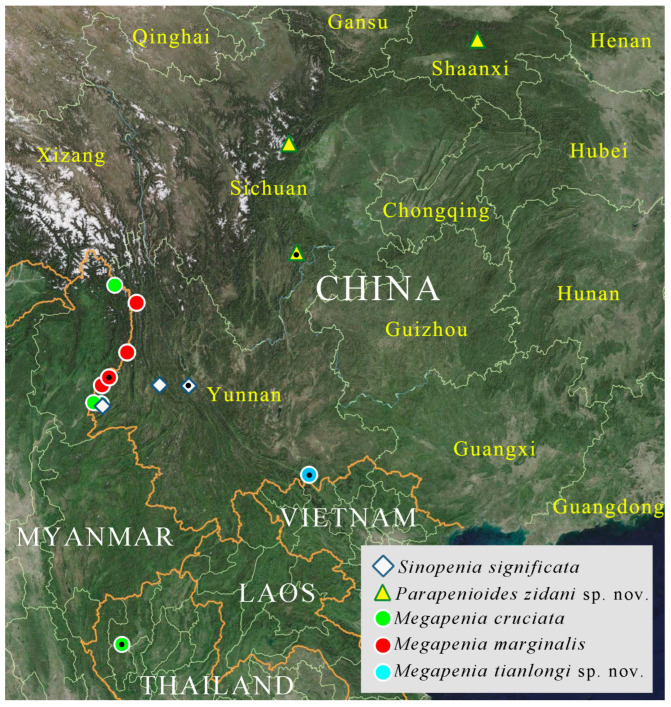
Distribution of *Sinopenia* **gen. nov.**, *Parapenioides* **gen. nov.**, and *Megapenia* **gen. nov.** Type localities are indicated by a black dot at the center of each symbol.

## 4. Discussion

### 4.1. Taxonomic Highlights of the Study on Parapenia and the New Genera

Beyond the routine tasks of reviewing and describing species, we emphasize the diagnostic significance of bursa copulatrix sclerites in *Parapenia* and other genera of Dimini. Sclerotized structures in the bursa copulatrix are relatively common in Elateridae and have traditionally served as useful characters for distinguishing species or genera [49,50,51,52]. However, their occurrence within Dimini appears to be more limited.

Previous studies have shown that bursa copulatrix sclerites are absent in small genera such as *Sinodima*, *Neodima*, and *Neocsikia* [19,20,22]. In the larger genera *Dima* and *Penia*, most species have not been examined for female genital structures, but current evidence suggests a general absence of sclerotizations. In *Dima*, sclerotized elements have been identified primarily in European species, typically as small spiniform structures [45,46], whereas all examined Asian species lack such features [23,24,25]. Similarly, in *Penia*, most species lack bursal sclerites, though exceptions exist, for instance, *P. takasago* possesses small internal spines [13]. Prior to this study, *Platiana* was the only genus in Dimini known to have distinct sclerotizations, represented by a circular plate with a centrally positioned long spine [47].

In contrast to the aforementioned genera, *Parapenia* consistently exhibits strongly developed and morphologically uniform sclerotized structures in the bursa copulatrix across species. These structures are more complex and clearly distinct from those of *Platiana*. Although they may not offer sufficient variation for species-level diagnosis, their structural consistency renders them valuable for generic delimitation. Among the three new genera described herein, *Parapenioides*, *Sinopenia*, and *Megapenia* also possess sclerotized bursal structures, but these differ markedly from those found in *Parapenia*. Notably, most *Megapenia* species (e.g., *M. marginalis*, *M. cruciata*) exhibit well-developed sclerites, while *M. tianlongi* shows evidence of structural reduction.

In summary, female bursa copulatrix sclerites in Dimini are morphologically diverse and can be classified into four major types:

(1) Absent, as in *Sinodima*, *Neodima*, and *Neocsikia*;
(2) Well developed, as in *Parapenia*, *Platiana*, *Parapenioides*, and *Sinopenia*;
(3) Well developed, but with indications of reduction in some species, as in *Megapenia*;
(4) Generally absent but present as small spiniform elements in certain species or regional populations, as seen in *Dima* (Balkan species) and *Penia* (*P. takasago*).

Additionally, female genitalia remain undescribed for several genera, including *Csikia*, *Paracsikia*, *Sabahdima*, and *Pseudocsikia*, leaving the presence of bursal sclerites in these taxa unresolved. Even in species-rich genera such as *Penia*, *Platiana*, and *Dima*, previously undetected forms may yet be discovered, as most species remain unexamined for female internal structures. Expanded anatomical investigations of female specimens will be critical to fully uncover the diversity and evolutionary patterns of bursal sclerotization within the tribe Dimini.

### 4.2. Taxonomic Tangles of the Study on Parapenia

Although this study provides a systematic revision of *Parapenia* species from China, species delimitation remains challenging for several dull-colored and morphologically similar taxa, including *P. tonkinensis*, *P. yunnana*, *P. taiwana*, *P. wulingshanensis*, and *P. villosa*. The difficulty lies not only in their inconspicuous external features, but more critically in the high morphological variability and limited diagnostic value of the aedeagus.

Extensive dissections conducted in the present study reveal a striking degree of interspecific similarity in aedeagal morphology (Figure 4D, Figure 6D, Figure 8D, Figure 11G, Figure 13D, Figure 16H–Q, Figure 19G–M, Figure 23D,I and Figure 24H), indicating a notable trend of convergence across species. Concurrently, pronounced intraspecific variation is observed (e.g., in *P. tonkinensis*: Figure 16H–Q; and in *P. villosa*: Figure 19G–M), with even individuals from the same locality often displaying substantial differences in the shape of the phallobase and the apical portion of the parameres. Some specimens exhibit highly divergent or even extreme aedeagal morphologies that, in isolation, could justify their treatment as separate species; however, their external morphology is indistinguishable from that of sympatric individuals (e.g., *P. villosa* from Tam Dao: specimen of Figure 17I and Figure 19I versus that of Figure 17F–H,J and Figure 19J). Among all examined taxa, *P. wulingshanensis* is the only species exhibiting aedeagal features that are consistently distinguishable from its congeners. Nevertheless, considerable intraspecific variation persists in this species as well, particularly in the form of the paramere apex and the shape of the phallobase (Figure 22H–M).

These findings highlight a general pattern in *Parapenia*: notable interspecific similarity paired with extensive intraspecific variability in male genitalia, thus substantially limiting the utility of these characters for reliable species identification. Future taxonomic work incorporating fresh material and molecular approaches, such as DNA barcoding or phylogenetic analysis, will be essential for resolving these ambiguities.

## Figures and Tables

**Table 1:** Comparative characters of Dimini genera with large sclerotized plates in bursa copulatrix. Abbreviations used are explained directly in the table below.

Characters	*Parapenia*	*Parapenioides*	*Sinopenia*	*Megapenia*
Body length (mm)	8.3–11.3	11.1–12.2	10.9–12.5	11.3–19.1
Body shape	Stout to elongate	Elongate	Elongate	Elongate
Anterior angle of pronotum (AAP)	Narrowly protruded (Figure 25A)	Right-angled (Figure 25D)	Subquadrate (Figure 25G)	Laterally arched (Figure 25J)
Pit at base of AAP	Deep, hole-like	Indistinct	Lunate fossa	Shallowly impressed
Posterior angle of pronotum (PAP)	Stout, bidentate	Elongate, weakly bidentate	Elongate, weakly bidentate	Elongate, unidentate
Small notch at posterior margin of PAP	One to two, shallow to deep	One, shallow	One, shallow	One, deep
Lateroapical setae on PAP	Absent	Absent	Absent	Present
Long curved setae on tarsomere IV	Weak	Weak	Weak	Strong
Prosternal process (PP) (lateral view)	Slightly narrowed	Slightly narrowed	Widened, with large ventro-apical notch	Strongly narrowed
ventro-apical notch of PP	small to large	small to large	large	small
Anterior angle of elytra	Bidentate	Bidentate	Bidentate	Rounded
Median lobe and parameres	Slender	Slender	Stout	Stout
Spiculum ventrale/sternite VIII length ratio	3.0–3.5	3	2.7	1.4–2.8
Paraproct/coxite length ratio	4.9-5.8	4.8	4.6	2.2-4.0
sclerites of bursa copulatrix	1 large fan-like structure and 2 robust plates (Figure 4H)	2 large plates (Figure 27H)	1 large weakly sclerotized region (Figure 29H)	1 large and 2 small plates; or obsolete (Figure 34H)

## Data Availability

The original contributions presented in this study are included in the article. Further inquiries can be directed to the corresponding authors.
